# Psychotropic medications and their interactions with subcortical brain volume in bipolar disorder: An ENIGMA mega-analysis

**DOI:** 10.1038/s41380-025-03432-z

**Published:** 2026-01-15

**Authors:** Sinead King, John O’Connor, Emma Corley, Giulia Tronchin, Elisa Fontana, Leila Nabulsi, Melody J. Y. Kang, Joaquim Radua, Brian Hallahan, Christoph Abé, Martin Alda, Dag Alnæs, Silvia Alonso-Lana, Silvia Amoretti, Jochen Bauer, Francesco Benedetti, Klaus Berger, Michael Berk, Erlend Bøen, Joscha Böhnlein, Birgitte Boye, Beatrice Bravi, Erick J. Canales-Rodríguez, Udo Dannlowski, Caroline Demro, Annabella Di Giorgio, Ana M. Diaz-Zuluaga, Torbjørn Elvsåshagen, Pauline Favre, Tracy Erwin-Grabner, María Florencia Forte, Janice M. Fullerton, Lisa S. Furlong, Susan L. Rossell, David C. Glahn, Benjamin I. Goldstein, Ian H. Gotlib, Roberto Goya-Maldonado, Melissa J. Green, Dominik Grotegerd, Oliver Gruber, Bartholomeus C. M. Haarman, Tim Hahn, Tomas Hajek, Leonie Hater, Marco Hermesdorf, Josselin Houenou, Fleur M. Howells, James A. Karantonis, Kody G. Kennedy, Tilo Kircher, Anna Luisa Klahn, Maximilian Konowski, Bernd Krämer, Elijah Lahud, Rayus Kuplicki, Mikael Landén, Carlos López-Jaramillo, Bradley J. MacIntosh, Hannah Meinert, Susanne Meinert, Elisa M. T. Melloni, Philip B. Mitchell, Benson Mwangi, Igor Nenadić, Bronwyn J. Overs, Nadine Parker, Godfrey Pearlson, Edith Pomarol-Clotet, James J. Prisciandaro, Yann Quidé, Gloria Roberts, Amanda Rodrigue, Elena Rodríguez-Cano, Lisa Rauer, Matthew D. Sacchet, Raymond Salvador, Fabio Sambataro, Theodore D. Satterthwaite, Jonathan Savitz, Freda Scheffler, Navid Schürmeyer, Chen Shen, Kang Sim, Jair C. Soares, Aleix Solanes, Márcio Gerhardt Soeiro-de-Souza, Scott R. Sponheim, Dan J. Stein, Frederike Stein, Henk S. Temmingh, Lea Teutenberg, Sophia I. Thomopoulos, Snezana Urosevic, Tamsyn E. Van Rheenen, Eduard Vieta, Lars T. Westlye, Daniel H. Wolf, Mon-Ju Wu, Lakshmi N. Yatham, Giovana B. Zunta-Soares, Dara M. Cannon, Paul M. Thompson, Ole A. Andreassen, Christopher R. K. Ching, Colm McDonald

**Affiliations:** 1 Clinical Neuroimaging Laboratory, Centre for Neuroimaging, Cognition & Genomics (NICOG), College of Medicine Nursing and Health Sciences, University of Galway, Galway, Ireland; 2 Research Ireland Futureneuro Centre for Translation Brain Science, Royal College of Surgeons, Dublin, Ireland; 3 Foundation IRCCS Ca’ Granda Ospedale Maggiore Policlinico, Department of Neurosciences and Mental Health, Milan, Italy; 4 Imaging Genetics Center, Mark and Mary Stevens Neuroimaging and Informatics Institute, Keck School of Medicine, University of Southern California, Los Angeles, California, USA; 5 Institut d’Investigacions Biomèdiques August Pi i Sunyer (IDIBAPS), Centro de Investigación Biomédica en Red de Salud Mental (CIBERSAM), Barcelona, Spain; 6 Department of Medicine, Faculty of Medicine and Health Sciences, Institute of Neurosciences (UBNeuro), University of Barcelona, Barcelona, Spain; 7 Department of Clinical Neuroscience, Karolinska Institutet, Stockholm, Sweden; 8 Department of Psychiatry, Dalhousie University, Halifax, Nova Scotia Canada; 9 Department of Psychology, University of Oslo, Oslo, Norway; 10 Centre for Precision Psychiatry, University of Oslo, Oslo, Norway; 11 FIDMAG Germanes Hospitalàries Research Foundation, Barcelona, Spain; 12 CIBERSAM, ISCIII, Madrid, Spain; 13 Bipolar and Depressive Disorders Unit, Department of Psychiatry and Psychology, Hospital Clínic de Barcelona, Barcelona, Spain; 14 Psychiatry, Mental Health and Addictions Group, Vall d’Hebron Research Institute (VHIR), Instituto de Investigación Sanitaria Acreditado Instituto de Investigación - Hospital Universitario Vall d’Hebron (IR-HUVH), Centro de Investigación Biomédica en Red de Salud Mental, Instituto de Salud Carlos III, Barcelona, Catalonia Spain; 15 Department of Radiology, University of Münster, Münster, Germany; 16 Vita‐Salute San Raffaele University, Milan, Italy; 17 Division of Neuroscience, Psychiatry and Psychobiology Unit, IRCCS Ospedale San Raffaele, Milan, Italy; 18 Institute of Epidemiology and Social Medicine, University of Münster, Münster, Germany; 19 Deakin University, IMPACT Institute – The Institute for Mental and Physical Health and Clinical Translation, School of Medicine, Barwon Health, Geelong, Victoria Australia; 20 Institute of Clinical Medicine, University of Oslo, Oslo, Norway; 21 Department of Psychosomatic Medicine, Oslo University Hospital, Oslo, Norway; 22 Institute for Translational Psychiatry, University of Münster, Münster, Germany; 23 Clinical Psychology and Translational Psychotherapy, Department of Psychology, University of Münster, Münster, Germany; 24 Oslo University Hospital, Department of Acute Psychiatry, Unit of Psychosomatic and CL Adult Psychiatry, Oslo, Norway; 25 Department of Behavioural Medicine, Institute of Basic Medical Sciences, University of Oslo, Oslo, Norway; 26 Signal Processing Lab (LTS5), École Polytechnique Fédérale de Lausanne, Lausanne, Switzerland; 27 Department of Radiology, Centre Hospitalier Universitaire Vaudois (CHUV), Lausanne, Switzerland; 28 Department of Psychiatry, Medical School and University Medical Center OWL, Protestant Hospital of the Bethel Foundation, Bielefeld University, Bielefeld, Germany; 29 Department of Psychology, University of Minnesota, Minneapolis, MN USA; 30 Department of Psychiatry and Behavioral Sciences, University of Minnesota, Minneapolis, MN USA; 31 Department of Mental Health and Addictions, ASST Papa Giovanni XXIII, Bergamo, Italy; 32 Research Group in Psychiatry GIPSI, Department of Psychiatry, Faculty of Medicine, Universidad de Antioquia, Medellín, Colombia; 33 Department of Neurology, Oslo University Hospital, Oslo, Norway; 34 Section for Precision Psychiatry, Division of Mental Health and Addiction, Oslo University Hospital, Oslo, Norway, Oslo, Norway; 35 Université Paris Est Créteil, INSERM Unit 955, Team Translational NeuroPsychiatry, Créteil, France; 36 Neurospin, CEA Paris‐Saclay, team UNIACT, Gif‐sur‐Yvette, France; 37 Laboratory of Systems Neuroscience and Imaging in Psychiatry, Department of Psychiatry and Psychotherapy, University Medical Center Goettingen, Goettingen, Germany; 38 Neuroscience Research Australia, Randwick, New South Wales Australia; 39 School of Biomedical Sciences, Faculty of Medicine and Health, University of New South Wales, Sydney, Australia; 40 Department of Psychiatry, University of Melbourne, Melbourne, Victoria Australia; 41 Centre for Mental Health and Brain Sciences, School of Health Sciences, Swinburne University, Melbourne, Australia; 42 St Vincent’s Mental Health, St Vincent’s Hospital, Melbourne, VIC Australia; 43 Department of Psychiatry, Boston Children’s Hospital, Boston, MA USA; 44 Department of Psychiatry, Harvard Medical School, Boston, MA, Boston, USA; 45 Centre for Youth Bipolar Disorder, Centre for Addiction and Mental Health, Toronto, Canada; 46 Department of Pharmacology and Toxicology, University of Toronto, Toronto, Canada; 47 Department of Psychiatry, University of Toronto, Toronto, Canada; 48 Department of Psychology, Stanford University, Stanford, CA USA; 49 Discipline of Psychiatry and Mental Health, School of Clinical Medicine, Faculty of Medicine and Health, University of New South Wales, Sydney, Australia; 50 Section for Experimental Psychopathology and Neuroimaging, Department of General Psychiatry, Heidelberg University, Heidelberg, Baden-Württemberg Germany; 51 Department of Psychiatry, University Medical Center Groningen, University of Groningen, Groningen, The Netherlands; 52 National Institute of Mental Health, Klecany, Czech Republic; 53 Northern Hospital, Mental Health Services, Epping, Victoria, Australia; 54 APHP, Mondor University Hospitals, DMU IMPACT, Créteil, France; 55 Neuroscience Institute, University of Cape Town, Cape Town, South Africa; 56 Department of Psychiatry and Mental Health, University of Cape Town, Cape Town, South Africa; 57 Philipps-Universität Marburg, Faculty of Medicine, Department of Psychiatry and Psychotherapy, Rudolf-Bultmann- Str. 8, D-35039 Marburg, Germany; 58 Department of Psychiatry and Neurochemistry, Institute of Neuroscience and Physiology, University of Gothenburg, Gothenburg, Sweden; 59 Minneapolis VA Health Care System, Minneapolis, MN USA; 60 Laureate Institute for Brain Research, Tulsa, Oklahoma USA; 61 Department of Medical Epidemiology and Biostatistics, Karolinska Institutet, Stockholm, Sweden; 62 Department of Medical Biophysics, University of Toronto, Toronto, Canada; 63 Institute for Translational Neuroscience, University of Münster, Münster, Germany; 64 Center of Excellence on Mood Disorders, UTHealth Houston, Houston, TX USA; 65 Department of Psychiatry and Behavioral Sciences, UTHealth Houston, Houston, TX USA; 66 School of Behavioral Health Sciences, UTHealth Houston, Houston, TX USA; 67 Departments of Psychiatry and Neuroscience, Yale University School of Medicine, New Haven, CT USA; 68 Department of Psychiatry and Behavioral Sciences, Medical University of South Carolina, Charleston, SC USA; 69 NeuroRecovery Research Hub, School of Psychology, University of New South Wales (UNSW) Sydney, Sydney, New South Wales Australia; 70 Meditation Research Program, Department of Psychiatry, Massachusetts General Hospital, Harvard Medical School, Boston, MA USA; 71 Department of Neuroscience, University of Padova, Padova, Italy; 72 Department of Psychiatry, University of Pennsylvania Perelman School of Medicine, Philadelphia, PA USA; 73 Oxley College of Health Sciences, The University of Tulsa, Tulsa, Oklahoma USA; 74 West Region, Institute of Mental Health, Singapore, Singapore; 75 Yong Loo Lin School of Medicine, National University of Singapore, Singapore, Singapore; 76 Mood Disorders Unit (GRUDA), Hospital das Clinicas HCFMUSP, Faculdade de Medicina, Universidade de São Paulo, São Paulo, SP Brazil; 77 South African Medical Research Council Unit on Risk & Resilience in Mental Disorders, South African Medical Research Council, Cape Town, South Africa; 78 Valkenburg Hospital, Cape Town, South Africa; 79 KG Jebsen Centre for Neurodevelopmental Disorders, University of Oslo, Oslo, Norway; 80 Division of Mental Health and Addiction, Oslo University Hospital, Oslo, Norway; 81 Department of Psychiatry, University of British Columbia, Vancouver, British Columbia Canada; 82 Departments of Neurology, Psychiatry, Radiology, Engineering, Pediatrics and Ophthalmology, University of Southern California, Los Angeles, CA USA

**Keywords:** Neuroscience, Bipolar disorder

## Abstract

MRI studies in bipolar disorder (BD) have yielded inconsistent findings, partly due to the varied use of psychotropic medications. This study utilised a mega-analysis approach, accounting for concurrent medication status (syndrome-based and Neuroscience-based Nomenclature (NbN) classifications), in order to assess the association of medication status with subcortical brain volumes in BD. Data from 2,664 BD patients and 4,065 controls (CN) were pooled from 34 research groups as part of the ENIGMA Bipolar Disorder Working Group. Standardized ENIGMA protocols were used to measure subcortical brain volumes. Linear-mixed-effects regression evaluated the association between psychotropic medications and subcortical volumes, and moderation analyses explored interactions. Medication-free patients (n = 410) showed mild ventricular enlargement (d = 0.07) and increased putamen volume (d = 0.06) compared to CN. Patients taking psychotropic medications exhibited smaller subcortical volumes (d = -0.06 to -0.11) and larger ventricles (d = 0.11 to 0.19). Use of antiepileptic and antipsychotic medications was associated with smaller hippocampal and thalamic volumes (d = -0.07 to -0.14), while NbN classification indicated that the categories of ‘valproate’ and ‘dopamine and other monoamine receptor antagonists’ are key variables when considering volume differences between BD and CN. Concurrent lithium use weakened the negative association between antiepileptic use and hippocampal volume (β = 0.19, q = 0.038) in patients. Medication status is associated with altered subcortical brain volumes in BD. The NbN classification provides a useful framework for future studies, emphasizing the need for comprehensive longitudinal research to further unravel complex clinical-pharmacological-neurobiological interactions in BD.

## Introduction

Neuroimaging studies of bipolar disorder (BD) have reported volumetric alterations in various subcortical brain regions, but results are inconsistent, likely due to clinical and methodological heterogeneity, as well as underpowered sample sizes [1]. Among the more consistent differences to be documented in prior meta-analyses and large-scale mega-analyses of subcortical volumes are larger ventricles and smaller hippocampal and thalamic volumes [2, 3] in patients with BD relative to controls (CN).

Studies have varied in the neuroimaging processing, statistical methodology, and analysis techniques used and in the choice of brain volumes assessed. These differences have led to discrepancies in the reported patterns of alterations in brain structure and function. International clinical research collaborations such as the Enhancing Neuro Imaging Genetics through Meta-Analysis (ENIGMA) Consortium can overcome some of these limitations by pooling data and applying standardized processing pipelines to maximize sample size and diversity [1]. The ENIGMA Bipolar Disorder Working Group (ENIGMA-BD) was formed in 2012 and has published the largest neuroimaging studies of BD to date [4], pooling existing neuroimaging samples to build the most geographically diverse samples of BD and CN, leading to more ecologically valid cohorts that are more likely to generalize to patient populations.

Patients with BD are commonly prescribed more than one psychotropic medication. A key consideration when reviewing prior literature on neuroanatomical alterations in BD is the extent to which the concurrent use of psychotropic medications, such as lithium (Li) and antipsychotics (AP), may influence these brain differences. Preclinical and clinical studies suggest that Li may have neurotrophic effects [5], whereas AP medications have been associated with smaller gray and white matter volumes [6–8]. Supporting this, Hajek et al. [9] demonstrated that BD patients on Li had larger total hippocampal volumes compared to those not taking Li, a finding consistent with several other studies [2, 10–14]. Similarly, our prior ENIGMA-BD analyses showed that patients taking Li had a widespread pattern of thicker cortex and larger thalamic volumes compared to those not taking Li [3, 15].

Deciphering the impact of psychotropic medications on brain anatomy in vivo however, is further potentially complicated by interacting effects of illness severity and duration, polypharmacy, genetic and environmental risk factors, obesity, and substance misuse [16, 17]. Furthermore, prior studies have used the traditional syndrome-based classification of psychotropic drugs which uses nomenclature such as antidepressants (AD) and antiepileptic mood stabilizers (AED), as opposed to classifications that reflect the underlying neuropharmacological mechanism of action of these compounds (e.g., primarily dopamine receptor antagonists), which may provide more predictive power when modeling their associations with brain volume variations. The European College of Neuropsychopharmacology has created such a pharmacologically-driven classification system, the Neuroscience-based Nomenclature (NbN), with the goal of moving away from syndrome-based classification to a pharmacologically driven classification [18].

Given the significant role of subcortical limbic structures in BD and our previous ENIGMA-BD meta-analysis findings, we aimed to elucidate the associations between psychotropic medication status and subcortical brain structure in an expanded cohort (56% larger than in previous analyses [3]). To address the complexities of medication effects, we employed both traditional syndrome-based classifications and the NbN approach, which considers the pharmacological mechanisms of action. We focussed primarily on medication status and its associations, while accounting for potential confounds, including concurrently prescribed psychotropic medications, and incorporated detailed medication information. We hypothesized that BD patients taking different classes of psychotropic medications would show distinct patterns of subcortical volume alterations compared to controls and to those not taking such medications, and that these patterns would further differ based on the pharmacological mechanisms of action. Given the role of subcortical structures in mood regulation and BD pathophysiology [19], a richer understanding of these associations is critical for developing better clinical interventions. It is important to note that a true causal relationship between medication status and brain structural variation cannot be definitively established from cross sectional observational studies such as this. Brain morphometric variation may be a cause rather than a consequence of medication selection and large scale randomised clinical trials would be necessary to disentangle the direction of effect. Nevertheless, identifying morphometric associations with medication status could provide valuable insights into potential mechanisms underlying psychotropic medications and their associations with brain structure and function.

The specific research questions addressed were:

1. Do subcortical volumes of patients with BD taking no medications and of those taking one or more concurrent psychotropic medications differ from CN?

2. Are subcortical brain volumes associated with medication status, when classified based either on traditional syndrome-based categories or on their pharmacological mechanisms of action, when comparing patients with BD taking a specific medication class to CN or to patients who are not using the specific medication class?

3. Does taking Li interact with taking other psychotropic medications to influence subcortical brain volume?

4. Does medication status moderate the association between clinical indicators of illness course and subcortical volumes?

## Methods & materials

### Participating sites

We included ENIGMA-BD samples from 34 independent sites for this project (Supplementary Table 1), comprising 6,729 participants (2,664 BD patients and 4,065 CN). Of the 2,664 BD patients, medication status was unavailable for 251 participants. Each participating site obtained approval from an institutional review board or local ethics committee. All study participants completed informed consent documents prior to their original participation. Supplementary Table 2 provides the instruments used to obtain diagnosis and clinical information for each site. Supplementary Table 3 lists exclusion criteria for study enrolment. Most studies did not exclude comorbidities, other than substance abuse. All procedures contributing to this work comply with the ethical standards of the relevant national and institutional committees and all methods were performed in accordance with the relevant guidelines and regulations.

### Data acquisition and parcellation

Structural 3D T1-weighted brain MRI scans were acquired at each site. Detailed information on scanner sequence and acquisition parameters are presented in Supplementary Table 4. All sites used the same ENIGMA-standardized analytical protocols documented at: http://enigma.ini.usc.edu/protocols/imaging-protocols/. These protocols are standardized across the ENIGMA Consortium, are open-source, and are freely available online in order to foster open science, replication, and reproducibility. The protocols have been applied in prior publications by ENIGMA-BD [3, 17, 20], and more broadly across the ENIGMA Consortium [1]. Briefly, FreeSurfer was used to derive volumes of eight subcortical regions (lateral ventricles, nucleus accumbens, amygdala, hippocampus, pallidum, putamen, caudate nucleus, and thalamus), per hemisphere (left and right), based on the FreeSurfer subcortical atlas [21]. Each site’s data underwent a rigorous quality control process, which included the exclusion of images with significant artifacts or poor quality, and the standardization of preprocessing steps, such as skull stripping, spatial normalization, and intensity normalization. Bilateral brain volumes were used to index brain structure of each region, by deriving an average volume from both hemispheres. Volumes from one hemisphere occasionally failed the quality control stage. In these cases, we instead used the volumes that were present for the other hemisphere to minimise data loss. For transparency, we also conducted *a posteriori* analysis of left and right hemisphere volumes separately to ensure consistent findings across both hemispheres for the primary analyses. Measures of total intracranial volume (ICV) were computed to standardize volume estimates. A description of image and volume segmentation quality control is presented in Supplementary Note 1.

### Categorisation of medications

Medications used at the time of scan were first categorised using the traditional, syndrome-based classes (i.e., Li, AP, AD, AED). We combined first- and second-generation AP because this dichotomy is not currently justified pharmacologically [22], and they have similar associations with reduced brain volumes in primate models [23]. Subsequently, we used more detailed named medication information to reclassify medication class by their pharmacological domain and mechanism of action using the Neuroscience-based Nomenclature (NbN), described here: https://nbn2r.com/ and in Supplementary Note 2 and Supplementary Tables 5a-c. This resulted in a final set of 10 NbN-based categories (Supplementary Table 5c). AP were recoded into three NbN categories: primarily dopamine receptor antagonists, dopamine and other (serotonin-norepinephrine) monoamine receptor antagonists, and dopamine, serotonin receptor partial agonist/antagonists. AD were recoded into two NbN categories: those targeting primarily serotonin (reuptake inhibitors - multimodal) and those targeting serotonin and other monoamines with different mechanisms of action. AED were recoded into glutamate, sodium/calcium channel blockers and valproate. Both Li and valproate were placed in their own individual NbN categories because their mechanism of action remains unclear. Anxiolytics and hypnotics were recoded into the category GABA positive allosteric modulators. Psychotropic medications for which the mechanism of action is either still unclear or the drug is not yet listed in NbN were recoded in an “Other” category and were not included in the analyses because of the multiple medications included with diverse mechanisms of action. Considering that Li was analysed in the traditional medication classification, this was not repeated for the NbN analysis. Thus, the NbN analysis was conducted on the remaining 8 categories.

### Statistical analysis

All individual-level subcortical volume, clinical and demographic data were pooled centrally to perform a ‘mega-analysis’ comparing subcortical and ventricular volumes between BD and CN. We used linear mixed modelling (package *lme4*, version 3.1-152 in R ([24], version 4.1.1) to examine associations between subcortical volumes and pharmacological treatment. We opted to use linear mixed models as they allow for both fixed and random effects and are powerful when dealing with hierarchically structured data. In addition, linear mixed models are robust in handling missing data and unequal group sizes, and allowed for including site as a random effect to account for residual variance attributable to site-specific differences. For these analyses, regional brain volume was the dependent variable, and each medication class was the independent fixed factor, with age, sex, ICV and other medications taken added as covariates. Scan site was included as a random factor. To initially assess overall medication load, we compared those taking no medication (N = 410) and those taking one, two or three or more (any combination) medications at the time of scan to CN. Next, we ran one model per analysis for each medication category, such that e.g., ‘Li’ as the independent fixed factor was replaced with ‘AED’ (and so on), for all traditional syndrome-based medication categories. Our covariates for each model were age, sex, ICV and other medications taken. We then ran individual models for medications reclassified under the NbN mechanism of action categories. In total, we ran 144 linear mixed models. As an additional *a posteriori analysis*, we performed linear mixed models separately for the left and right subcortical volumes for the syndrome-based classification to ensure that the findings were consistent across each hemisphere, and posthoc sensitivity analyses including propensity score matching to assess the impact of medication use and polypharmacy on subcortical volumes when accounting for measures of clinical severity. See Supplementary Note 3 for a detailed description of statistical analysis techniques.

Moderation analysis was run using PROCESS 4.3.1 [25] in the *bruceR* package (version 2023.9) to assess whether the observed significant associations described above in patients (i.e. of each medication class on brain volume) were significantly influenced by Li use when co-prescribed. The moderation analysis was intended to explore potential interactions rather than establish causality. We chose to focus on Li as a moderator due to its proposed neuroprotective effects reported in the preclinical and clinical literature and due to the results reported in the present study, with a particular interest in whether concurrent Li use can attenuate the negative associations of other psychotropic medications with subcortical volume. In an exploratory analysis, we examined whether differences in medication status in BD and clinical course indicators were associated with significant subcortical volumes from the linear mixed models. All analyses were adjusted for age, sex, and ICV, with data clustering effects across each site. In each moderation analysis, we controlled for the effects of medications other than the one serving as the moderator variable (i.e., when examining the moderating effect of Li use on the association between AED and subcortical brain volume, AP and AD were included as covariates in the model).

We corrected for multiple comparisons across all 237 models (See Supplementary Note 3) using the Storey et al. [26] false discovery rate (FDR) procedure in the *qvalue* package (R package version 2.34.0), whereby q-values < 0.05 were considered statistically significant. This approach was chosen to control for the potential inflation of Type I error across a large number of comparisons and is an extension of the traditional Benjamini-Hochberg procedure. We considered q-values < 0.05 as the threshold for statistical significance. By correcting across all models, we aimed to ensure a robust and consistent control of false discovery rates across the entire set of analyses. We calculated effect sizes from the t-statistic (Cohen’s d) and their 95% confidence intervals (CIs) using the *effectsize* package in R.

## Results

Descriptive information on the demographic and clinical variables of patients divided into medication subgroups is provided in Table 1. When comparing the entire BD cohort irrespective of medication status to CN, BD patients displayed enlarged ventricles and smaller hippocampus, thalamus and ICV (See Supplementary Figure 1 and Supplementary Table 6). Across all brain regions, less than 5% of cases were affected by missing data when only one hemisphere passed quality control (Supplementary Table 7). To confirm that this minor data loss did not bias results or introduce asymmetry, we repeated the analyses using linear mixed models that accounted for both left and right subcortical volumes and demonstrated similar results (Supplementary Table 8).

**Table 1:** Demographic and Clinical Information.

		Healthy Controls (CN) (n=4065)		Patients (BD) (n=2664)		t/z/x2 value	*p*-value	Patients not taking medication (n=410)	Patients taking one medication class (n=764)	Patients taking two medication classes (n=877)	Patients taking three medication classes (n=362)	F/H/x^2^	*p*-value	Patients taking Li (n=870)	Patients taking AED (n=925)	Patients taking AP (n=1008)	Patients taking AD (n=840)
*Demographic and clinical information*	*N CN/BD*	*M*	*St.D*	*M*	*St.D*			*M(SD)/%*	*M(SD)/%*	*M(SD)/%*	*M(SD)/%*			*M(SD)/%*	*M(SD)/%*	*M(SD)/%*	*M(SD)/%*
Age at time of scan	4051 (CN)/ 2663 (BD)	33.3	12.6	37	13.7	-11	<0.0001	33.7 (13.6)	36.8 (14)	37.5 (13.5)	40.8 (12.8)	17.86	<0.0001	39.8 (13.2)	38.2 (13.2)	36.4 (13.6)	39.6 (13.6)
Sex	4065 (CN)/ 2664 (BD)	2268 (56%) female		1548 (58%)female		3.4	.063	248 (60%)female	446 (58%)female	506 (58%)female	222 (61%)female	1.92	0.589	481 (55%)female	567 (61%)female	566 (56%)female	528 (63%)female
Serum levels at time of scan (mmol/L)	155 (Li)	..		..		..	..	..	..	..			..	0.59 (0.19)	..		
Concurrent medication status (N & %)	2413 (BD)	..	..	0 =410 (15%), 1=764 (29%), 2=877 (33%), 3>=362 (14%)		..	..		Li=246 (32%), AED=178(23%), AP=187 (25%), AD=153 (20%)	Li=375 (43%), AED=471(54%), AP=510 (58%), AD=398 (45%)	Li=249 (69%), AED=276(76%), AP=311 (86%), AD=289 (80%)	..	..	..	..	..	
BDI/BDII/BDNOS	2264	..	..	BDI =1638; BDII=518; BDNOS=108		..		235/138/31/6	480/185/32/67	591/144/21/121	262/37/6/57	88.76	<0.0001	625/97/15/134	621/161/18/125	713/115/31/149	512/216/28/84
Body mass index (BMI)	1826 (CN)/ 1448 (BD)	24	4.3	26.6	5.8	-13.7	<.00001	25.9 (6.1)	26 (5.5)	26.7 (5.8)	28.1 (6.1)	28.72	<0.0001	26.6 (5.2)	27.2 (6.2)	27.1 (6)	27.3 (6.1)
Education in years	2178 (CN)/ 1214 (BD)	14.9	3	14.2	3.7	-5.1	<0.0001	13.9 (3.6)	14.3 (3.9)	14.3 (3.4)	13.9 (3.6)	2.41	0.485	14.4 (3.5)	14.1 (3.6)	14.1 (3.7)	14.1 (3.6)
Full scale IQ	1868 (CN)/821 (BD)	115	13.5	106	17.3	-12.2	<0.0001	108.3 (16.7)	108.2 (16.3)	104.6 (17.4)	105 (19.3)	5.79	0.121	105.4 (16.4)	104.6 (18.6)	103.9 (18.3)	109.1 (17.4)
CTQ Total	1252 (CN)/ 343 (BD)	32.4	9.7	40.4	17.9	-10.3	<0.0001	35.8 (19.2)	37.4 (18.5)	42.3 (16.4)	44.2 (17.5)	8.61	0.035	42.1 (14.1)	41.1 (19.1)	43.1 (16.5)	41.8 (18.3)
*Illness course measures*																	
History of Psychosis	1771 (BD)	..	..	778 (29.2%)		..		84 (20.1%)	214 (28%)	309 (35.2%)	155 (42.8%)	53.99	<0.0001	338 (38.9%)	356 (38.5%).	388 (38.5%)	234 (27.9%)
Illness Duration (in years)	2036 (BD)	..	..	15.3	11.9	..	..	13.6 (10.7)	14.1 (11.1)	14.7 (11.3)	16.3 (11)	14.03	0.0003	15.8 (11.2)	15.3 (11)	13.6 (11.1)	16.1 (11.2)
Age of Onset (in years)	2036 (BD)	..	..	22.8	10.2	..	..	20.3 (10.5)	22.6 (10)	23.2 (10)	25 (10.7)	55.01	<0.0001	24.2 (9.8)	23.4 (10.3)	23.1 (10.3)	23.9 (10.6)
Psychiatric Hospitalisations	611 (BD)	..	..	2.5	4.3	..	..	0.9 (2.7)	2.3 (2.5)	3.4 (4.4)	4.6 (6.3)	129.41	<0.0001	3.8 (3.4)	3.4 (4.2)	3.6 (4.3)	3.4 (4)
Total hypomanic episodes	949 (BD)	..	..	6.1	16.7	..	..	5.4 (12.6)	4.7 (12.8)	3.6 (10.4)	2 (6.4)	13.72	0.0033	2.4 (7.2)	4.4 (12)	2.6 (9.4)	3.8 (10.2)
Total manic episodes	1356 (BD)	..	..	8.7	20.8	..	..	7.4 (18.4)	7.9 (19.2)	6.9 (18.4)	6.9 (16.8)	9.7	0.021	5.3 (13.8)	8 (19.5)	7.2 (18.2)	8 (20.3)
Total depressive episodes	14319 (BD)	..	..	12.3	21.5	..	..	11.3 (20.7)	11.3 (20.5)	10.7 (18.9)	11.2 (17.8)	5.14	0.167	8.8 (15.2)	12.5 (20.9)	9.5 (17.3)	13.3 (21.1)
Total mixed episodes	742 (BD)	..	..	2.3	11.1	..	..	0.6 (3.3)	0.3 (1.7)	0.7 (3)	0.4 (2.4)	2.15	0.541	0.3 (1.7)	0.7 (3.2)	0.6 (3.1)	0.4 (2.3)
BDI Total	826 (CN)/ 1448 (BD)	2.9	3.6	16.1	13	-18.1	<0.0001	19.2 (13)	13.8 (13.1)	17.3 (13.7)	15.8 (10.4)	7.93	0.047	10.1 (10.2)	16.4 (13.5)	17.2 (13)	19.6 (11.2)
HDRS Total	1492 (CN) /795 (BD)	1.1	2	12.1	9	-31.7	<0.0001	12 (8.3)	11.8 (9.6)	12.8 (9)	14.6 (8.4)	9.42	0.024	12.3 (9.8)	13.4 (8.8)	12.1 (8.5)	14.8 (8.6)
MADRS Total	894(CN)/ 625(BD)	1.9	5.2	12.4	10.7	-24.5	<0.0001	14.3 (11.1)	11 (11.1)	11.7 (10)	12.8 (10.6)	10.49	0.015	11.1 (10.3)	10.5 (9.7)	11.7 (10.2)	14 (11.3)
CES-D Total	44(CN)/ 92(BD)	6.7	4.2	13.6	15	-0.4	0.645	..	16.25 (10.2)	22.75 (14.6)	28.2 (10.6)	3.82	0.28	27.3 (10.7)	25.7 (14.2)	23.9 (12.4)	26.7 (12.4)

*t* t-statistic, *z* Mann-Whitney *z*-value (t and z were calculated for differences between BD and CN), F = F-statistic, *H* Kruskal-Wallis H statistic, *χ²* Chi-Square statistic (F, H and χ² were calculated for differences between BD patients not taking, taking one class, taking two classes, and taking three or more classes of medication), *Li* Lithium, *AED* Antiepileptics, *AP* Antipsychotics, *AD* Antidepressants, *BMI* Body Mass Index, *CTQ* Childhood Trauma Questionnaire, *BDI* Beck’s Depression inventory, *HDRS* Hamilton Depression Rating Scale, *MADRS* Montgomery-Asberg Depression Rating Scale, *CES-D* Center for Epidemiological Studies Depression. Note that in the *N CN/BD* column, the number of CN and BD does not match the total number of CN and BD as this data was missing/not collected for some sites included in this study.

### Patients with BD taking no medications or on one or more concurrent medications

**Fig. 1:**
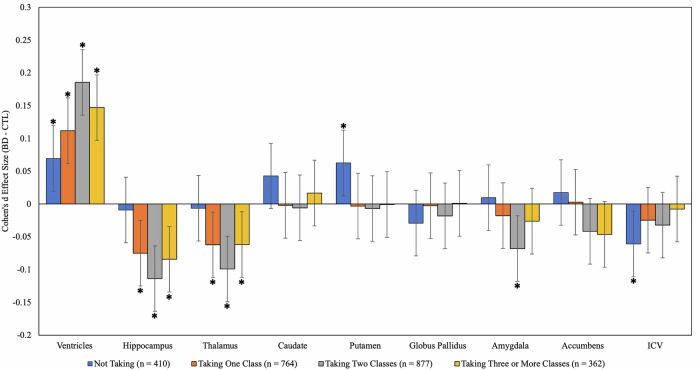
Adjusted effect size estimates for BD patients not taking and those taking one or more classes of psychotropic medication at the time of scan compared with CN. For the ‘taking one’ group, patients were taking any one of the four syndrome-based medications (e.g., Lithium, Antiepileptics, Antipsychotics, Antidepressants). For the ‘taking 2’ and ‘taking 3 or more’ groups, patients were taking any combination of the syndrome-based medications. Effect sizes are reported after accounting for age, sex, intracranial volume and other medications taken. Error bars represent 95% Confidence Intervals of the estimate. Effect sizes were considered significant (marked with *) if they were below the significance threshold (q = < 0.05).

BD patients not taking any psychotropic medications at the time of scan displayed significantly larger ventricular and putamen volumes, and smaller ICV compared to CN. BD patients taking one, two, or three classes of psychotropic medication had significantly smaller hippocampus and thalamus volumes, and larger ventricles compared to CN, with patients taking two medication classes displaying the largest effect sizes. Additionally, BD patients taking two medication classes had significantly smaller amygdala volumes compared to CN (See Fig. 1, Table 2, Supplementary Table 9). After balancing confounders with propensity scoring in a subsample of participants for whom this detailed clinical information was available (Supplementary Figure 2), patients taking two or more medications displayed significantly lower thalamus and hippocampal volumes than those taking none or one medication, as well as increased ventricular volume (Supplementary Table 10).

**Table 2:** Associations between medication status and subcortical volume when comparing BD to CN.

	Ventricles			Hippocampus			Thalamus			Caudate			Putamen			Globus Pallidus			Amygdala			Accumbens			ICV		
	*d*	*d 95% CI*	q-value	*d*	*d 95% CI*	q-value	*d*	*d 95% CI*	q-value	*d*	*d 95% CI*	q-value	*d*	*d 95% CI*	q-value	*d*	*d 95% CI*	q-value	*d*	*d 95% CI*	q-value	*d*	*d 95% CI*	q-value	*d*	*d 95% CI*	q-value
***Medication Load***																											
Not Taking	0.07	[.02, .12]	**0.037**	-0.01	[-.06, .04]	0.52	-0.01	[-.06, .04]	0.54	0.04	[-.01, .09]	0.17	0.06	[.01, .11]	**0.041**	-0.03	[-.08, .02]	0.29	0.01	[-.04, .06]	0.51	0.02	[-.03, .07]	0.44	-0.06	[-.11, -.01]	**0.045**
Taking one	0.11	[.06, .16]	**0.0003**	-0.08	[-.12, -.03]	**0.015**	-0.06	[-.11, -.01]	**0.041**	0.00	[-.05, .05]	0.58	0.00	[-.05, .05]	0.58	0.00	[-.05, .05]	0.58	-0.02	[-.07, .03]	0.44	0.00	[-.05, .05]	0.57	-0.02	[-.07, .02]	0.34
Taking two	0.19	[.13, .24]	**<0.0001**	-0.11	[-.16, -.06]	**<0.0001**	-0.10	[-.15, -.05]	**0.0007**	-0.01	[-.05, .04]	0.55	-0.01	[-.06, .04]	0.55	-0.02	[-.07, .03]	0.43	-0.07	[-.12, -.02]	**0.030**	-0.04	[-.09, .01]	0.17	-0.03	[-.08, .02]	0.26
Taking three or more	0.15	[.09, .20]	**<0.0001**	-0.08	[-.13, -.03]	**0.0048**	-0.06	[-.11, -.01]	**0.042**	0.02	[-.03, .07]	0.44	0.00	[-.05, .05]	0.44	0.00	[-.05, .05]	0.59	-0.03	[-.08, .02]	0.32	-0.05	[-.10, .00]	0.13	-0.01	[-.06, .04]	0.53
***Traditional-medication classification***																											
Lithium	0.08	[.02, .13]	**0.020**	0.03	[-.02, .08]	0.28	0.06	[.01, .11]	**0.048**	-0.01	[-.06, .04]	0.49	0.03	[-.02, .08]	0.30	-0.04	[-.09, .01]	0.17	0.01	[-.04, .06]	0.51	-0.02	[-.06, .03]	0.46	-0.03	[-.08, .02]	0.32
Antiepileptics	0.18	[.13, .24]	**<0.0001**	-0.09	[-.14, -.04]	**0.0029**	-0.14	[-.19, -.09]	**<0.0001**	-0.03	[-.08, .02]	0.26	-0.07	[-.12, -.02]	**0.035**	-0.03	[-.08, .02]	0.29	-0.04	[-.09, .01]	0.17	-0.05	[-.10, .00]	0.12	-0.04	[-.09, .01]	0.17
Antipsychotics	0.12	[.06, .17]	**0.0002**	-0.09	[-.14, -.04]	**0.0039**	-0.07	[-.12, -.02]	**0.021**	0.06	[.01, .11]	**0.049**	0.05	[.00, .10]	0.10	0.02	[-.03, .07]	0.37	-0.03	[-.08, .02]	0.32	0.00	[-.05, .05]	0.59	-0.03	[-.08, .02]	0.29
Antidepressants	0.05	[-.01, .10]	0.17	-0.06	[-.11, -.01]	**0.041**	-0.05	[-.10, .00]	0.134	0.02	[-.03, .07]	0.45	0.03	[-.02, .08]	0.27	-0.02	[-.07, .03]	0.40	-0.03	[-.08, .02]	0.26	0.01	[-.04, .06]	0.52	-0.03	[-.08, .02]	0.28
***NbN-based medication classification***																											
Valproate	0.22	[.16, .27]	**<0.0001**	-0.10	[-.16, -.05]	**0.0018**	-0.18	[-.23, -.12]	**<0.0001**	-0.03	[-.09, .02]	0.28	-0.09	[-.15, -.04]	**0.0048**	-0.02	[-.08, .03]	0.42	-0.04	[-.10, .01]	0.22	-0.03	[-.09, .02]	0.29	-0.05	[-.11, .00]	0.13
GSCCB	0.06	[.01, .12]	0.085	-0.05	[-.11, .00]	0.13	-0.03	[-.08, .02]	0.32	-0.01	[-.07, .04]	0.49	-0.06	[-.11, .00]	0.10	-0.01	[-.06, .04]	0.53	0.00	[-.05, .06]	0.57	-0.02	[-.07, .04]	0.47	-0.02	[-.08, .03]	0.39
GABA PAM	0.11	[.05, .16]	**0.0029**	-0.06	[-.11, .00]	0.11	-0.05	[-.11, .00]	0.13	0.06	[.01, .12]	0.06	-0.01	[-.07, .04]	0.51	0.04	[-.01, .09]	0.24	0.00	[-.06, .05]	0.59	0.01	[-.05, .06]	0.55	-0.01	[-.07, .04]	0.48
Primarily dopamine antagonists	0.03	[-.02, .09]	0.30	0.02	[-.03, .08]	0.38	0.03	[-.02, .09]	0.28	0.05	[.00, .10]	0.13	0.03	[-.03, .08]	0.35	-0.01	[-.07, .04]	0.48	0.03	[-.03, .08]	0.36	0.02	[-.03, .08]	0.41	0.01	[-.05, .06]	0.53
Dopamine and other antagonists	0.08	[.02, .13]	**0.041**	-0.09	[-.14, -.03]	**0.009**	-0.05	[-.10, .01]	0.171	0.04	[-.02, .09]	0.26	0.02	[-.04, .07]	0.46	-0.01	[-.06, .05]	0.54	-0.05	[-.10, .01]	0.17	0.01	[-.05, .06]	0.55	-0.06	[-.11, .00]	0.10
Dopamine partial agonists	0.00	[-.06, .06]	0.58	0.01	[-.04, .06]	0.51	-0.01	[-.06, .05]	0.55	0.09	[.03, .14]	**0.008**	0.08	[.02, .13]	**0.027**	0.07	[.01, .12]	**0.041**	0.03	[-.02, .08]	0.31	0.06	[.00, .11]	0.10	-0.04	[-.09, .02]	0.26
Targeting Serotonin	0.04	[-.02, .10]	0.26	-0.03	[-.09, .02]	0.29	-0.02	[-.07, .04]	0.46	0.04	[-.01, .09]	0.24	-0.01	[-.06, .05]	0.54	0.01	[-.05, .06]	0.54	-0.04	[-.09, .02]	0.26	0.01	[-.05, .06]	0.53	-0.10	[-.15, -.04]	**0.0034**
Targeting Serotonin and other	0.04	[-.02, .10]	0.28	-0.04	[-.09, .02]	0.26	-0.04	[-.09, .02]	0.25	0.05	[-.01, .10]	0.17	0.01	[-.05, .06]	0.53	0.02	[-.04, .07]	0.44	-0.01	[-.07, .04]	0.48	0.05	[.00, .11]	0.12	0.02	[-.04, .07]	0.44

*d* = Cohen’s *d*, 95% *C*I=95% confidence intervals for cohen’s *d*, *NbN* neuroscience-based nomenclature, q = FDR adjusted p-values, *GSCCB* glutamate sodium/calcium channel blockers, *GABA PAM*=GABA positive allosteric modulators. q-values highlighted in bold indicate statistical significance <0.05.

### Medications classified by syndrome-based medication categories

**Fig. 2:**
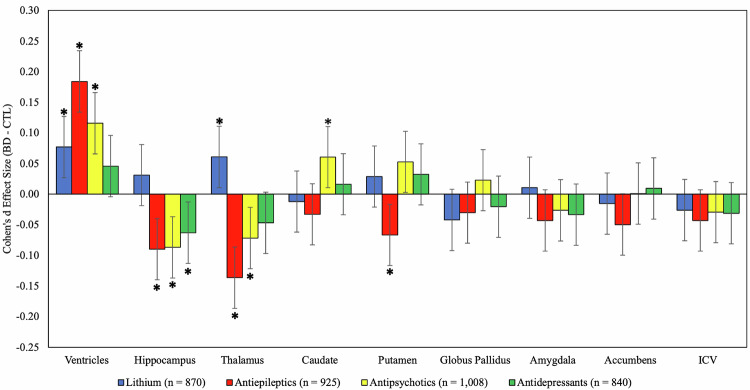
Adjusted effect size estimates for BD patients taking each syndrome-based psychotropic medication class compared to CN. For all analyses including patients taking lithium, antiepileptics, antidepressants and antipsychotics, effect sizes are reported after accounting for age, sex, intracranial volume, and other medications taken. Error bars represent 95% confidence intervals of the estimate. Effect sizes were considered significant (marked with *) if they were below the significance threshold (q = < 0.05).

Figure 2 shows effect size estimates for patients with BD taking each traditional syndrome-based medication category, compared with CN, after accounting for age, sex, ICV and other psychotropic medications taken at the time of scan (see also Supplementary Tables 11-18). Patients taking lithium at the time of scan (Li + ) had significantly larger ventricular and thalamic volumes compared to CN which survived FDR correction. Serum lithium levels were available on a small subset of patients and an ad-hoc analysis did not find any association between Li+ serum levels and subcortical volume (Supplementary Table 19). AED+ patients had significantly smaller hippocampus, thalamus, and putamen volumes, as well as larger ventricular volume compared with CN. Similarly, AP+ patients had significantly smaller hippocampus and thalamus volumes as well larger caudate volumes (bilaterally and in the right hemisphere), along with larger ventricular volumes. AD+ patients had significantly smaller hippocampus volume when compared with CN.

When directly comparing patients on/off different medications, Li+ patients had significantly larger thalamic and hippocampal volumes compared to Li- at the time of scan. AED+ patients had significantly smaller hippocampus, thalamus, caudate, and putamen volumes and larger ventricles compared to AED- patients. AP+ patients had significantly smaller thalamic volumes compared to AP- patients (Supplementary Figures 3-6 and Supplementary Tables 20-23).

### Medications classified by NbN mechanism of action

**Fig. 3:**
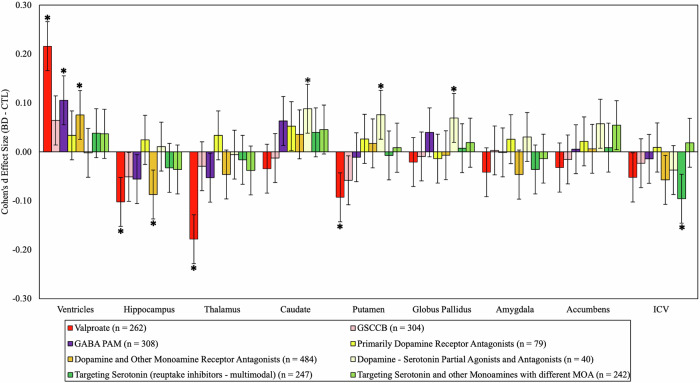
Subcortical differences between patients taking NbN classified psychotropic medication compared to healthy controls and controlling for psychotropic medication usage. Cohen’s d estimates of subcortical volumetric differences between patients taking NbN classified psychotropics and healthy controls while controlling for age, sex, ICV (for subcortical volumes), and psychotropic medication. Error bars show 95% confidence intervals. Effect sizes were considered significant (marked with *) if they were below the significance threshold (q = <0.05). GSCCB = Glutamate sodium/calcium channel blockers, GABA PAM = GABA positive allosteric modulators, MOA = Mechanism of Action.

When modelling AED associations by mechanism of action, patients taking valproate displayed a widespread pattern of smaller hippocampus, thalamus, and putamen volumes, and larger ventricular volume compared with CN. In contrast, patients taking glutamate, sodium/calcium channel-blockers (e.g., lamotrigine) showed no significant subcortical volume differences compared with CN. Patients taking GABA-positive allosteric modulators (e.g., benzodiazepines) showed greater ventricular volume compared with CN. In relation to AP, there were no significant differences in subcortical volumes between patients taking primarily dopamine receptor antagonists and CN. However, patients taking dopamine and other monoamine receptor antagonists showed smaller hippocampal and larger ventricles compared with CN. Furthermore, BD patients taking dopamine-serotonin partial agonists and antagonists showed larger volume in the globus pallidus, caudate and putamen. BD patients taking medications primarily targeting serotonin, as well as those targeting serotonin and other monoamines did not differ significantly from CN. However, patients taking drugs primarily targeting serotonin had significantly smaller ICV compared with CN (Table 2, Fig. 3, and Supplementary Tables 24-31). To ensure comparability of results, demographic and clinical information for those patients on whom NbN medication categorisation was available are also provided in Supplementary Table 32, and sensitivity analyses were conducted by restricting traditional syndrome based medication class analyses to the subset of patients included in the NbN classification. Similar effect sizes were found for the majority of the syndrome based medication categories described above, although the association between AP+ and smaller thalamic volume reduced and was not significant in this smaller sample size (Supplementary Table 33).

### Concurrent medication interactions

For the moderation analysis of concurrent medication administration, co-administration of Li significantly weakened the association of AED with hippocampal volume (β = 0.19, q = 0.038), explaining 31% of the variance (Supplementary Figure 6; Supplementary Table 34). No other significant moderation effects of Li on medication status were observed on subcortical volumes.

### Moderating effect of medication class on associations between illness course indicators and subcortical brain volumes

Associations of clinical course indicators with subcortical volumes are shown in Supplementary Table 35 and with medication status in Supplementary Table 36. More episodes of mood exacerbation, longer duration of illness, BD subtype I vs II, and early age of onset were significant, albeit weakly, correlated with smaller subcortical volume in several brain regions and with psychotropic medication status. However, the association between illness course variables and subcortical volumes were not significantly moderated by medication status (Supplementary Tables 37-44). Psychotropic medication use showed significant geographical variation (Supplementary Table 45), with the strongest regional differences observed for Li (higher rates in Europe, lower in North America; Cramér’s V = 0.21), and smaller but significant effects for AED, AP and AED use (Cramér’s V = 0.10–0.14).

## Discussion

In the largest mega-analytic study of standardized subcortical brain measures conducted to date, using syndrome-based and NbN mechanism of action classifications and covarying for concurrent medication status, we identified various differential associations between psychotropic medication treatment and brain volumes in BD patients compared to CN as well as to patients not taking medications. The associations detected were subtle, with most effect sizes detected < 0.2. This study primarily encompassed collection and analyses of detailed medication information, and detailed clinical information was only available on a subset of individuals. In this subset, clinical indicators of illness severity, such as greater number of mood episodes, psychiatric hospitalisations, longer duration of illness and BD I disorder were associated with lower subcortical volumes. However, we did not find evidence of moderating effects of medication status on the associations of illness severity with subcortical volumes, suggesting that the observed medication–volume relationships are not solely explained by underlying illness severity.

Consistent with prior research [3], BD patients, irrespective of medication status, exhibited larger ventricles compared to CN. In patients taking one, two or more psychotropic medications, larger ventricles, and smaller hippocampal and thalamic volumes were found in an apparent stepwise fashion, when compared to CN. These findings may indicate subcortical atrophy in medicated patients or reflect the cumulative impact of chronic illness or long-term medication use. Given the observational nature of the data, these findings are open to indication bias in that patients taking several medications are more likely to have severe illness or treatment resistance, which could be associated with subcortical volume deficit rather than medication use. However, the propensity score matching analysis in a smaller subsample on whom clinical information was available is consistent with polypharmacy being associated with greater volume reductions of the thalamus, hippocampus and larger ventricular volume even when patients were matched for markers of clinical severity.

In medication-free patients at the time of scan, larger putamen and ventricular volumes alongside reduced ICV were observed. Reduced ICV has been reported previously, particularly in first-episode BD [27] and early-onset affective psychosis [28] and is often interpreted as evidence of impaired neurodevelopment. Larger putamen volume in unmedicated BD is less established but has been reported in our prior meta-analysis [2], voxel-based morphometry studies [29], and in individuals at familial or subsyndromal risk [30]. Additionally, adolescent BD patients, who generally have lower exposure to medication, have been observed to have larger putamen volumes compared to age matched CN [31]. It is important to note, however, that medication-free patients are unlikely to be medication-“naive.” Thus, volumetric differences, such as the larger putamen volume, may reflect the effects of previous medication regimens. Also of note is that the medication free group likely comprises those patients with a milder form of illness compared to medicated patients and displayed lower levels of history of psychosis, duration of illness and number of psychiatric hospitalisations (Table 1).

Li+ patients exhibited larger thalamic and ventricular volumes compared to CN. Additionally, hippocampal volumes were significantly larger than those observed in Li- patients. These differences persisted even after adjusting for other medications, including AED, which exhibit contrasting associations with subcortical volumes. Neuroprotective and neurotrophic effects of Li use have been reported in pre-clinical, as well as in cross-sectional and longitudinal neuroimaging research [5, 9, 11, 20, 32]. The potential neuroprotective effects of Li were further supported here in a moderation analysis in which co-administration of Li was found to weaken the association of AED+ with hippocampal volume. Although the precise mechanism remains elusive, Li may facilitate beneficial neural adaptations by inhibiting pro-apoptotic pathways such as glycogen synthase kinase-3β (GSK-3β), enhancing neuroprotective mechanisms like B-cell lymphoma protein-2 (bcl-2), and upregulating neurotrophic signaling through BDNF [5, 32]. Additionally, its effects on purinergic signaling and inflammatory pathways are reported to play a significant role [33].

AED+ patients had significantly smaller hippocampus, thalamus, and putamen volumes, as well as larger ventricular volume compared with CN. Using the NbN classification, associations between AED use and smaller subcortical brain volumes were largely linked to valproate use and did not extend to other AEDs involving ion-channel blocking to modulate the glutamatergic system. This aligns with existing evidence linking valproate use to variations in gray and white matter volume, thinner cortex, and larger ventricular volumes in patients with epilepsy [34, 35]. The precise mechanism through which valproate may be associated with smaller subcortical brain volume remains unclear. Given the literature on adverse neurological and teratogenic effects from valproate exposure in utero, possible neurotoxic associations have been described in preclinical literature, including hyperammonemia-induced excitotoxicity, impaired mitochondrial function, oxidative stress and inflammation [36]. It is also possible that valproate is associated with smaller subcortical brain volume due to reasons unrelated to pharmacological exposure to this medication, such as the clinical characteristics of the patients prescribed this medication.

AP+ patients exhibited significantly smaller hippocampus and thalamus volumes, alongside larger caudate and ventricular volume, compared to CN, while lower thalamic volumes were noted in AP+ versus AP- patients. The association of AP use with lower hippocampal and thalamic volume aligns with findings from preclinical literature and neuroimaging studies of schizophrenia [3, 8, 23, 37]. Regarding NbN classification, the larger subgroup of AP+ patients taking dopamine and other monoamine receptor antagonists displayed smaller hippocampal volumes and larger ventricles, indicating that this class of medication may be responsible for the associations between AP+ (more generally) and lower brain volume. Interestingly, BD patients taking dopamine-serotonin partial agonists and antagonists showed larger basal ganglia volumes, including the globus pallidus, caudate, and putamen. This finding is consistent with several studies that have replicated the association of larger basal ganglia volumes with atypical AP use [38, 39], thought to reflect striatal hypertrophy, potentially serving as a compensatory response to neuroleptic antagonism of dopamine receptors [40]. There were no significant differences in clinical characteristics across the NbN types for AP patients, suggesting that the observed volumetric changes may be related to medication effects rather than variations in illness severity or other clinical factors.

In relation to AD treatment, smaller hippocampal volumes were observed in AD+ patients compared to CN. When using the NbN classification, AD+ patients on medications primarily targeting serotonin- such as multimodal reuptake inhibitors- showed smaller intracranial volumes (ICV) compared to CN. Given that smaller ICV may suggest a disrupted neurodevelopmental trajectory, this association is unlikely to be attributable to medication effects [27]. The observed reduction in hippocampal volume is consistent with the broader literature linking depressive illness to hippocampal shrinkage [41], as AD+ patients typically had more depressive episodes and higher ratings on depressive symptom scales compared to other subgroups (Table 1). However, medication class did not significantly moderate the effects of clinical course measures on subcortical volumes.

Anxiolytics and hypnotics (including benzodiazepines) were recoded into the NbN category GABA positive allosteric modulators which were associated with larger ventricles compared to CN. Greater ventricular volumes in patients taking benzodiazepines, as well as lower hippocampal and striatal volumes, have been reported in patients with anxiety disorders [42]. The observed ventricular enlargement could be a direct medication effect or indirectly related to anxiety associations (e.g., substance use, chronic sleep problems).

### Strengths and limitations

The major strengths of this study include its large sample size, standardized data processing and analysis, and the use of pooled individual-level data to account for relevant covariates and co-prescribed medications. This extensive dataset allowed us to investigate interactions among variables that smaller, less powered studies could not conclusively address. Notably, this is the first mega-analytic study to employ both syndrome and mechanism-of-action-based medication classification approaches to assess medication associations with subcortical volume in BD. Although our findings align with prior evidence suggesting potentially neuroprotective effects of Li in comparison to valproate, which has been linked to the opposite pattern, these results should be interpreted cautiously given the cross-sectional study design which precludes our ability to establish causal relationships between psychotropic medication status and morphometric changes or support clinical recommendations. Instead, they highlight the importance of targeted randomized controlled trials incorporating neuroimaging and functional outcomes to clarify the relative effects of mood-stabilizing medications in BD.

Further, the observed associations could be influenced by a variety of confounding factors which were under-explored in this study, including but not limited to medication adherence, the severity of illness, duration of treatment, and other clinical characteristics. The direction of causality could be reversed in that morphometric changes could pre-exist and be responsible for the subsequent medication selection. Strategies to tackle indication bias with opportunities for mitigation such as longitudinal imaging studies integrated into randomized trials are necessary to explore causal relationships and changes in brain structure over time. Such studies are very challenging to conduct, but some previous research has shown that AP medication treatment is associated with increased pallidal volume over time in first episode psychosis [43], and that Li treatment can slow white matter volume reduction in the internal capsule in BD [44]. The ENIGMA-BD consortium conducted the largest longitudinal observational study to date and did not find any association between the annual rate of brain structure change and classic ‘syndrome-based’ medication classes [45]. However, this study only included approximately 300 bipolar disorder patients and had a limited scan interval, underscoring the need for more extensive longitudinal studies. Given that structural brain imaging changes may not necessarily translate to functional outcomes, future studies on medication associations would benefit from including functional imaging and functional outcome measures such as neurocognitive performance, social and occupational functioning. Machine learning and artificial intelligence driven multimodal approaches could further assist to address causal direction and refine stratification of illness, medication and outcome variables, in particular as larger and richer datasets become more accessible through further collaborative data sharing initiatives.

While the use of the NbN approach represents a significant advancement in medication classification by accounting for the pharmacological mechanisms of action, it is important to recognize that this method, though more refined, still has limitations. The categorization of medications based on presumed mechanisms of clinical action assumes that these mechanisms are fully understood- a premise that is not always accurate. In addition, we faced limitations in including all relevant clinical variables in our main analyses. Our primary goal was to delineate how medication status correlates with subcortical volumes, but the complexity of including numerous clinical covariates was constrained by sample size. Variability in individual studies regarding inclusion criteria, diagnostic and clinical data collection, and medication details also affected the granularity of our analyses. For example, NbN analyses could only be conducted for approximately 48% of patients with available medication data. Although the NbN findings largely align with and develop the syndrome based findings, one discrepancy of note is that the association of thalamic volume reduction with antipsychotic medication was not significant for any NbN subcategory of dopamine modulating medications. However, as with hippocampal volume reduction, the ‘Dopamine and other monoamine receptor antagonists’ subcategory displayed a similar direction of effect with a smaller effect size than the syndrome based classification (-0.05 vs -0.07), and the lack of statistical significance may be related to the smaller sample size analysed.

Additionally, medication status at the time of the scan does not fully reflect historical medication use. Although we had access to medication type and polypharmacy and inferred that most patients included were likely to be taking these prescribed medications for a period of time, we did not have detailed access to dose of medication, duration of medication treatment or past use of different medications, all of which could be associated morphometric changes detected at the time of imaging. Furthermore, a small number of BD patients (251 out of 2,664) had no information available for medication status. Medication effects remain a critical area for future ENIGMA-BD studies and is actively collecting further detailed clinical information, particularly with a focus on incorporating detailed information about medication dosage and history as well as harmonising clinical measures. Given the multicentre international dataset, there are likely regional and cultural variables which could influence prescribing practices. Notably the proportions of patients prescribed the traditional medication classes differ between North America, Europe and Rest of World (Supplementary Table 44), with a greater proportion of the latter group taking antiepileptic and antipsychotic medications. The associations of brain morphometric changes with medication class could potentially be confounded by variation in regional environmental exposures and availability of health service provision. Given that the psychotropic medications examined in this study are also commonly used in other psychiatric conditions such as major depressive disorder and schizophrenia, this study highlights the need to carefully model psychotropic use in any cross disorder structural neuroimaging studies. Findings considered to be diagnostic differences could be confounded by differential medication use across disorders, such as lithium in bipolar disorder and antipsychotics in schizophrenia.

## Conclusion

This study presents a detailed analysis of the associations of psychotropic medication status with subcortical brain volume in patients with BD. Our findings reveal that medication-free BD patients have similar subcortical volume to CN aside from putamen and ventricular enlargement, whereas patients on concurrent medications generally exhibit pronounced subcortical variations. AED and AP use were associated with ventricular enlargement and lower hippocampal and thalamic volumes. NbN classification revealed that these associations appeared to be associated with valproate and with dopamine and other monoamine receptor antagonists respectively. Conversely, Li use was associated with larger hippocampal and thalamic volumes than those not taking Li, even when adjusting for concurrent usage of psychotropic medications. Further, concurrent Li use weakened the negative association of AED use with hippocampal volume, reinforcing its potential neuroprotective properties. Whilst subcortical variations were linked to several indicators of clinical severity, these were not modulated by medication class. Longitudinal studies and ideally randomised controlled trials will be necessary to disentangle the potentially complex associations between psychotropic medication status and subcortical volume reductions.

## Supplementary information

Supplemental Material

## Data Availability

Data are made available upon request.

## Code availability

All analyses were conducted in R version 4.1.1. Code is available upon request.
