# Supplementary material for: Psychotropic medications and their interactions with subcortical brain volume in bipolar disorder: An ENIGMA mega-analysis
Source: Mol Psychiatry. 2026 Jan 15;31(5):2941–53. doi: 10.1038/s41380-025-03432-z (PMC13099646; doi:10.1038/s41380-025-03432-z)
Supplement: Supplementary file 1 — Supplemental Material [file 41380_2025_3432_MOESM1_ESM.docx]

**Supplementary Materials**

**Table of Contents**

[Supplementary Note 1. Description of image and volume segmentation quality control 5](#_heading=h.foccppmzevw7)

[Supplementary Note 2. Neuroscience based Nomenclature (NbN) classification 6](#_heading=h.vlnluumjnbbp)

[Supplementary Note 3. Statistical Analysis 7](#_heading=h.tu2tsexjt8ji)

[Supplementary Figure 1. Subcortical volumetric differences between BD patients (n = 2664) and CN (n = 4065) 10](#_heading=h.wcnma2us4as6)

[Supplementary Figure 2. Covariate balance before and after propensity score weighting 11](#_heading=h.apox42xak80g)

[Supplementary Figure 3. Subcortical volumetric differences between patients taking (n = 870) lithium and not taking (n = 1554) lithium 12](#_heading=h.qr9qa6o5hg66)

[Supplementary Figure 4. Subcortical volumetric differences between patients taking (n = 925) and not taking (n = 1489) antiepileptics 13](#_heading=h.ix7fjc8c1gva)

[Supplementary Figure 5. Subcortical volumetric differences between patients taking (n = 1008) and not taking (n = 1415) antipsychotics 14](#_heading=h.uraj1f4x1pvu)

[Supplementary Figure 6. Subcortical volumetric differences between patients taking (n = 840) and not taking (n = 1574) antidepressants 15](#_heading=h.pv52lxnub8mp)

[Supplementary Figure 7. Concurrent lithium use moderates the association between antiepileptic use and lower hippocampal volume 16](#_heading=h.l75loailm4m6)

[Supplementary Table 1. ENIGMA – Bipolar Disorder Working Group Demographics – case-control breakdown for participating sites 17](#_heading=h.x7wh9iuoowfx)

[Supplementary Table 2. Diagnosis and medication information 18](#_heading=h.easmnbdtiwf9)

[Supplementary Table 3. Inclusion and Exclusion criteria for each site 20](#_heading=h.c7dfxgc503z4)

[Supplementary Table 4. Image acquisition and processing details by site 25](#_heading=h.5j73vvoc5bcz)

[Supplementary Table 5a. Pharmacological domains and mechanisms of action of NbN 27](#_heading=h.x9stwpvswx51)

[Supplementary Table 5b. NbN classification of psychotropic medication 28](#_heading=h.2tzoo6mtsjne)

[Supplementary Table 6. Subcortical volumetric differences between patients with bipolar disorder and healthy controls 39](#_heading=h.i8hc60oufkom)

[Supplementary Table 7. Number of Cases where hemispheric volumes were missing 39](#_heading=h.aswtujpls5l8)

[Supplementary Table 8. Subcortical volumetric differences between patients with bipolar disorder and healthy controls across hemispheres 39](#_heading=h.odxwojwtp324)

[Supplementary Table 9. Associations between the number of psychotropic medications BD patients are taking at the time of scan and subcortical volume compared with CN 41](#_heading=h.x00bjjmdcmn)

Supplementary Table 10. Propensity score matching association of number of psychotropic medications BD patients are taking at the time of the scan with subcortical volume………..43

Supplementary Table 11. The association of lithium treatment with subcortical volume – BD patients taking lithium vs. CN………………………………………………………………..44

[Supplementary Table 12. The association of antiepileptic treatment with subcortical volume – BD patients taking antiepileptics vs. CN 44](#_heading=h.3sha806uvjvb)

[Supplementary Table 13. The association of antipsychotic treatment with subcortical volume – BD patients taking antipsychotics vs. CN 44](#_heading=h.rvkf1lrythnx)

[Supplementary Table 14. The association of antidepressant treatment with subcortical volume – BD patients taking antidepressants vs. CN 45](#_heading=h.sxy363acydpg)

[Supplementary Table 15. The association of lithium treatment with subcortical volume – BD patients taking lithium vs. CN across hemispheres 45](#_heading=h.f03vhy5rv15q)

[Supplementary Table 16. The association of antiepileptic treatment with subcortical volume – BD patients taking antiepileptics vs. CN across hemispheres 46](#_heading=h.buq4cp4svy4v)

[Supplementary Table 17. The association of antipsychotic treatment with subcortical volume – BD patients taking antipsychotics vs. CN across hemispheres 47](#_heading=h.uws02bi4b4mg)

[Supplementary Table 18. The association of antidepressant treatment with subcortical volume – BD patients taking antidepressants vs. CN across hemispheres 48](#_heading=h.tccdcqf06of2)

[Supplementary Table 19. The association between lithium serum levels and subcortical volume in BD 48](#_heading=h.1mn89xxr3h4t)

[Supplementary Table 20. The association of lithium treatment with subcortical volume – BD patients taking lithium vs. BD patients not taking lithium 49](#_heading=h.er0wv1pfi78t)

[Supplementary Table 21. The association of antiepileptic treatment with subcortical volume – BD patients taking antiepileptics vs. BD patients not taking antiepileptics 49](#_heading=h.rbq31s7x65jm)

[Supplementary Table 22. The association of antipsychotic treatment with subcortical volume – BD patients taking antipsychotics vs. BD patients not taking antipsychotics 50](#_heading=h.jdfprulskn2a)

[Supplementary Table 23. The association of antidepressant treatment with subcortical volume – BD patients taking antidepressants vs. BD patients not taking antidepressants 50](#_heading=h.snexxmysdt5w)

[Supplementary Table 24. The association of valproate treatment with subcortical volume – BD patients taking valproate vs. CN 51](#_heading=h.34bwxycl01l5)

[Supplementary Table 25. The association of glutamate sodium/calcium channel-blockers (GSCCB) with subcortical volume – BD patients taking glutamate sodium calcium channel-blockers vs. CN 51](#_heading=h.u48fk0eo5fb3)

[Supplementary Table 26. The association of GABA positive allosteric modulators (GABA PAM) with subcortical volume – BD patients taking GABA positive allosteric modulators vs. CN 52](#_heading=h.qhb986iy1uif)

[Supplementary Table 27. The association of primarily dopamine receptor antagonists with subcortical volume – BD patients taking primarily dopamine antagonists vs. CN 52](#_heading=h.h1d5x9m0eihu)

[Supplementary Table 28. The association of dopamine and other monoamine receptor antagonists with subcortical volume – BD patients taking dopamine and other receptor antagonists vs. CN 53](#_heading=h.70n5hhscgvuj)

[Supplementary Table 29. The association of dopamine-serotonin partial agonists and antagonists with subcortical volume – BD patients taking dopamine partial agonists vs. CN 53](#_heading=h.yrdjav6bei6f)

[Supplementary Table 30. The association of drugs targeting serotonin with subcortical volume – BD patients taking drugs targeting serotonin vs. CN 54](#_heading=h.bvrle4tgk7nb)

[Supplementary Table 31. The association of drugs targeting serotonin and other monoamines with subcortical volume – BD patients taking drugs targeting serotonin and other vs. CN 54](#_heading=h.2bckoc8s2xy2)

[Table 32. Demographic and Clinical Comparisons in NbN subset sample 55](#_heading=h.vjudw1zbgm8z)

[Supplementary Table 33. The association of traditional syndrome-based medication with subcortical volume – BD patients taking vs. CN of subset sample included in NbN analysis 57](#_heading=h.ktr7y9clui4a)

[Supplementary Table 34. The moderating role of lithium on the associations between antiepileptics and antipsychotics with subcortical volume in BD patients 58](#_heading=h.pzss9gzclehp)

[Supplementary Table 35. Correlation between illness course measures and subcortical volume in BD patients 59](#_heading=h.gshuvy43803o)

[Supplementary Table 36. Group differences between illness course measures, BD subtype, BMI and psychotropic medication use in BD patients 60](#_heading=h.4uhcl4g2hn0v)

[Supplementary Table 37. The moderating role of psychotropic drugs on the relationship between total manic episodes and subcortical volume in BD patients 61](#_heading=h.7bbz9v40oxek)

[Supplementary Table 38. The moderating role of psychotropic drugs on the relationship between total hypomanic episodes and subcortical volume in BD patients 61](#_heading=h.b4etndsnvm15)

[Supplementary Table 39. The moderating role of psychotropic drugs on the relationship between total depressive episodes and subcortical volume in BD patients 61](#_heading=h.goys6h562ni6)

[Supplementary Table 40. The moderating role of psychotropic drugs on the relationship between total psychiatric hospitalizations and subcortical volume in BD patients 62](#_heading=h.mly9p4dxyrh9)

[Supplementary Table 41. The moderating role of psychotropic drugs on the relationship between a history of psychosis and subcortical volume in BD patients 62](#_heading=h.lzfg88rr3its)

[Supplementary Table 42. The moderating role of psychotropic drugs on the relationship between age of onset and subcortical volume in BD patients 62](#_heading=h.1t6imu63suj)

[Supplementary Table 43. The moderating role of psychotropic drugs on the relationship between illness duration and subcortical volume in BD patients 63](#_heading=h.32128tono0u1)

[Supplementary Table 44. The moderating role of psychotropic drugs on the relationship between BD subtype I vs II and subcortical volume in BD patients 63](#_heading=h.aess57dxsv8d)

[Supplementary Table 45. Regional variation in lithium, antiepileptic, antipsychotic, and antidepressant use among patients with bipolar disorder 64](#_heading=h.vg1n5qwdykte)

# Supplementary Note 1. Description of image and volume segmentation quality control

Scanning was performed on several different platforms and no specific harmonization in acquisition was obtained to reduce geometric distortion effects (using a phantom for calibration). All analyses and quality checks were done without knowing the subjects' diagnoses. A validated automated segmentation tool, FreeSurfer, was used to analyze the images. FreeSurfer was used to derive measures for the lateral ventricles, hippocampus, thalamus, caudate, putamen, globus pallidus, amygdala, nucleus accumbens, and ICV. Each site visually inspected each image for segmentation errors. Additionally, volumes more than 3 standard deviations away from the site's average for each structure were flagged and re-examined visually. Segmentation outliers that were deemed correct upon re-inspection were retained in the analysis. In total, 12.5% of lateral ventricular volumes, 1.8% of amygdala volumes, 1.3% of nucleus accumbens volume, 1.8% of hippocampal volumes, 1.3 % of thalamic volumes, 1.2% of putamen volumes, 1.5% of globus pallidus volumes, 1.4% of caudate volumes, and 0.7% of ICV failed quality control and were removed from analyses.

# Supplementary Note 2. Neuroscience based Nomenclature (NbN) classification

The traditional system used for the classification of psychotropic medication is based on clinical indication and groups pharmacological agents into broad categories such as “mood stabilizers”, “antipsychotics”, and “antidepressants”. However, clinical indication-based nomenclature fails to effectively capture the range of pharmacological domains on which these drugs act and the mechanisms of action through which they exert their effects, and it is likely that these are more relevant when it comes to assessing neurobiological associations of psychotropic medication use.

To address the need for an evidence-based and pharmacologically driven classification system, the European College of Neuropsychopharmacology set up a “Nomenclature Taskforce” which culminated in the first edition of the “Neuroscience-based Nomenclature” (NbN). Under the multi-axial NbN classification system, psychotropic drugs are categorized according to their pharmacological domain, mode of action, approved indication, efficacy, side effects, and practical notes. A search engine for NbN classified psychotropic medications is available on the NbN website (available here: <https://nbn2r.com/>). As the NbN is still in development and the mechanism of action of a number of psychotropic compounds remains unclear, several psychotropic drugs have not yet been fully classified.

For the present study, information regarding medication use at the time of scan (i.e., name of medication or Anatomical Therapeutic Chemical (ATC) code) was collected across the 34 sites and recoded using the NbN classification system. Such detailed information on medication name was available on a subset of our sample (48% of BD patients) and thus the NbN analyses were confined to this cohort. We opted to recode medication based on the pharmacological domain and mode of action. The pharmacological domain represents the neurotransmitter, molecule, or system in which the psychotropic drug modulates. The mode of action details the effect a psychotropic drug has on its pharmacological target. (See Supplementary Table 5a) It is important to note that a psychotropic drug can act on multiple pharmacological domains via multiple modes of action, and this is reflected in the NbN classification system. Firstly, we listed all psychotropic medications taken by patients at the time of scan in which we were provided their medication name or their ATC code. Secondly, we cross-checked the medication names and ATC codes with the NbN system, identifying the pharmacological domains and modes of action for each drug. Medications not listed in the NbN classification and those taken by less than 10 patients were added to the “Other Psychotropics” category (See Supplementary Table 5b). Moreover, the pharmacological domain and mode of action of some psychotropic medications is still poorly understood and not yet specified in the NbN system. Notable examples of this relevant to the current study include lithium, valproate, and topiramate. Due to the common role of lithium and valproate in the management of bipolar disorder, we decided to assign these to their own separate categories. Topiramate was added to the “Other Psychotropics” category.

Due to the potentially large number of combinations between pharmacological domain and mode of action, we condensed the variables based on pharmacological similarity into a final 10 NbN based categories (See Supplementary Table 5c). Antipsychotics were recoded into three different NbN categories (1) Primarily dopamine receptor antagonists, (2) Dopamine and other (serotonin-norepinephrine) monoamine receptor antagonists, and (3) Dopamine, serotonin receptor partial agonist/antagonists. Antidepressants were recoded into two NbN categories (4) Targeting primarily serotonin (reuptake inhibitors multimodal) and (5) Targeting serotonin and other monoamines with different mechanisms of action. Antiepileptics were recoded into (6) glutamate sodium/calcium channel blockers and (7) Valproate. Lithium (8) was assigned its own NbN category. Anxiolytics and hypnotics were recoded into the category GABA positive allosteric modulators (9). “Other Psychotropics” (10) category is composed of drugs not included in the NbN system, whereby the pharmacological domain or mode of action is unclear, or when there were less than 10 patients taking a given medication at the time of scan. We excluded the ‘Other Psychotropics’ category from the statistical analysis due to the heterogeneity of drugs in this category in terms of clinical indication, pharmacological domain, and mode of action. Moreover, since lithium was included in our analysis of psychotropic medication classified using a clinical indication-based approach, this medication was not reanalyzed but rather included as a covariate in our NbN analyses of the other 8 categories.

# Supplementary Note 3. Statistical Analysis

**Cohort Characteristics**

Differences in demographic and clinical variables (See in Table 1) between bipolar disorder (BD) patients and healthy controls (CN), and BD patients not taking any medication, taking one class, two classes, and three or more classes were tested using t tests, chi-squared tests, ANOVA, and Kruskal-Wallis test. Pearsons r and Spearman’s g were used to measure the correlation between illness course indicators and subcortical volume in BD patients.

**Linear Mixed Effect Regression Analyses**

Linear mixed-effect regression (lmer) modelling was used to investigate associations between psychotropic medication use at the time of scan with subcortical volume, lateral ventricle volume, and ICV in BD patients compared to healthy controls. All analyses were conducted using the lme4 package in R. In each model, we included the subcortical region of interest as the dependent variable. We covaried for age, sex, and ICV (unless ICV was the dependent variable) by including them as fixed effects and included “Site” as a random effect to control for methodological heterogeneity such as differences the field strength of MRI scanners, imaging sequences, and image processing. In total, we ran 144 lmer models.

We examined differences between BD patients and CN while not controlling for medication to establish baseline differences. We ran nine lmer models (one for each subcortical region of interest) including “Group” as a fixed effect (coded as “0” = CN, “1” = BD).

Example of syntax: lmer(BilateralCaud ~ Group + ICV + Age + Sex + (1 | Site), data = Data)

We analyzed differences between BD patients not taking any classes of psychotropic medication, taking one class, taking two classes, and taking three classes compared to CN. Psychotropic medication included in this analysis was classified using a clinical indication-based approach (i.e. lithium, antipsychotics, antiepileptics, and antidepressants). The “MedicationLoad” variable was created by adding the number of clinical indication-based classes BD that patients were taking at the time of scan (coded as “0” = CN, “1” = Not Taking, “2” = Taking One Class, “3” = Taking Two Classes, “4” = Taking Three or More Classes). We did not control for the effects of specific medications in this analysis. We ran one lmer model per subcortical region of interest, totaling nine lmer models.

Example of syntax: lmer(BilateralAmyg ~ MedicationLoad + ICV + Age + Sex + (1 | Site), data = Data)

We investigated differences between BD patients using psychotropic medication at the time of scan compared to CN while controlling for potential concurrent medication use. Psychotropic medication included in this analysis was classified using a clinical indication-based approach (i.e., lithium, antipsychotics, antiepileptics, antidepressants). To compare BD patients taking medication to CN, the CN group was set as the reference (coded as 0). However, as the CN group was replicated across each medication variable and in order to avoid multicollinearity, we analyzed one “Predictor” variable (coded as “0” = CN, 1 = “Taking”, “2” = Not Taking) per analysis and controlled for potential concurrent psychotropic medication use by including the other medications as “Covariate” variables (coded as “1” = Taking, “2” = Not Taking + CN). This statistical approach allowed us to avoid perfect multicollinearity, control for concurrent psychotropic medication use, and isolate the differences in subcortical volume between BD patients taking a class of psychotropic medication compared to CN. This approach was computationally intensive and resulted in a total of 36 lmer models (4 x 9 with each psychotropic medication as a “Predictor” for each subcortical region of interest).

Example of syntax: lmer(BilateralHippo ~ AntiEpilepticPredictor + LithiumCovariate + AntiDepCovariate + AntiPsychCovariate + Sex + ICV + Age + (1 | Site), data = Data)

We then examined differences between BD patients taking and not taking psychotropic medication at the time of scan while controlling for concurrent medication use. Psychotropic medication included in this analysis was classified using a clinical indication-based approach (i.e., lithium, antipsychotics, antiepileptics, antidepressants). We included each class of psychotropic medication as fixed effects (coded as “0” = Not Taking, “1” = Taking) in a joint analysis. We ran one lmer model per subcortical region of interest, totaling in nine lmer models.

Example of syntax: lmer(BilateralThal ~ LithiumTakingvsNotTaking + AntiEpilepticTakingvsNotTaking + AntiDepTakingvsNotTaking + AntiPsychTakingvsNotTaking + ICV + Age + Sex + (1 | Site), data = Data)

We examined differences between BD patients taking NbN classified psychotropic medication compared to CN (See Supplementary Note 2 and Supplementary Tables 5a-c for more details on the NbN classification system and how it was adopted by the present study) while controlling for potential concurrent medication use. Adopting the NbN classification system allowed for a more nuanced analysis of the association between psychotropic medication use at the time of scan and subcortical volume. Following the same approach as with the our analysis of BD patients taking clinical indication-based classified psychotropic drugs compared to CN, we used a “Predictor” variable (coded as “0” = CN, 1 = “Taking”, “2” = Not Taking) to isolate differences in subcortical volume between BD patients taking the main psychotropic variable of interest and CN while controlling for concurrent medication use by including other NbN classified psychotropic drugs as “Covariates” (coded as “1” = Taking, “2” = Not Taking + CN). We had a total of 8 NbN classified psychotropic medication classes, resulting in a total of 72 lmer models (8 x 9 with each psychotropic medication as a “Predictor” for each subcortical region of interest).

Example of syntax: lmer(BilateralVent_log10 ~ ValproatePredictor + LithiumCovariate + GlutamateChannelBlockersCovariate + TargetingSerotoninCovariate + TargetingSerotoninAndOtherCovariate + PrimarilyDopamineCovariate + DopamineAndOtherCovariate + DopaminePartialAgonistCovariate + OtherCovariate + BenzosCovariate + ICV + Age + Sex + (1 | Site), data = Data)

**Moderation Analyses**

Moderation analyses were used to further investigate whether psychotropic medication use at the time of scan changes the strength or direction of the association between other psychotropic medications and illness course indicators with subcortical volume. We used the bruceR package in R as this allows us to account for the hierarchical nature of the dataset by including “Site” as a random effect.

We examined whether concurrent lithium use interacts with the significantly identified associations between antiepileptics and antipsychotics on subcortical volume when comparing BD taking and not taking psychotropic medications at the time of scan. (See Supplementary Tables 12-15 and Supplementary Figures 2-5). In each model, we included “age”, “sex”, and “ICV” as covariates, as well as psychotropic medications that were not the independent variable or moderator as covariates to control for potential concurrent medication use.

Example of syntax: PROCESS(group_bipolar, y = "BilateralAccumb", x = "AntiEpilepticTakingvsNotTaking", mods = c("LithiumTakingvsNotTaking"), covs = c("Age ", "Sex", "ICV", "AntiDepCovariate", "AntiPsychCovariate"), clusters = c("Site"), ci = c("mcmc"), nsim = 5000, seed = 123456, center = FALSE, std = FALSE, digits = 4)

We examined whether psychotropic medication use at the time of scan interacts with previously identified associations between illness course indicators and subcortical volume. To fulfil the requirements for this exploratory analysis, we first established a relationship between the illness course indicators and the subcortical region of interest. Significant findings were included in the moderation analysis. We only included psychotropic medications as moderators if they were associated with the illness course indicator and the subcortical region of interest. We included psychotropic medications that were not the moderator as covariates to control for potential concurrent medication use.

Example of Syntax: PROCESS(group_bipolar, y = "BilateralAccumb", x = "Manic_Episodes_Total", mods = c("AntiepilepticsTakingvsNotTaking"), covs = c("Age", "Sex", "ICV", "AntiDepCovariate", "AntiPsychCovariate", “LithiumCovariate”), clusters = c("Site"), ci = c("mcmc"), nsim = 5000, seed = 123456, center = FALSE, std = FALSE, digits = 4)

We ran a total of 93 moderation analyses.

**Propensity Score Matching**

Propensity scores were estimated for each pairwise comparison of interest using multinomial regression models implemented in the WeightIt R package (Greifer, 2020)^^[[1]](#footnote-1)^^. Predictors included severity of illness-related covariates: duration of illness, history of psychosis, number of episodes of mood exacerbation and psychiatric hospitalizations. Inverse probability of treatment weights (IPTWs) were computed to estimate average treatment effects, stabilized to reduce variance, and trimmed at the 1st and 99th percentiles to improve robustness. Effective sample sizes were reported to evaluate precision. Prior to weighting, all covariates were inspected for implausible values and outliers (>3 SDs from the mean), which were winsorized when necessary. Covariate balance before and after weighting was evaluated using standardized mean differences (cobalt package), with absolute SMDs < .10 considered evidence of adequate balance. The distribution of weights was examined to identify extreme values; stabilized and trimmed weights were used to minimize the influence of such cases.

For each bilateral subcortical region, weighted linear regression models were fitted, adjusting for ICV, age, sex, and fixed effects for site. To account for clustering by site, robust cluster-corrected standard errors (CR2) were estimated using the clubSandwich package (Pustejovsky & Tipton, 2018)^^[[2]](#footnote-2)^^.

Stabilized IPTW was chosen over matching methods (e.g., nearest-neighbor matching in *MatchIt*) because weighting preserves the full analytic sample and maximizes statistical efficiency. This is particularly important in multi-site neuroimaging datasets where subgroup sizes are modest, and overlap may be limited. In addition, approaches that require complete data (e.g., matching with listwise deletion) can be problematic in clinical cohorts, as missingness in illness-course variables may not occur at random. By applying IPTW with trimming, we achieved covariate balance while maintaining representativeness of the broader sample, providing a principled approach to estimate marginal effects of medication burden on brain structure.

# Supplementary Figure 1. Subcortical volumetric differences between BD patients (n = 2664) and CN (n = 4065)


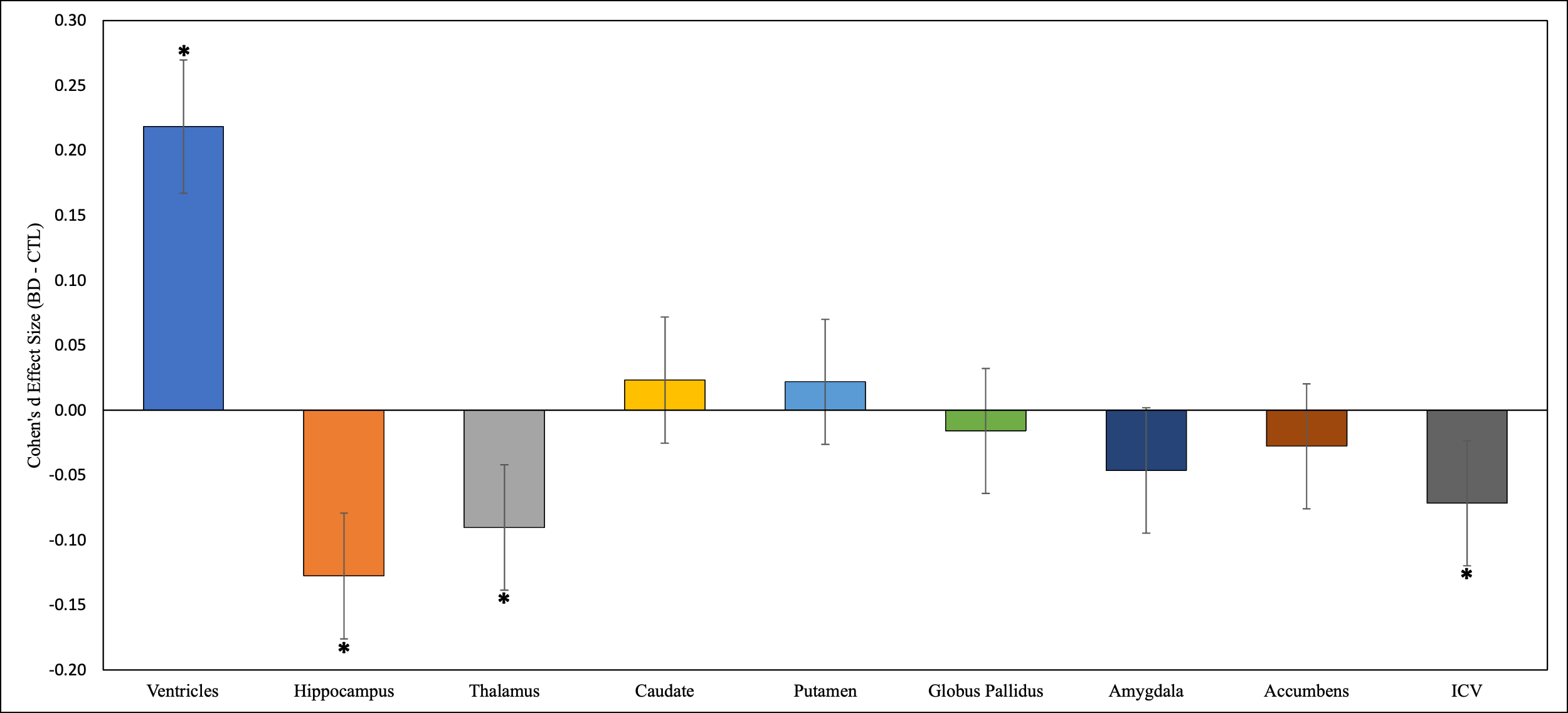


*Note.* Cohen’s d estimates of subcortical volumetric differences between patients with bipolar disorder and healthy controls while controlling for age, sex, and ICV (for subcortical structures). Error bars show 95% confidence intervals. Asterix (*) indicates a q-value of < 0.05.

# Supplementary Figure 2. Covariate balance before and after propensity score weighting


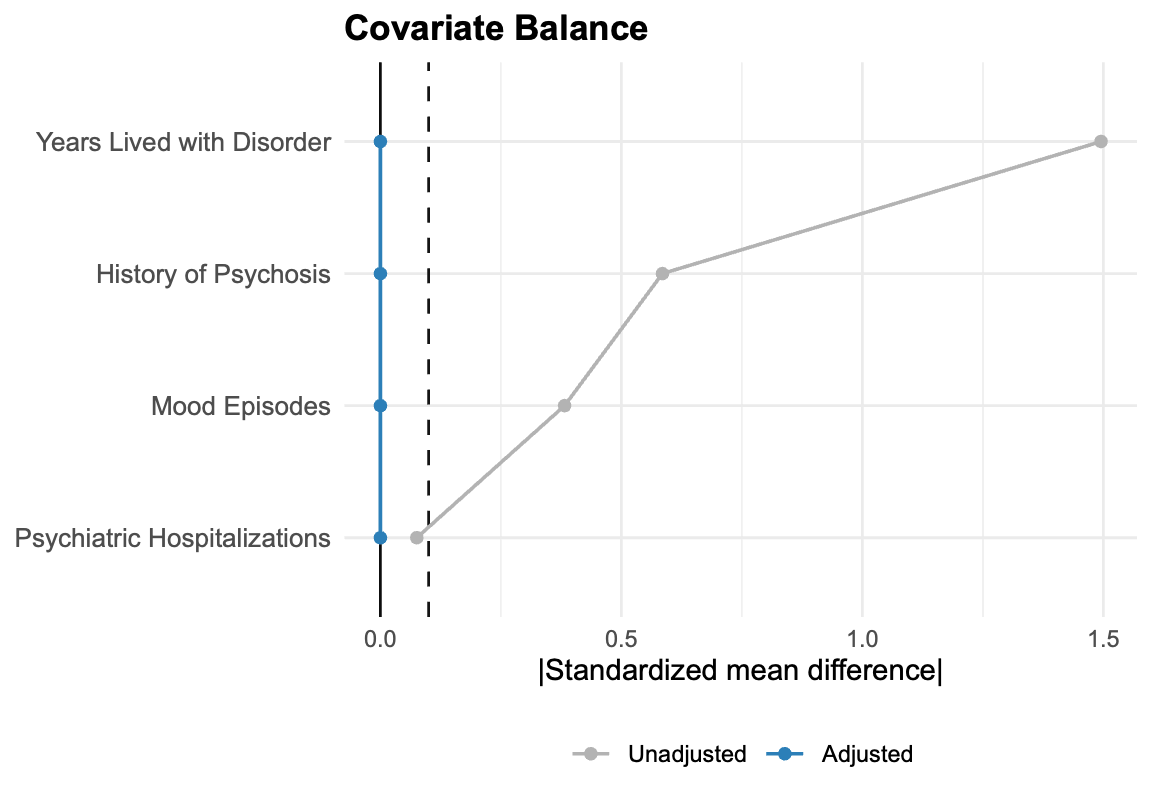


*Note. Standardized mean differences (SMDs) are shown for each covariate. Gray points represent unadjusted values; blue points represent values after weighting. The dashed line indicates the 0.10 threshold for acceptable balance.*

# Supplementary Figure 3. Subcortical volumetric differences between patients taking (n = 870) lithium and not taking (n = 1554) lithium


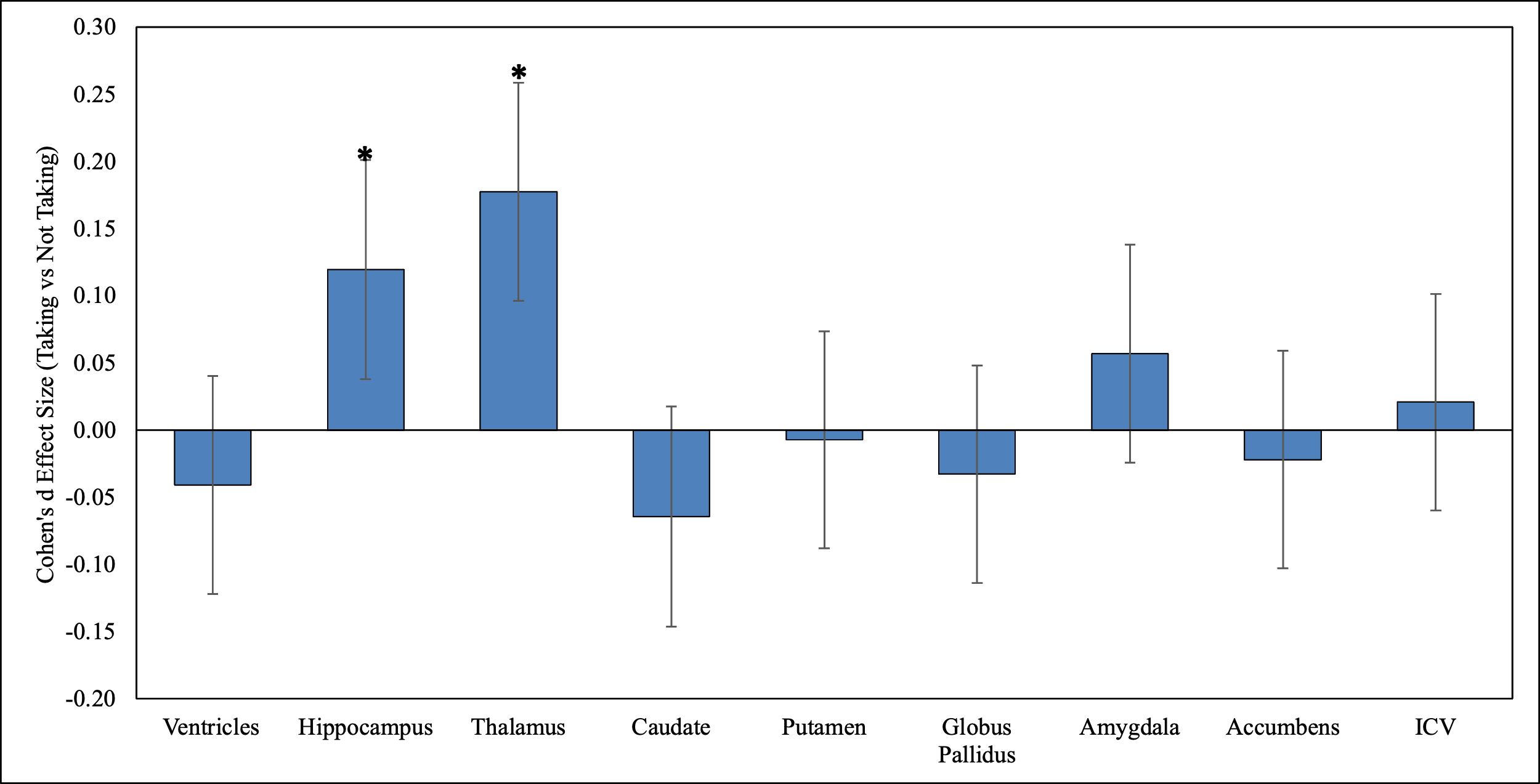


*Note.* Cohen’s d estimates of subcortical volumetric differences comparing patients taking lithium to patients not taking lithium while controlling for age, sex, ICV (for subcortical structures) and psychotropic medication (antiepileptics, antipsychotics, and antidepressants). Error bars show 95% confidence intervals. Black asterisk (*) indicates a q-value of < 0.05.

# Supplementary Figure 4. Subcortical volumetric differences between patients taking (n = 925) and not taking (n = 1489) antiepileptics


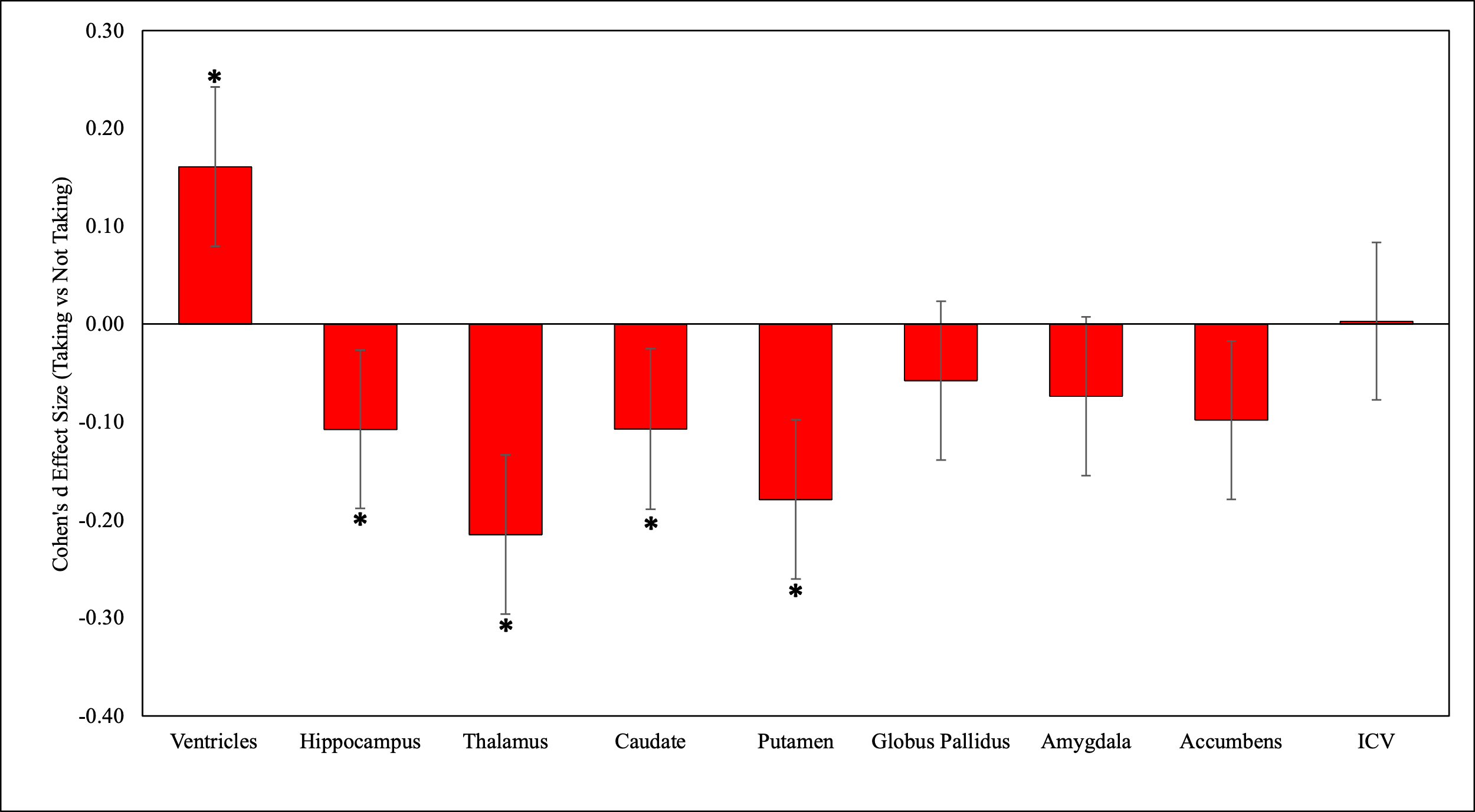


*Note*. Cohen’s d estimates of subcortical volumetric differences comparing patients taking antiepileptics to patients not taking antiepileptics while controlling for age, sex, ICV (for subcortical structures) and psychotropic medication (lithium, antipsychotics, and antidepressants). Error bars show 95% confidence intervals. Black asterisk (*) indicates a q-value of < 0.05.

# Supplementary Figure 5. Subcortical volumetric differences between patients taking (n = 1008) and not taking (n = 1415) antipsychotics


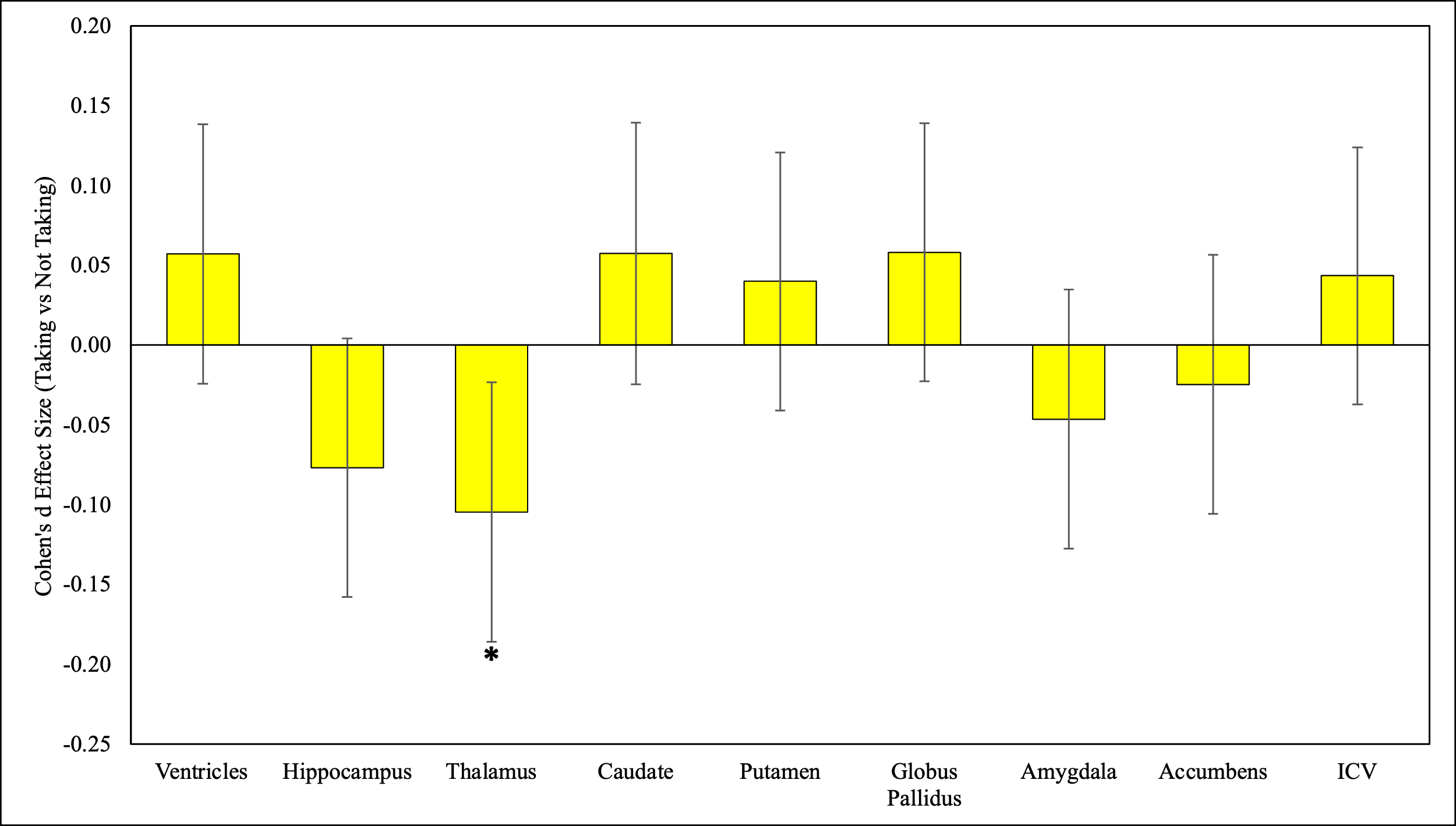


*Note.* Cohen’s d estimates of subcortical volumetric differences comparing patients taking antipsychotics to patients not taking antipsychotics while controlling for age, sex, ICV (for subcortical structures) and psychotropic medication (lithium, antiepileptics, and antidepressants). Error bars show 95% confidence intervals. Black asterisk (*) indicates a q-value of < 0.05.

# Supplementary Figure 6. Subcortical volumetric differences between patients taking (n = 840) and not taking (n = 1574) antidepressants


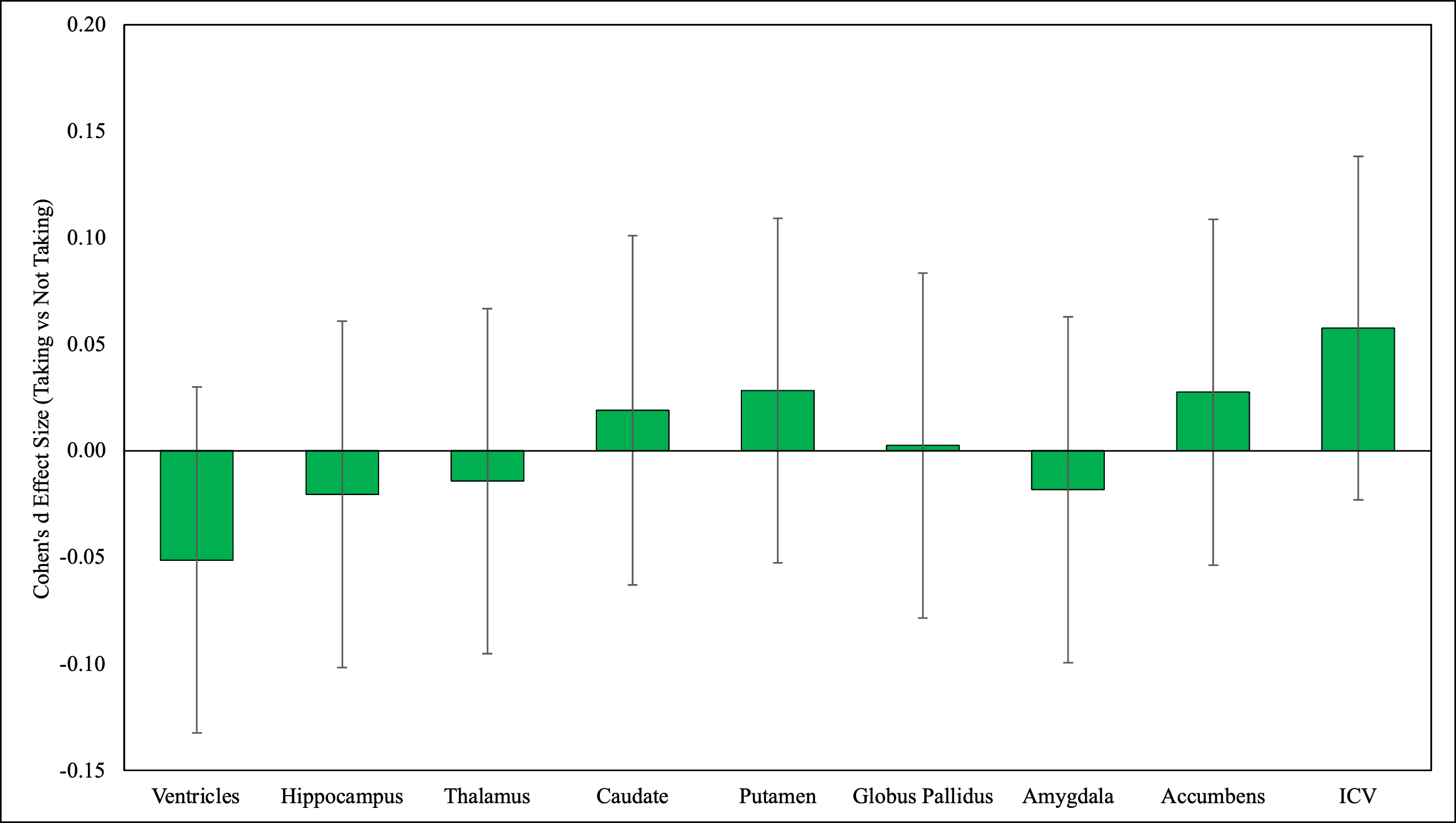


*Note.* Cohen’s d estimates of subcortical volumetric differences comparing patients taking antipsychotics to patients not taking antipsychotics while controlling for age, sex, ICV (for subcortical structures) and psychotropic medication (lithium, antiepileptics, and antidepressants). Error bars show 95% confidence intervals. Black asterisk (*) indicates a q-value of < 0.05.

# Supplementary Figure 7. Concurrent lithium use moderates the association between antiepileptic use and lower hippocampal volume


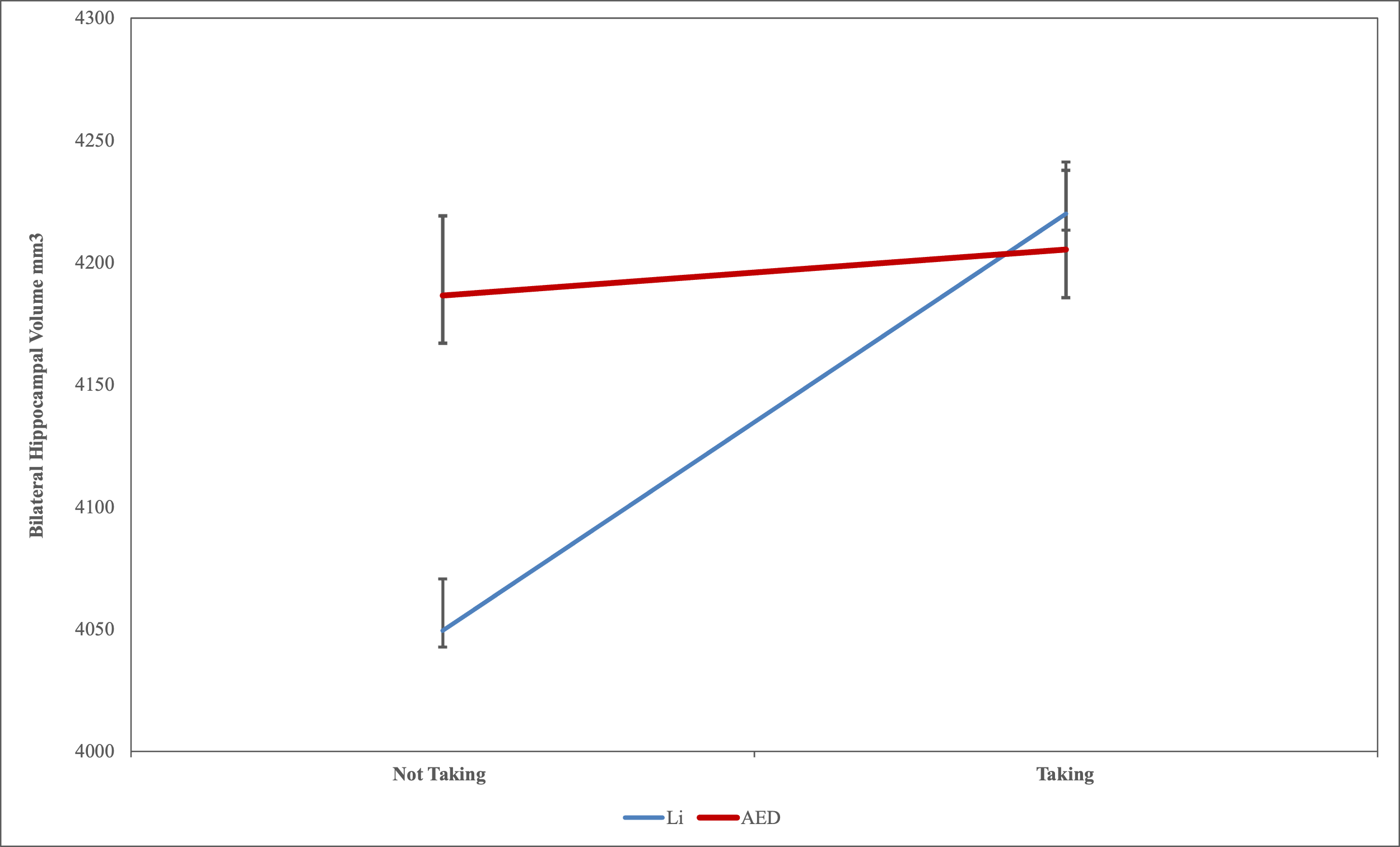


*Note*. Li = Lithium, AED = Antiepileptics, Error bars show 95% confidence intervals.

# Supplementary Table 1. ENIGMA – Bipolar Disorder Working Group Demographics – case-control breakdown for participating sites

| **Cohort** | **Mean Age (SD) CTL** | **Mean Age (SD) BD** | **Mean Age of Onset** | **# Female/Male CTL** | **# Female/Male BD** | **# CTL** | **# BD** | **# BDI/BDII/BDNOS** | **Total N** |
| --- | --- | --- | --- | --- | --- | --- | --- | --- | --- |
| GBS | 36.4 (11.5) | 39.8 (10.7) | 28.0 (8.0) | 38/58 | 35/33 | 96 | 68 | 68/0/0 | 164 |
| SCDS | 34.2 (10.9) | 34.0 (10.7) | 29.7 (10.4) | 23/20 | 27/19 | 43 | 46 | 46/0/0 | 89 |
| CIAM | 26.6 (4.9) | 29.6 (5.2) | 21.5 (4.4) | 14/17 | 10/15 | 31 | 25 | 0/1/0 | 56 |
| MoodInflame | 38.5 (16.4) | 44.5 (10.5) | 20.3 (7.6) | 12/12 | 12/10 | 24 | 22 | 22/0/0 | 46 |
| SBP | 39.1 (14.6) | 40.4 (12.4) | 18.9 (11.1) | 43/40 | 85/51 | 83 | 136 | 77/59/0 | 219 |
| CHRM2_Galway | 40.6 (13.5) | 43.5 (12.4) | 26.8 (9.7) | 32/24 | 22/22 | 56 | 44 | 36/8/0 | 100 |
| GIPSI | NaN | 40.3 (11.7) | 22.6 (10.1) | NaN | 55/30 | NaN | 85 | 77/8/0 | 85 |
| IGP | 36.0 (11.0) | 36.7 (12.2) | 21.8 (9.3) | 32/38 | 49/25 | 70 | 74 | NaN | 144 |
| MALT | 31.2 (9.1) | 34.4 (7.3) | 16.0 (5.8) | 26/18 | 32/12 | 44 | 44 | 0/44/0 | 88 |
| Barcelona | 41.3 (9.6) | 41.7 (9.4) | 25.8 (8.6) | 62/55 | 57/45 | 117 | 102 | NaN | 219 |
| Uni_British_Columbia | 23.4 (4.7) | 22.6 (4.7) | 17.7 (5.0) | 23/24 | 35/32 | 47 | 67 | 67/0/0 | 114 |
| BiDirect | 50.8 (7.8) | 50.8 (6.9) | NaN | 26/18 | 26/18 | 44 | 44 | NaN | 88 |
| SBA | 36.8 (10.4) | 37.8 (9.6) | 22.0 (9.6) | 34/23 | 21/19 | 57 | 40 | 39/0/0 | 97 |
| San_Raffaele | 32.4 (12.3) | 46.9 (11.6) | 30.8 (10.1) | 100/95 | 214/103 | 195 | 317 | 226/79/9 | 512 |
| COGSBD | 36.2 (11.7) | 37.8 (11.3) | 22.6 (9.7) | 19/13 | 29/34 | 32 | 63 | 59/3/0 | 95 |
| Brazil_USP | 28.0 (7.4) | 32.4 (8.5) | 25.3 (5.8) | 37/36 | 79/38 | 73 | 117 | 105/12/0 | 190 |
| OSLO_TOP | 34.8 (9.7) | 34.8 (11.6) | 22.0 (9.3) | 144/158 | 112/81 | 302 | 193 | 117/62/8 | 495 |
| Sydney | 22.3 (3.9) | 25.0 (3.7) | 15.2 (3.3) | 55/65 | 17/43 | 120 | 60 | 34/26/0 | 180 |
| Houston | 24.3 (14.9) | 28.2 (14.4) | 16.4 (6.5) | 94/79 | 153/110 | 173 | 263 | 163/58/42 | 436 |
| Penn | 37.4 (14.3) | 30.0 (10.3) | 19.2 (8.6) | 44/44 | 36/22 | 88 | 58 | 46/7/5 | 146 |
| Yale | 36.4 (13.6) | 34.8 (11.6) | 18.9 (7.6) | 157/89 | 66/38 | 246 | 104 | 99/1/0 | 350 |
| Halifax | 38.9 (13.5) | 48.4 (14.3) | 21.8 (6.8) | 44/24 | 59/44 | 68 | 103 | 71/32/0 | 171 |
| Paris | 36.1 (12.0) | 37.5 (12.7) | 22.7 (8.2) | 33/22 | 12/24 | 55 | 36 | 24/7/1 | 91 |
| MinnAdoIBP | 15.8 (1.8) | 16.0 (1.8) | 8.9 (3.4) | 26/28 | 25/20 | 54 | 45 | 19/16/10 | 99 |
| Marburg | 34.8 (12.8) | 43.7 (10.8) | 24.1 (11.2) | 257/154 | 31/17 | 411 | 48 | 31/15/0 | 459 |
| Muenster | 28.3 (10.3) | 41.7 (13.2) | 25.8 (12.4) | 142/80 | 7/18 | 222 | 25 | 8/17/0 | 247 |
| CAMH | NaN | 17.3 (1.4) | 14.8 (2.7) | NaN | 51/28 | NaN | 79 | 30/22/27 | 79 |
| AFFDIS | 39.9 (14.4) | 43.2 (9.2) | 26.4 (9.5) | 22/24 | 8/17 | 46 | 25 | 24/0/0 | 71 |
| Tulsa | 34.7 (10.5) | 40.6 (11.8) | NaN | 56/35 | 56/13 | 91 | 69 | 34/27/5 | 160 |
| HMS | 39.6 (12.2) | 43.2 (12.2) | 28.2 (9.6) | 34/21 | 20/21 | 55 | 41 | 41/0/0 | 96 |
| MNC | 35.4 (12.1) | 38.3 (11.8) | 26.2 (8.8) | 416/312 | 28/26 | 728 | 54 | 41/13/0 | 782 |
| MUSC | 38.7 (10.4) | 37.5 (12.2) | NaN | 13/13 | 36/33 | 26 | 69 | NaN | 95 |
| CLING | 25.2 (5.3) | 40.6 (10.5) | 26.7 (9.7) | 191/132 | 23/15 | 323 | 38 | 34/1/1 | 361 |
| PADUA | 41.5 (13.1) | 42.1 (13.0) | NaN | 19/26 | 20/40 | 45 | 60 | NaN | 105 |

# Supplementary Table 1. ENIGMA – Bipolar Disorder Working Group Demographics – case-control breakdown for participating sites

| **Cohort** | **Mean Age (SD) CTL** | **Mean Age (SD) BD** | **Mean Age of Onset** | **# Female/Male CTL** | **# Female/Male BD** | **# CTL** | **# BD** | **# BDI/BDII/BDNOS** | **Total N** |
| --- | --- | --- | --- | --- | --- | --- | --- | --- | --- |
| GBS | 36.4 (11.5) | 39.8 (10.7) | 28.0 (8.0) | 38/58 | 35/33 | 96 | 68 | 68/0/0 | 164 |
| SCDS | 34.2 (10.9) | 34.0 (10.7) | 29.7 (10.4) | 23/20 | 27/19 | 43 | 46 | 46/0/0 | 89 |
| CIAM | 26.6 (4.9) | 29.6 (5.2) | 21.5 (4.4) | 14/17 | 10/15 | 31 | 25 | 0/1/0 | 56 |
| MoodInflame | 38.5 (16.4) | 44.5 (10.5) | 20.3 (7.6) | 12/12 | 12/10 | 24 | 22 | 22/0/0 | 46 |
| SBP | 39.1 (14.6) | 40.4 (12.4) | 18.9 (11.1) | 43/40 | 85/51 | 83 | 136 | 77/59/0 | 219 |
| CHRM2_Galway | 40.6 (13.5) | 43.5 (12.4) | 26.8 (9.7) | 32/24 | 22/22 | 56 | 44 | 36/8/0 | 100 |
| GIPSI | NaN | 40.3 (11.7) | 22.6 (10.1) | NaN | 55/30 | NaN | 85 | 77/8/0 | 85 |
| IGP | 36.0 (11.0) | 36.7 (12.2) | 21.8 (9.3) | 32/38 | 49/25 | 70 | 74 | NaN | 144 |
| MALT | 31.2 (9.1) | 34.4 (7.3) | 16.0 (5.8) | 26/18 | 32/12 | 44 | 44 | 0/44/0 | 88 |
| Barcelona | 41.3 (9.6) | 41.7 (9.4) | 25.8 (8.6) | 62/55 | 57/45 | 117 | 102 | NaN | 219 |
| Uni_British_Columbia | 23.4 (4.7) | 22.6 (4.7) | 17.7 (5.0) | 23/24 | 35/32 | 47 | 67 | 67/0/0 | 114 |
| BiDirect | 50.8 (7.8) | 50.8 (6.9) | NaN | 26/18 | 26/18 | 44 | 44 | NaN | 88 |
| SBA | 36.8 (10.4) | 37.8 (9.6) | 22.0 (9.6) | 34/23 | 21/19 | 57 | 40 | 39/0/0 | 97 |
| San_Raffaele | 32.4 (12.3) | 46.9 (11.6) | 30.8 (10.1) | 100/95 | 214/103 | 195 | 317 | 226/79/9 | 512 |
| COGSBD | 36.2 (11.7) | 37.8 (11.3) | 22.6 (9.7) | 19/13 | 29/34 | 32 | 63 | 59/3/0 | 95 |
| Brazil_USP | 28.0 (7.4) | 32.4 (8.5) | 25.3 (5.8) | 37/36 | 79/38 | 73 | 117 | 105/12/0 | 190 |
| OSLO_TOP | 34.8 (9.7) | 34.8 (11.6) | 22.0 (9.3) | 144/158 | 112/81 | 302 | 193 | 117/62/8 | 495 |
| Sydney | 22.3 (3.9) | 25.0 (3.7) | 15.2 (3.3) | 55/65 | 17/43 | 120 | 60 | 34/26/0 | 180 |
| Houston | 24.3 (14.9) | 28.2 (14.4) | 16.4 (6.5) | 94/79 | 153/110 | 173 | 263 | 163/58/42 | 436 |
| Penn | 37.4 (14.3) | 30.0 (10.3) | 19.2 (8.6) | 44/44 | 36/22 | 88 | 58 | 46/7/5 | 146 |
| Yale | 36.4 (13.6) | 34.8 (11.6) | 18.9 (7.6) | 157/89 | 66/38 | 246 | 104 | 99/1/0 | 350 |
| Halifax | 38.9 (13.5) | 48.4 (14.3) | 21.8 (6.8) | 44/24 | 59/44 | 68 | 103 | 71/32/0 | 171 |
| Paris | 36.1 (12.0) | 37.5 (12.7) | 22.7 (8.2) | 33/22 | 12/24 | 55 | 36 | 24/7/1 | 91 |
| MinnAdoIBP | 15.8 (1.8) | 16.0 (1.8) | 8.9 (3.4) | 26/28 | 25/20 | 54 | 45 | 19/16/10 | 99 |
| Marburg | 34.8 (12.8) | 43.7 (10.8) | 24.1 (11.2) | 257/154 | 31/17 | 411 | 48 | 31/15/0 | 459 |
| Muenster | 28.3 (10.3) | 41.7 (13.2) | 25.8 (12.4) | 142/80 | 7/18 | 222 | 25 | 8/17/0 | 247 |
| CAMH | NaN | 17.3 (1.4) | 14.8 (2.7) | NaN | 51/28 | NaN | 79 | 30/22/27 | 79 |
| AFFDIS | 39.9 (14.4) | 43.2 (9.2) | 26.4 (9.5) | 22/24 | 8/17 | 46 | 25 | 24/0/0 | 71 |
| Tulsa | 34.7 (10.5) | 40.6 (11.8) | NaN | 56/35 | 56/13 | 91 | 69 | 34/27/5 | 160 |
| HMS | 39.6 (12.2) | 43.2 (12.2) | 28.2 (9.6) | 34/21 | 20/21 | 55 | 41 | 41/0/0 | 96 |
| MNC | 35.4 (12.1) | 38.3 (11.8) | 26.2 (8.8) | 416/312 | 28/26 | 728 | 54 | 41/13/0 | 782 |
| MUSC | 38.7 (10.4) | 37.5 (12.2) | NaN | 13/13 | 36/33 | 26 | 69 | NaN | 95 |
| CLING | 25.2 (5.3) | 40.6 (10.5) | 26.7 (9.7) | 191/132 | 23/15 | 323 | 38 | 34/1/1 | 361 |
| PADUA | 41.5 (13.1) | 42.1 (13.0) | NaN | 19/26 | 20/40 | 45 | 60 | NaN | 105 |

*Note.* GBS = Galway Bipolar Study, SCDS = Singapore Cross Diagnostic Study, CIAM = Cape Town, SBP = St. Göran Bipolar Project, GIPSI = Medellín, ICP = Imaging Genetics in Psychosis, MALT = Oslo, SBA = Stanford Bipolar Aggregate, COGSBD = Cognition in Bipolar Disorder, Brasil_USP = University of Sao Paulo, OSLO_TOP = Thematically Organized Psychosis Study, MinnAdolBP = Minnesota Adolescent/Adult (PENS and PHCP), CAMH = Center for Addiction and Mental Health (Toronto), AFFDIS = Affective Disorders Study, HMS = University of Heidelberg, MNC = Münster Neuroimaging Cohort, MUSC = Medical university of South Carolina, CLING = Clinical Neuroscience Göttingen, NaN = Missing Data/Data not collected.

# Supplementary Table 2. Diagnosis and medication information

| **Sample** | **Instrument for Diagnosing** | **Method for obtaining medication information** |
| --- | --- | --- |
| GBS | Structured Clinical Interview for DSMIV-TR-Patient Edition for patients and SCID_NP for controls | Detailed clinical interview outlining dose and duration of all psychotropic medication, supplemented by clinical notes where necessary. |
| SCDS | Structured Clinical Interview for DSM-IV | Detailed clinical interview and review of case notes |
| CIAM | Structured Clinical Interview for DSM-IV for Axis I Diagnoses | Patient interview and Hospital records |
| MoodInflame | MINI 5.0.0 | Detailed clinical interview |
| SBP | Structured Clinical Interview for DSM-IV | Detailed clinical interview and review of clinical notes |
| CHRM2_Galway | Structured Clinical Interview for DSM-IV | Detailed clinical interview |
| GIPSI | Diagnostic Interview for Genetic Studies (DIGS) | Detailed clinical interview |
| IGP | Diagnosis was confirmed using the OPCRIT algorithm applied to interviewer ratings on the DIP, acc. to ICD-10 criteria | Detailed clinical interview |
| MALT | Mini--International Neuropsychiatric Interview (MINI), DSM-IV criteria version 5.0. | Stanley Foundation Network Entry Questionnaire (NEQ). |
| Barcelona | Structured Clinical Interview for DSM-IV and Research Diagnostic Criteria (RDC). | Detailed clinical interview and review of case notes. |
| Uni_British_Columbia | Mini--International Neuropsychiatric Interview (MINI) for DSM-IV and ICD-10 | Detailed clinical interview |
| BiDirect | Mini--International Neuropsychiatric Interview (MINI) vs 5.0, HAM-D, current treatment (in hospital) for an episode | Face-to-face clinical interview with subsequent ATC coding |
| SBA | Structured Clinical Interview for DSM-IV | Detailed clinical interview |
| San_Raffaele | Structured Clinical Interview for DSM-IV | Detailed clinical interview |
| COGSBD | MINI-BD | Detailed clinical interview |
| Brazil_USP | Structured Clinical Interview for DSM-IV for Axis I Diagnoses | Self-report and clinical records |
| OSLO_TOP | Structured Clinical Interview for DSM-IV for Axis I Diagnoses | Structured patient interview, and hospital records |
| Sydney | Diagnostic Interview for Genetic Studies (for 22-30 year-olds); Kiddie-SADS (for 12-21 year-olds) | Patient interview and Adult Health Screening questionnaire |
| Houston | Structured Clinical Interview for DSM-IV for Axis I Diagnoses | Patient interview, plus information from the treating clinical psychiatrist (if available) |
| Penn | Structured Clinical Interview for DSM -IV for Axis I Diagnoses | Combination of self-report and clinician report |
| Yale | Structured Clinical Interview for DSM-IV for Axis I Diagnoses | Medication information was obtained using a standardized interview checklist. |
| Halifax | Structured Clinical Interview for DSM-IV for Axis I Diagnoses; (Halifax): Participants were recruited from patients followed up at a specialized Mood Disorders Program at Dalhousie University, Halifax, NS. The Program is a tertiary care clinic providing consultation services to family physicians and community psychiatrists and following up patients with BD. The diagnostic interviews were performed by pairs of clinicians, according to the Schedule for Affective Disorders and Schizophrenia, Lifetime version (SADS-L) and diagnoses were made according to DSM-IV criteria. | Questionnaire with self and interviewer reporting, in part using validated instruments; (Halifax): Patients had regular follow ups at the clinic, including monitoring of Li levels at least twice per year. Furthermore, we established illness course and treatment response to Li using NIMH life charts (NIMH-LCMTM) |
| Paris | Diagnostic Interview for Genetic Studies | (1) Open question: which medication are you taking? (2) hospital records (for inpatients) |
| MinnAdoIBP |  |  |
| FOR2107-Marburg | Structured Clinical Interview for DSM-IV-TR for Axis I Diagnoses | Self-report and hospital records |
| FOR2107-Muenster | Structured Clinical Interview for DSM-IV-TR for Axis I Diagnoses | Self-report and hospital records |
| CAMH | Psychiatric diagnoses of all participants were determined via the Schedule for Affective Disorders and Schizophrenia for School-Age Children, Present and Lifetime version (KSADS-PL). The Diagnostic and Statistical Manual of Mental Disorders IV was used to define BD type I and II diagnoses. BD-NOS diagnosis was defined using the same operationalized criteria as the Course and Outcome of Bipolar Youth (COBY) Study. | Detailed clinical interview outlining current psychotropic medication, supplemented by clinical notes where necessary. |
| AFFDIS | Patients diagnosed by their psychiatrists with a depressive phase of major depressive disorder (UD group) or a depressive phase of bipolar disorder (BD group) (cf. ICD-10) and sex, age, and education matched HC were recruited by announcements at the local University Psychiatric Hospitals. | Detailed clinical interview |
| Tulsa | Structured Clinical Interview for DSM-IV | Clinical interview |
| HMS | The diagnosis of bipolar I disorder was confirmed by using the German version of the Structured Clinical Interview for DSM-IV. | Patient interview, plus information from the treating clinical psychiatrist (if available) |
| MNC | Structured Clinical Interview for DSM-IV for Axis I Diagnoses | Self-report and hospital records |
| MUSC | Structured Clinical Interview for DSM-IV for Axis I Diagnoses | Clinical Interview |
| CLING | All patients met the diagnostic criteria for BD, type 1 or 2, according to ICD-10 and DSM-IV classification standards. Diagnoses were consented within members of the study group and the treating clinical psychiatrist. | Patient interview, plus information from the treating clinical psychiatrist (if available) |
| PADUA | Structured Clinical Interview for DSM-V | Detailed clinical interview and review of medical notes |

*Note.* GBS = Galway Bipolar Study, SCDS = Singapore Cross Diagnostic Study, CIAM = Cape Town, SBP = St. Göran Bipolar Project, GIPSI = Medellín, ICP = Imaging Genetics in Psychosis, MALT = Oslo, SBA = Stanford Bipolar Aggregate, COGSBD = Cognition in Bipolar Disorder, Brasil_USP = University of Sao Paulo, OSLO_TOP = Thematically Organized Psychosis Study, MinnAdolBP = Minnesota Adolescent/Adult (PENS and PHCP), CAMH = Center for Addiction and Mental Health (Toronto), AFFDIS = Affective Disorders Study, HMS = University of Heidelberg, MNC = Münster Neuroimaging Cohort, MUSC = Medical university of South Carolina, CLING = Clinical Neuroscience Göttingen.

# Supplementary Table 3. Inclusion and Exclusion criteria for each site

| **Sample** | **Criteria for Inclusion/Exclusion** |
| --- | --- |
| GBS | Inclusion criteria: DSM-IV diagnosis of bipolar disorder (patients); age >18 and <60. Exclusion criteria: history of neurological illness (comorbid); lifetime DSM-IV axis 1 disorder or family history of psychotic or affective disorder in first- or second-degree relatives (controls); history of substance and/or alcohol misuse in the past year; learning disability; recent oral steroid use. |
| SCDS | Exclusion criteria were age younger than 21 or older than 65 years, history of neurological disease or brain trauma, and alcohol/substance abuse in the 12 months prior to participation. All patients were diagnosed using DSM-IV, based on a detailed clinical interview and review of case notes. All healthy controls met the same exclusion criteria as the patients, and they were interviewed and excluded if they reported a history of mental illness and/or treatment with psychotropic medication other than non-regular use of benzodiazepines or similar drugs for insomnia. They were also questioned about family history of mental illness and excluded if a first-degree relative had experienced symptoms consistent with major psychiatric disorder and/or had received any form of in- or outpatient psychiatric care. The healthy controls were selected to be matched with the patients on demographic variables and on premorbid intelligence. |
| CIAM | Bipolar disorder participants were required to meet a diagnosis of bipolar I disorder with a significant history of psychosis. Between the ages of 19 and 40. Stable outpatients were recruited, and did not meet either mood polarity at the time of scanning. Were compatible for MRI and EEG imaging. Exclusion included history of epilepsy or seizures, which was an exclusion for EEG testing performed. Exclusion of any participants if presented with a significant/chronic general medication condition, e.g.. HIV, diabetes I/II, high blood pressure. Further for female participants no current/recent/suspected pregnancy or current lactation were allowed to participate. |
| MoodInflame | In the MOODINFLAME study we included adult male and female subjects who were free of inflammation-related symptoms including fever and current or recent infectious or inflammatory disease, uncontrolled systemic disease, uncontrolled metabolic disease or other significant uncontrolled somatic disorders known to affect mood. They did not use somatic medication known to affect mood or the immune system, such as corticosteroids, non- steroid anti-inflammatory drugs and statins. Female candidates who were pregnant or recently gave birth were excluded. Patients and controls did not have a contraindication for MRI scanning. Patients were allowed to continue their regular psycho- pharmacological treatment. They were euthymic at the time of scanning as indicated by an Inventory of Depressive Symptoms - Clinician Version (IDS-C30) score o22 and a Young Mania Rating Scale (YMRS) score o12, respectively. Patients with any other current primary major psychiatric diagnosis were excluded including: schizophrenia, schizoaffective disorder, anxiety disorder and substance use disorders. HC did not have any current or life- time psychiatric diagnosis. |
| SBP | Exclusion criteria: Patients: younger than 18 years, not in euthymic state. Controls: any psychiatric axis I or axis II disorder or neurological conditions, family history of schizophrenia or bipolar disorder in first-degree relatives, drug or alcohol abuse. |
| CHRM2_Galway | CHRM2 Study: Inclusion criteria were 18-65 years of age. A diagnosis of BD (and Euthymia) was confirmed using the Diagnostic and Statistical Manual of Mental Disorders (DSM-IV-TR) Structured Clinical Interview for DSM Disorders (American Psychiatric Association, 1994) conducted by an experienced psychiatrist. Exclusion criteria included neurological disorders, learning disability, comorbid misuse of substance/alcohol and of axis-1 disorders, history of head injury resulting in loss of consciousness for >5 minutes along with a history of oral steroid use in the previous 3 months. Mood symptoms severity was assessed using the Hamilton Anxiety (HARS) and Depression (HDRS-21) Rating Scale and the Young Mania Rating Scale (YMRS) at MRI scanning. Healthy controls had no personal history of a psychiatric illness or history among first-degree relatives, defined using the Structured Clinical Interview for DSM-IV – Non-patient edition (American Psychiatric Association, 1994). |
| GIPSI | Inclusion criteria: Diagnosis of Bipolar Disorder I or II based on DSM-IV-TR criteria, age between 18 and 60 years old, education level between 5 and 16 years. Exclusion criteria: Personal history of neurological disorders, mental disability, autism, electroconvulsive therapy and/or traumatic brain injury. |
| IGP | All participants were fluent English speakers and aged 18-65 years old. In addition to general MR contra-indication, exclusion criteria included an inability to communicate sufficiently in English, a current neurological disorder, life- time head injury with loss of consciousness,  a diagnosis of substance abuse or dependence in the past six months; and/or having been treated with electroconvulsive therapy in the previous six months. |
| MALT | Inclusion criteria patients: A DSM-IV diagnosis of bipolar disorder type II. Exclusion criteria healthy controls: Controls with previous or current psychiatric illness were excluded from the study. The exclusion criteria for all participants were: A.) age younger than 18 or older than 50 years; B.) previous head injury with loss of consciousness for more than 1 minute; C.) history of neurological or other severe chronic somatic disorder; D.) pregnancy; E.) metallic implants. |
| FIDMAG-Barcelona | All patients with bipolar disorder were right handed. Exclusion criteria were age younger than 18 or older than 65 years, history of neurological disease or brain trauma, and alcohol/substance abuse in the 12 months prior to participation. Patients were also required to have a current IQ in the normal range (>70). All patients were diagnosed using DSM-IV and Research Diagnostic Criteria (RDC), based on a detailed clinical interview and review of case notes. All healthy controls met the same exclusion criteria as the patients, and they were interviewed and excluded if they reported a history of mental illness and/or treatment with psychotropic medication other than non-regular use of benzodiazepines or similar drugs for insomnia. They were also questioned about family history of mental illness and excluded if a first-degree relative had experienced symptoms consistent with major psychiatric disorder and/or had received any form of in- or outpatient psychiatric care. The healthy controls were selected to be matched with the patients on demographic variables and on premorbid IQ. |
| Uni_British_Columbia | Inclusion criteria were: aged 14 to 35 years; first episode mania within 3 months of enrollment. Inclusion criteria were deliberately broad to capture a wide range of clinical presentations. Exclusion criteria were: inability to take part in neuropsychological testing; meets the standard criteria for exclusion for magnetic resonance imaging; a previous manic episode diagnosed retrospectively on structured interview or via collateral. Age- and sex-matched healthy controls were also recruited, with the following exclusion criteria for healthy controls: personal history of psychiatric disorder; family history of any major psychiatric disorder in the first or second degree relatives; inability to take part in neuropsychological testing; meets the standard criteria for exclusion for magnetic resonance imaging. |
| BiDirect | All participants were aged 35 to 65 years, participants in the depression cohort were recruited during in hospital treatment for a depressive episode, excluded were those with a concurrent substance use disorder, classification into BD type was done by the treating psychiatrist. Participants in the control cohort were recruited as a random sample drawn in the city register and invited to participate in the study.  The study was approved by the local ethics committee. |
| SBA | To be eligible for the study, individuals had to be between the ages of 18 and 60 years, be fluent English speakers, and meet criteria for Bipolar I Disorder (BD I) or for no lifetime mood disorder using the Structured Clinical Interview for the fourth edition of the Diagnostic and Statistical Manual of Mental Disorders (SCID-IV). Exclusion criteria included a history of alcohol or substance abuse or dependence in the past year, primary psychotic disorders, history of brain injury, hemorrhage, or tumor, stroke, medical conditions influencing the central nervous system, developmental disabilities, electroconvulsive treatment within the past 18 months, weight greater than 300 pounds, pain medication during the 24 h before the scan, diabetes, epilepsy, cardiovascular disorder, loss of consciousness for more than 5 min in the past year or more than 1 h during lifetime, and history of amnesia. Individuals were excluded if they were color blind or dyslexic, reported claustrophobia or any occupational or medical history entailing a risk of a metallic object in their body that could be dislodged by scanning (e.g., work as a metal worker, eye injury involving a metallic object, nonremovable body piercings, metal implants or ear tubes, certain tattoos). Women with late menstrual periods or possible pregnancies were also excluded, as were individuals taking first-generation antipsychotic medications. |
| San_Raffaele | Exclusion criteria were age younger than 18; the presence of other diagnoses on Axis I; the presence of pregnancy; history of epilepsy or major medical, neurological disorders or brain trauma; history of drug or alcohol abuse or dependency. Patients were also required to have a current IQ in the normal range (>70). No patient had received electroconvulsive therapy within 6 months prior to study enrolment. |
| COGSBD | All participants were aged 18-70. Exclusion criteria for all participants included; a history of neurological and neurodegenerative conditions, visual and hearing impairments, history of severe head injury, substance abuse/dependence in the past three months, and difficulties with written or spoken English. Furthermore, BD participants were excluded if they had non-trivial medication changes in the two months prior to assessment, or benzodiazepine use in the 48 hours prior. Healthy controls were excluded if they had a personal or family history of psychiatric illness, or current/previous psychotropic medication use. |
| Brazil_USP | Treatment-naive individuals fulfilling DSM-IV criteria for BD type I or II, at any phase of the illness. Healthy controls were free of any mental disorder and had no history of mood or psychotic disorders among first-degree relatives. Other inclusion/ exclusion criteria for both study groups: Aged between 18 - 45 years; Free of substance use disorders (lifetime); Right-handed; Absence of neurological disorders or any organic disorders that could affect the central nervous system, No history of head trauma with loss of consciousness; No contraindication for MRI scanning. |
| OSLO_TOP | Inclusion criteria: Patients with bipolar spectrum disorder between ages 18-65 recruited from psychiatric departments and outpatient clinics in Oslo as part of the Thematically Organized Psychosis (TOP) Research study. Exclusion criteria were: IQ < 70, a condition better accounted for by substance abuse or somatic illness, having a brain illness or a previous moderate/severe head injury. General: All participants signed a written informed consent. The study was approved by the Regional Committee for Medical Research Ethics and the Norwegian Data Inspectorate, and conducted in accordance with the Helsinki declaration |
| Sydney | Bipolar disorder participants meet DSM-IV criteria for either bipolar I or bipolar II disorder. Control participants meet criteria if no parent or sibling had bipolar I or II disorder, recurrent major depression, schizoaffective disorder, schizophrenia, recurrent substance abuse or any past psychiatric hospitalization; and no parent with a first degree relative had a past mood disorder hospitalization or history of psychosis. All subjects are aged between 12 and 30 years. For those aged between 12 and 21 an adapted version of the Schedule for Affective Disorders and Schizophrenia for School-Age Children – Present and Lifetime Version (K-SADS-BP) was developed specifically for use in the US-Australia collaborative study of young people at genetic risk for BD. For participants aged between 22 and 30 the DIGS (Version 4) is used to measure the current and lifetime presence of axis I DSM-IV disorders. |
| Houston | The study was approved by the Institutional Review Board of The University of Texas Health Science Center at San Antonio. Healthy controls reporting current or past Axis I disorders, suicidal history and a first-degree relative with any Axis I disorder were excluded. Participants with any endocrinological disease, head trauma, neurological disease, and family history of any hereditary neurological disorder or medical conditions such as hypertension, diabetes, active liver disease and kidney problems were excluded from this study. |
| Penn | Inclusion criteria: DSM-IV diagnosis of bipolar disorder; clinically stable, without recent (< 2 week) clinically significant changes in medication type or dose. As long as they are clinically stable, patients may have active illness symptoms, including hallucinations, mood elevation, irritability, or depression. Patients deemed to be at elevated risk of self-harm or violence will be excluded from the study. Age >18 and |
| Yale | (Study 1) Patients were identified through outpatient clinics and community mental health facilities in the Hartford area. Inclusion criteria for patients were age between 18 and 70 years, diagnosis of bipolar I disorder as determined by the Structured Clinical Interview for DSM-IV (SCID), and no first-degree relatives with a bipolar disorder diagnosis. Unrelated healthy comparison subjects were included if they had no lifetime history of axis I psychiatric disorder as assessed by the SCID and no family history of mood or psychotic disorders. Participants were excluded for alcohol or drug abuse or dependence within the past 6 months, a history of major medical or neurological disorders, or IQ <70 as assessed by the WAIS. In patients, euthymia was established with the Hamilton Depression Rating Scale (HAM-D), the Young Mania Rating Scale, the Brief Psychiatric Rating Scale (BPRS), and through diagnostic case reviews. |
|  | (Study 2) Inclusion criteria: ages 15-65 years; proficiency in English at the sixth-grade level or higher; no significant neurologic disorders including those secondary to head injury; no history of substance abuse within the last month or substance dependence within the last 6 months; and negative urine toxicology screening results on the day of testing. The healthy controls met the following additional criteria: no personal or family history (first degree) of psychotic or bipolar disorders; no personal history of recurrent mood disorder; no lifetime history of substance dependence; and no history of any significant cluster A Axis II personality features defined by meeting full criteria or within 1 criterion of a cluster A diagnosis using the Structured Interview for DSM-IV Personality. |
| Halifax | Inclusion criteria. The BD patients (both Li and non-Li groups) had to have: (i) a diagnosis of bipolar I or II disorder made by a psychiatrist using the SCID; (ii) at least 10 years of illness; (iii) a history of at least five episodes of illness (including manic, depressive, or mixed episodes); (iv) current Hamilton Depression Rating Scale, 17-item version (HAM-D-17) score < 7; (v) current Young Mania Rating Scale (YMRS) score < 5; (vi) current Clinical Global Impressions Scale–Bipolar (CGI-BP) score < 3; and (vii) a period of euthymia for at least four months prior to scanning, as aside from state- related factors, patients in acute episodes may present with additional difficult to control confounding variables, including recent medication change or substance abuse. The non-Li group had to have less than three months of lifetime Li exposure, more than 24 months prior to the scanning. The Li group had to have a current Li treatment lasting a minimum of 24 months. Exclusion criteria. Individuals from any of the three groups were excluded if they met any of the magnetic resonance imaging (MRI) exclusion criteria or had any serious medical illness (e.g., brain injury, Cushings disease, or conditions treated with corticosteroids). Individuals with BD were excluded if they had: (i) more than one lifetime course of electroconvulsive therapy (ECT) or ECT in the previous 12 months; (ii) comorbid psychiatric disorders, and ⁄ or personality disorder; (iii) active substance abuse in the previous 12 months; (iv) significant change in their medication in the previous three months; or (v) current psychotic features or acute suicidality. Individuals from the non-Li group were excluded if they had: (i) Li exposure < 2 years before the scanning; or (ii) lifetime Li exposure of more than three months. The neuropsychiatrically healthy individuals were excluded if they had a personal history of psychiatric disorders. (Halifax) Diabetes Study: The subjects with BD were required to 1) have the diagnosis of bipolar I or II disorder made by a psychiatrist; and 2) be at least 18 years of age. Patients were excluded if they had 1) the diagnosis of organic mood disorder; 2) mood disorder not otherwise specified; or 3) more than one lifetime course of electroconvulsive therapy or electroconvulsive therapy within the last 6 months. The neuropsychiatrically healthy, euglycemic subjects were excluded if they had 1) a personal history of psychiatric disorders; or 2) T2DM. Subjects from any group were excluded if they 1) met any magnetic resonance imaging (MRI) exclusion criteria; 2) suffered from substance abuse in the last 12 months; had a history of 3) neurodegenerative disorders; or 4) cerebrovascular disease/stroke, as we were interested in the more subtle T2DM-related neuronal changes. Halifax High Risk Study: Families were identified through adult probands with BD, who had participated in 1) previous genetic and high-risk studies for the Halifax sample. Only the offspring from these families, not the probands, were a part of the MRI study. The offspring from BD parents were divided into two subgroups: 1) the Unaffected HR group, which consisted of 50 offspring with no lifetime history of psychiatric disorders. These individuals were at an increased risk for BD because they had one parent affected with a primary mood disorder. 2) The Affected Familial group, which consisted of 36 offspring who met criteria for a lifetime Axis I diagnosis of mood disorders (i.e., a personal history of at least one episode of depression, hypomania, or mania meeting full DSM-IV criteria). When available, we recruited more than one offspring per family. From this study, we provided data only from patients who had a personal history of bipolar disorder. |
| Paris | Inclusion criteria for study participation were ages between 18 and 65, no history of alcohol or drug abuse/dependence, no history of mental retardation, no previous head trauma with loss of consciousness, and no current or past cardiac or neurological disease. We excluded subjects with any significant cerebral anatomic anomaly. In addition, HC were free of any personal past or present personal psychiatric disorder and first-degree family history of schizophrenia, schizoaffective disorder or BD. Participants were not included for MRI if MRI was contraindicated or if pregnant. |
| MinnAdoIBP | Adolescent study had the following inclusion criteria for all participants: ages 13 to 19, absence of neurological disorders or severe head injury; no major/chronic physical conditions; absence of neuroimaging contraindications due to fMRI requirements; IQ ≥ 70; absence of learning disabilities/developmental problems; normal/corrected-to-normal vision and hearing; native English speaker or bilingual since age 5; and right handedness. Adolescents in BD group required confirmed primary BD diagnosis on diagnostic interview, while healthy control participants required no history of psychopathology and no family history of BD, psychosis, or severe depression.  Adult BD data were collected as part of two family studies focusing on psychosis. PENS recruited adults aged 18–65, including people with major mental illness (schizophrenia, schizoaffective disorder, bipolar I disorder without psychotic features, and bipolar I disorder with psychotic features), their first-degree biological relatives, and unrelated healthy controls. Only patients with a history of psychotic symptoms were included in primary analyses, though a separate analysis was completed comparing participants with bipolar I disorder with and without psychotic features (see below). PHCP (Psychosis Human Connectome Project), recruited people aged 18–65 with a psychotic disorder (schizophrenia, schizoaffective disorder or bipolar I disorder with psychotic features), their first-degree biological relatives (aged 18–69), and unrelated healthy controls. Exclusion criteria for PHCP have been described previously (Demro et al., 2021) and match those of PENS. |
| FOR2107-Marburg | Inclusion criteria: age 18-65 years; patients were diagnosed of bipolar I disorder by SCID-I Interview, currently depressed, (hypo)manic or remitted. Exclusion criteria all: any MRI contraindications; any neurological abnormalities. Exclusion criteria controls: any current or former psychiatric disorder; Exclusion criteria patients: substance dependence or current benzodiazepine treatment (wash out of at least three half-lives before study participation) |
| FOR2107-Muenster | Inclusion criteria: age 18-65 years; patients were diagnosed of bipolar I disorder by SCID-I Interview, currently depressed, (hypo)manic or remitted. Exclusion criteria all: any MRI contraindications; any neurological abnormalities. Exclusion criteria controls: any current or former psychiatric disorder; Exclusion criteria patients: substance dependence or current benzodiazepine treatment (wash out of at least three half-lives before study participation) |
| CAMH | Inclusion criteria: Participants in both groups needed to be English-speaking and able to provide informed consent. For BD participants, they were also required to meet diagnostic criteria for BD-I, BD-II or BD-NOS. Exclusion criteria: Participants in both groups were excluded if they had an existing cardiac condition, diabetes, auto-immune or inflammatory illness, and/or related pharmacological treatment for any of these conditions. Other exclusion criteria were the presence of MRI contraindications, self-reported infectious illness in the past 14 days, and history of drug dependence in the past 3 months. Additional exclusion criteria for CG participants were lifetime history of MDD, BD or psychosis, and first- or second-degree family history of BD or psychosis. |
| AFFDIS | All participants were between 18 and 65 years old. Exclusion criteria were contraindications for MRI scans (i.e. pregnancy, metallic implants), strong visual impairment, neurological disorder, brain surgery or head trauma, current substance abuse, past or present psychiatric disorder for HC, and comorbid psychiatric diagnosis other than anxiety disorders for BD and UD. |
| Tulsa | Bipolar disorder participants met DSM-IV criteria for either bipolar I or bipolar II disorder, or BD NOS. Age 18-55. The unmedicated BD group did not receive any psychotropic medications for at least 3 weeks (8 for fluoxetine) prior to the MRI scanning. The healthy control individuals met the same exclusion criteria except that they had no personal or family (first-degree relatives) history of psychiatric illness assessed using the Structured Clinical Interview for the DSM-IV-TR and the Family Interview for Genetic Studies (FIGS). Exclusion criteria were as follows: serious suicidal ideation or behavior; medical conditions or concomitant medications likely to influence CNS or immunological function including cardiovascular, respiratory, endocrine and neurological diseases; a history of drug or alcohol abuse within 6 months or a history of drug or alcohol dependence within 1 year (DSM-IV-TR criteria), and general MRI exclusion criteria such as magnetic implants or claustrophobia. |
| HMS | Diagnosis according to DSM-IV criteria was obtained using the German version of the Structural Clinical Interview for DSM-IV assessed by the study psychiatrists from the Psychiatry Unit at Saarland University Hospital and confirmed by the treating psychiatrist. Exclusion criteria for patients and controls were MRI contraindications, organic disorders of the central nervous system (e.g., infectious, toxic or cerebrovascular disease, traumatic brain injury and epilepsy) or mental retardation. Exclusion criteria for healthy controls were past or present psychiatric, neurological or medical disorder and a positive family history of psychiatric disorders. |
| MNC | Inclusion criteria: age 17-65 years; patients were diagnosed of bipolar I disorder by SCID-Interview, currently depressed (HAMD >= 18); Exclusion criteria all: any MRI contraindications; any neurological abnormalities; Exclusion criteria controls: any current or former psychiatric disorder; Exclusion criteria patients: substance-related disorders or current benzodiazepine treatment (wash out of at least three half-lives before study participation), and former electroconvulsive therapy |
| MUSC | Individuals meeting *DSM-IV-TR* criteria for BD and/or alcohol dependence (approximating moderate to severe AUD in *DSM-5*, herein referred to as *AUD*) were enrolled into BD + AUD, AUD alone, and BD alone groups. Demographically matched healthy controls (HCs) free from psychiatric disorder were concurrently enrolled. Race and ethnicity data were collected by self-report. General inclusion criteria required participants to be between 18 and 65 years of age, capable of consenting and completing study procedures, and able to abstain from alcohol and drugs for 1 week or longer, verified by serial biomarker testing, before MRI scanning to avoid potential confounding effects of intoxication or withdrawal. Group-specific inclusion/exclusion criteria required participants with BD + AUD and participants with AUD to meet *DSM-IV-TR* criteria for current (past 3 months) AUD and exceed National Institute on Alcohol Abuse and Alcoholism (NIAAA)–defined heavy alcohol drinking levels (ie, >14 [men] or >7 [women] alcohol drinks/week) (on average) in the month before enrollment or, if abstinent at intake, the month preceding their last alcohol drink (ie, because inpatient referrals were coming from a controlled environment). Participants with BD + AUD and those with AUD with co-occurring (past 3 months) drug dependence (approximating moderate to severe SUD) were also included for feasibility and generalizability. Psychotropic medications were permitted for participants with BD + AUD and those with BD but additions, discontinuations, or dose changes more than 20% made 1 week or less prior to MRI were exclusionary. Finally, exceeding NIAAA-defined heavy alcohol drinking levels, reporting frequent (>weekly) past-month drug use, or meeting criteria for any non tobacco-related SUD excluded potential participants with BD and HC participants. General exclusion criteria included serious medical illness; clinically significant head injury; past-month electroconvulsive therapy; psychotic disorder; recurrent major depressive disorder; past-month posttraumatic stress disorder, obsessive-compulsive disorder, or eating disorder; current benzodiazepine or antidipsotropic use (eg, naltrexone, acamprosate, disulfiram); history of delirium tremens or more than 1 withdrawal seizure; significant withdrawal (>7 on the Clinical Interview for Withdrawal Assessment for Alcohol–Revised) and/or medical detoxification 2 weeks or less of MRI; claustrophobia; or MRI incompatible surgical implants or (non)ferrous materials |
|  |  |
| CLING | Patients had to meet the diagnostic criteria for BD, type 1, according to ICD-10 and DSM-IV classification standards. Diagnoses were consented within members of the study group and the treating clinical psychiatrist. The healthy controls exhibited no past or present psychiatric or neurological disorder and had no positive family history of psychiatric disorders. Exclusion criteria in general were lifetime diagnoses of substance dependence, substance abuse during the last month, cannabis abuse during the last 2 weeks, mental retardation, dementia, and neurological illnesses. |
| PADUA | The structured clinical interview for DSM-5-Patient Edition (SCID-5) was used for diagnosis of BD type I and II, and patients were included if on stable pharmacological treatment for at least 1 month. A family history of severe mental illness or a current diagnosis of psychiatric disorders or drug treatment (excluding contraceptive pills in women) were exclusion criteria for HC. Participants were excluded if they were younger than 18 or older than 65 years, if they had a lifetime drug dependence, a history of alcohol or drug abuse in the six months before the study, previous traumatic head injury with loss of consciousness, past or present major medical illness, neurological disorders, and intellectual disability. |

*Note.* GBS = Galway Bipolar Study, SCDS = Singapore Cross Diagnostic Study, CIAM = Cape Town, SBP = St. Göran Bipolar Project, GIPSI = Medellín, ICP = Imaging Genetics in Psychosis, MALT = Oslo, SBA = Stanford Bipolar Aggregate, COGSBD = Cognition in Bipolar Disorder, Brasil_USP = University of Sao Paulo, OSLO_TOP = Thematically Organized Psychosis Study, MinnAdolBP = Minnesota Adolescent/Adult (PENS and PHCP), CAMH = Center for Addiction and Mental Health (Toronto), AFFDIS = Affective Disorders Study, HMS = University of Heidelberg, MNC = Münster Neuroimaging Cohort, MUSC = Medical university of South Carolina, CLING = Clinical Neuroscience Göttingen.

# Supplementary Table 4. Image acquisition and processing details by site

| **Site** | **FreeSurfer** | **MRI** | **Sequence** | **Direction** | **Slices** | **Gap (mm)** | **Voxels (mm)** | **TI (ms)** | **TE (ms)** | **TR (ms)** | **Flip angle** |
| --- | --- | --- | --- | --- | --- | --- | --- | --- | --- | --- | --- |
| GBS | V5.3 | 1.5T Siemens Magnetom | 3D T1-weighted magnetization prepared rapid acquisition on gradient echo (MPRAGE) | Axial | 256 | 0 | .45 x .45 x .9 | 600 | 4.38 | 1140 | 15 |
| SCDS | V5.3 | 3D T1-weighted magnetization prepared rapid acquisition gradient echo (MPRAGE) | 3T Philips Achieva | Axial | 180 | 0 | 1 x 1 x 1 | 600 | 3.3 | 7.2 | 8 |
| CIAM | V5.3 | 3T Siemens Allegra | 3D T1-weighted magnetization prepared rapid acquisition on gradient echo (MPRAGE) | Sagittal | 129 | 0 | 1.3 x 1.0 x 1.3 | 1100 | 1.53; 3.21; 4.89; 6.57 | 2530 | 7 |
| MoodInflame | V5.3 | 3D ultrafast spoiled gradient echo sequence (SPGR) | 3T Philips Intera | Axial | 130 | 0 | .859 x .859 x 1.2 | NA | 4.59 | 9.76 | 8 |
| SBP | V5.1 | 3D‐SPGR | 1.5 T Signa Excite | Coronal | 128 | 1.8 | .7 x .7 x 1.8 | NA | 6 | 21 | 30 |
| CHRM2_Galway | V5.3 | 3D T1-weighted MPRAGE | 3T Philips Achieva Philips | Axial | 180 | 0 | .83 x .83 x .89 | NA | 3.046 | 8.5 | 8 |
| GIPSI | V5.3 | 3T Philips Achieva Philips | 3D T1-weighted TFE | Axial | 160 | 0 | 1 x .6 x .6 | NA | 2.06 | 4.76 | 8 |
| IGP | V5.3 | 3T Achieva Philips TX | 3D T1- weighted magnetization prepared rapid acquisition gradient echo (MPRAGE) | Sagittal | 200 | 0 | 0.9x0.9x0.9 | NA | 4.1 | 8.9 | 8 |
| MALT | NA | 3T Philips Achieva | 3D T1- weighte d turbo field echo (TFE) | Sagittal | 220 | 0 | 1x1x1 | - | 2.3 | 8.4 | 7 |
| FIDMAG-Barcelona | V5.3 | 1.5T GE Signa | 3D T1-weighted enhanced fast gradient echo (EFGRE3D) | Axial | 180 | 0 | .47 x .47 x 1 | 710 | 3.93 | 2000 | 15 |
| Uni_British_Columbia | V5.3 | 3T Philips Achieva | 3D T1 TFE | Axial | 180 | 0 | 1.0 x 1.0 x 1.0 | 794 | 3.5 | 7.6 | 8 |
| BiDirect | V5.3 | 3T Philips Intera | 3D T1-weighted turbo field echo (TFE) | Sagittal | 160 | 0 | 1x1x1 | NA | 3.56 | 7.26 | 9 |
| SBA | V5.3 | 1.5T GE Signa | spoiled gradient echo (SPGR) pulse sequence | Sagittal | 116 |  | 0.86 x 0.86 x 1.5 | 300 | 1.7-3.0 | 8.3-10.1 | 15 |
| San_Raffaele | V6.0 | 3.0 Tesla scanner (Gyroscan Intera, Philips,) | 3D T1-weighted enhanced fast field echo | Axial | 220 | 0.8 | .9 x .9 x 1.6 acq; .9 x .9 x .8 rec | NA | 4.6 | 25 | 30 |
| COGSBD | V6 | 3T Siemens Magnetom  TrioTim | MPRAGE | Sagittal | 176 | 0 | 1x1x1 | 900 | 2.52 | 1900 | 9 |
| Brazil_USP | N/A | 1.5T Siemens Espree | 3D T1- weighted magnetization prepared rapid acquisition gradient echo (MPRAGE) | Sagittal | 160 | 0 | 1.3x1.3x1.2 | NA | 3.65ms | 2400 | 8 |
| OSLO_TOP | V5.1 | 3T Philips Achieva | 3D T1-weighted turbo field echo (TFE) | Sagittal | 220 | 0 | 1 x 1 x 1 | NA | 2.3 | 8.4 | 7 |
| Sydney | V5.3 | 3T Philips Achieva | 3D T1-weighted turbo field echo (TFE) | Sagittal | 180 | 1 | 1 x 1 x 1 | NA | 2.5 | 5.5 | 8 |
| Houston | V5.3 | 1.5T Philips Gyroscan Intera | 3D T1-weighted spoiled gradient recalled acquisition in steady state | Sagittal | 144 | 1 | 1x1x1 | 220 | 5 | 2 | 40 |
| Penn | V5.0 | 3T Siemens Tim Trio | 3D T1-weighted magnetization prepared rapid acquisition gradient echo (MPRAGE) | Axial | 200 | 0 | 0.9x0.9x1 | 1100 | 3.51 | 1810 | 8 |
| Yale | V5.3 | 3T Siemens Allegra | 3D T1-weighted magnetization prepared rapid acquisition gradient echo (MPRAGE) | Sagittal | 160 | 0 | 1x1x1.2 | 900 | 2.91 | 2300 | 9 |
| Halifax | V5.3 | 1.5T GE Signa | 3D T1-weighted spoiled gradient recalled acquisition in steady state | Coronal | 125 | 1.5 | .9375 x .9375 x 1.5 | 0 | 5 | 25 | 40 |
| Paris | V5.3 | 3T Siemens Tim Trio | 3D T1-weighted magnetization prepared rapid acquisition gradient echo (MPRAGE) | Sagittal | 160 | 0 | 1x1x1.1 | 900 | 2.98 | 2300 | 9 |
| MinnAdoIBP | NA | Siemens  3 Tesla Prisma | AdolBP:T1-weighted MPRAGE sequence  PENS: T1-weighted MPRAGE sequence  PHCP: T1-weighted MPRAGE sequence | AdolBP: Coronal  PENS:  PHCP: Sagittal | AdolBP: 240  PENS: 256  PHCP: 208 | 0 | AdolBP: 1x1x1  PENS:  PHCP: .8x.8x.8 | AdolBP: 1100  PENS:  PHCP: | AdolBP: 3.65  PENS: 2.12  PHCP: 1.81, 3.6, 5.39, 7.18 | AdolBP: 1530  PENS:2400  PHCP: 2500 | AdolBP: 7  PENS:8  PHCP: 8 |
| FOR2107-Marburg | V5.3 | 3T Siemens Magnetom Trio | 3D T1-weighted magnetization prepared rapid acquisition on gradient echo (MPRAGE) | Sagittal | 176 | 0.5 | 1 x 1 x 1 | 900 | 2.26 | 1900 | 9 |
| FOR2107-Muenster | V5.3 | 3T Siemens PRISMA | 3D T1-weighted magnetization prepared rapid acquisition on gradient echo (MPRAGE) | Sagittal | 192 | 0 | 1 x 1 x 1 | 900 | 2.28 | 2130 | 8 |
| CAMH | V6.0 | 3 T Philips Achieva scanner | 3D fast spoiled gradient echo | Axial | 140 | 0 | 0.94 × 1.17 × 1.2 | 1400 | 2.3 | 9.5 | 8 |
| AFFDIS | V6.0 | 3T Siemens Magnetom Tim Trio | 3D T1-weighted magnetization prepared rapid acquisition gradient echo (MPRAGE) | Sagittal | 176 | 0 | 1x1x1 | 900 | 3.26 | 2250 | 9 |
| Tulsa | V6.0 | 3T GE MR750 Discovery | MPRAGE | Axial | 186 | 0 | .938 x .0938 x .9 | 725 | 2.01 | 6 | 8 |
| HMS | V5.1 | 1.5T Siemens Sonata | 3D T1-weighted magnetization prepared rapid acquisition gradient echo (MPRAGE) | Sagittal | 176 | 0mm | 1x1x1 | 700 | 3.42 | 1900 | 15 |
| MNC | V5.3 | 3T Philips Gyroscan Intera | 3D Fast gradient echo sequence | Coronal | 320 | 0 | .5 x .5 x .5 | 815 | 3.4 | 7.4 | 9 |
| MUSC | V5.3 | 3T Siemens Magnetom Trio | 3D T1-weighted magnetization prepared rapid acquisition on gradient echo (MPRAGE) | Sagittal | 256 | 0 | 1x1x1 | 900 | 2.26 | 2300 | 8 |
| CLING | V5.1 | 3T Siemens Tim Trio | 3D T1-weighted magnetization prepared rapid acquisition gradient echo (MPRAGE) | Sagittal | 176 | 0mm | 1x1x1 | 900 | 3.26 | 2250 | 9 |
| PADUA | V5.3 | 3T Philips Ingenia | 3D T1-weighted magnetization-prepared rapid gradient-echo sequence | Sagittal | 181 | 0 | 1x1x1 | 593 | 3 | 6676 | 8 |

*Note.* GBS = Galway Bipolar Study, SCDS = Singapore Cross Diagnostic Study, CIAM = Cape Town, SBP = St. Göran Bipolar Project, GIPSI = Medellín, ICP = Imaging Genetics in Psychosis, MALT = Oslo, SBA = Stanford Bipolar Aggregate, COGSBD = Cognition in Bipolar Disorder, Brasil_USP = University of Sao Paulo, OSLO_TOP = Thematically Organized Psychosis Study, MinnAdolBP = Minnesota Adolescent/Adult (PENS and PHCP), CAMH = Center for Addiction and Mental Health (Toronto), AFFDIS = Affective Disorders Study, HMS = University of Heidelberg, MNC = Münster Neuroimaging Cohort, MUSC = Medical university of South Carolina, CLING = Clinical Neuroscience Göttingen.

# Supplementary Table 5a. Pharmacological domains and mechanisms of action of NbN

| **Pharmacological Domain** | **Mode of action** |
| --- | --- |
| 1. Acetylcholine | 1. Enzyme inhibitor |
| 1. Dopamine | 2. Enzyme modulator |
| 1. GABA | 3. Ion channel blocker |
| 1. Glutamate | 4. Neurotransmitter releaser |
| 1. Histamine | 5. Positive allosteric modulator |
| 1. Melatonin | 6. Receptor agonist |
| 1. Norepinephrine | 7. Receptor antagonist |
| 1. Opioid | 8. Receptor partial agonist |
| 1. Orexin | 9. Reuptake inhibitor |
| 1. Serotonin |  |

# Supplementary Table 5b. NbN classification of psychotropic medication

| **Indication-based label** | **Medication Name** | **Pharmacological Domain** | **Mechanism of Action** | **ATC code** |
| --- | --- | --- | --- | --- |
| **Antiepileptic Mood stabilizers** | Lamotrigine | glutamate | voltage-gated sodium channel blocker | N03AX09 |
|  | Carbamazepine | glutamate | voltage-gated sodium and calcium channel blocker | N03AF01 |
|  | Gabapentin | glutamate | voltage-gated calcium channel blocker | N03AX12 |
|  | Oxcarbazepine | glutamate | voltage-gated sodium and calcium channel blocker | N03AF02 |
|  | Sodium valproate | glutamate | yet to be determined | N03AG01; N03AG02 |
|  | Topiramate | glutamate, GABA | unclear | N03AX11 |
|  | Pregabalin | glutamate | voltage-gated calcium channel blocker | N03AX16 |
|  | Lithium | inhibition of inositol monophosphatase adenylyl-cyclase, GMP, glycogen synthase kinase 3; increases activity of serotonin and acetylcholine in animal models; modulator of intracellular signaling cascades | enzyme modulator | N05AN01 |
| **First Generation Antipsychotics** | Haloperidol | dopamine | receptor antagonist | N05AD01 |
|  | Flupentixol | dopamine, serotonin | receptor antagonist | N05AF01 |
|  | Zuclopenthixol | dopamine | receptor antagonist | N05AF05 |
|  | Levomepromazine | dopamine, serotonin | receptor antagonist | N05AA02 |
|  | Chlorpromazine | dopamine, serotonin | receptor antagonist | N05AA01 |
|  | Trifluoperazine | dopamine, serotonin | receptor antagonist | N05AB06 |
|  | Pipotiazine | dopamine, serotonin | receptor antagonist | N05AC04 |
|  | Perphenazine | dopamine | receptor antagonist | N05AB03 |
|  | Fluphenazine | dopamine | receptor antagonist | N05AB02 |
|  | Loxapine | dopamine, serotonin | receptor antagonist | N05AH01 |
|  | Sulpiride | dopamine | receptor antagonist | N05AL01 |
|  | Thioridazine | dopamine, serotonin | receptor antagonist | N05AC02 |
| **Second Generation Antipsychotics** | Amisulpride | dopamine | receptor antagonist | N05AL05 |
|  | Risperidone | dopamine, serotonin, norepinephrine | receptor antagonist | N05AX08 |
|  | Olanzapine | dopamine, serotonin | receptor antagonist | N05AH03 |
|  | Quetiapine | dopamine, serotonin, norepinephrine | multimodal/dopamine, serotonin, norepinephrine receptor antagonist, reuptake inhibitor (metabolite) | N05AH04 |
|  | Lurasidone | dopamine, serotonin | receptor antagonist | N05AE05 |
|  | Aripiprazole | dopamine, serotonin | receptor partial agonist | N05AX12 |
|  | Asenapine | dopamine, serotonin, norepinephrine | receptor antagonist | N05AH05 |
|  | Paliperidone | dopamine, serotonin, norepinephrine | receptor antagonist | N05AX13 |
|  | Ziprasidone | dopamine, serotonin | receptor antagonist | N05AE04 |
|  | Clozapine | dopamine, serotonin, norepinephrine | receptor antagonist | N05AH02 |
| **Selective Reuptake Inhibitors** | Paroxetine | serotonin | reuptake inhibitor | N06AB05 |
|  | Citalopram | serotonin | reuptake inhibitor | N06AB04 |
|  | Sertraline | serotonin | reuptake inhibitor | N06AB06 |
|  | Escitalopram | serotonin | reuptake inhibitor | N06AB10 |
|  | Fluoxetine | serotonin | reuptake inhibitor | N06AB03 |
|  | Fluvoxamine | serotonin | reuptake inhibitor | N06AB08 |
| **Noradrenergic and Specific Serotonergic Antidepressants** | Mirtazapine | serotonin, norepinephrine | receptor antagonist | N06AX11 |
| **Serotonin-Norepinephrine Reuptake Inhibitors** | Venlafaxine | serotonin, norepinephrine | reuptake inhibitor | N06AX16 |
|  | Duloxetine | serotonin, norepinephrine | reuptake inhibitor | N06AX21 |
| **Serotonin Antagonist and Reuptake Inhibitors** | Trazodone | serotonin | multimodal/ serotonin reuptake inhibitor, receptor antagonist 5-HT1A, receptor antagonist 5-HT2 | N06AX05 |
| **Norepinephrine-Dopamine Reuptake Inhibitors** | Bupropion | norepinephrine, dopamine | reuptake inhibitor, releaser | N06AX12 |
| **Tricyclic Antidepressants** | Clomipramine | serotonin, norepinephrine | reuptake inhibitor | N06AA04 |
|  | Dosulepin | serotonin, norepinephrine | reuptake inhibitor | N06AA16 |
|  | Amitriptyline | serotonin, norepinephrine | multimodal: reuptake inhibitor, receptor antagonist | N06AA09 |
|  | Imipramine | serotonin, norepinephrine | reuptake inhibitor | N06AA02 |
|  | Trimipramine | serotonin, dopamine | receptor antagonist | N06AA06 |
|  | Nortriptyline | norepinephrine | reuptake inhibitor | N06AA10 |
| **Monoamine Oxidase Inhibitors** | Tranylcypromine | serotonin, norepinephrine, dopamine | multimodal: enzyme inhibitor (MAO-a and B), releaser (DA, NE) | N06AF04 |
|  | Moclobemide | serotonin, norepinephrine, dopamine | enzyme inhibitor | N06AG02 |
| **Reversible Inhibitors of Monoamine Oxidase A** | Reboxetine | norepinephrine | reuptake inhibitor | N06AX18 |
|  | Agomelatine | melatonin, serotonin | receptor agonist and antagonist | N06AX22 |
| **Tricyclic Antidepressants + First Generation Antipsychotics** | Etrafon (perphenazine+amitriptyline) | serotonin, norepinephrine, dopamine | multimodal: reuptake inhibitor, receptor antagonist | N06CA01 |
| **NOT LISTED IN NBN** | Opipramol |  |  | N06AA05 |
|  | Pipamperone |  |  | N05AD05 |
|  | Prothipendyl |  |  | N05AX07 |
|  | Imipraminoxide |  |  | N06AA03 |
|  | Clotiapine |  |  | N05AH06 |
|  | Promazine |  |  | N05AA03 |
|  | Melperone |  |  | N05AD03 |
|  | Levetiracetam |  |  | N03AX14 |
|  | Pheneturide |  |  | N03AX13 |
|  | Trifluperidol |  |  | N05AD02 |

**Supplementary Table 5c. Traditional vs Neuroscience based Nomenclature (NbN) categories.**

| **Traditional category** | **NbN category** | **Medication examples** | **N (% of patients)** |
| --- | --- | --- | --- |
| **Antipsychotics** | 1.Primarily dopamine receptor antagonists | Haloperidol, Zuclopenthixol, Perphenazine, Fluphenazine, Sulpiride, Amisulpride. | 79 (2.9%) |
|  | 2.Dopamine and other (Serotonin-Norepinephrine) monoamine receptor antagonists | Flupenthixol, Levomepromazine, Chlorpromazine, Trifluoperazine, Pipotiazine, Loxapine, Thioridazine, Olanzapine, Lurasidone, Ziprasidone, Risperidone, Paliperidone, Asenapine, Quetiapine, Clozapine | 484 (18.2%) |
|  | 3.Dopamine, serotonin receptor partial agonist/antagonist | Aripiprazole | 40 (1.5%) |
| **Antidepressants** | 4. Targeting serotonin (reuptake inhibitors-multimodal) | Escitalopram, Citalopram, Paroxetine, Fluoxetine, Fluvoxamine, Sertraline, Trazodone | 247 (9.2%) |
|  | 5. Targeting serotonin and other monoamines with different mechanisms of action | Mirtazapine, Venlafaxine, Duloxetine, Clomipramine, Dosulepin, Imipramine, Bupropion, Amitriptyline, Nortriptyline, Tranylcypromine, Moclobemide, Reboxetine, Agomelatine | 242 (9.1%) |
| **Antiepileptics** | 6. Glutamate, sodium, calcium channel blockers | Carbamazepine, Oxcarbazepine, Lamotrigine, Pregabalin, Gabapentin | 304 (11.4%) |
|  | 7. Valproate | Valproate compounds (valproic acid, valproate) | 262 (9.8%) |
| **Mood Stabilisers** | 8. Lithium | Lithium compounds (lithium carbonate, lithium sulphate) | 871 (32.7%) |
|  |  |  |  |
| **Anxiolytics and hypnotics** | 9. Positive Allosteric Modulators on GABA | Zopiclone, Zolpidem, Clonazepam, Alprazolam, other benzodiazepines | 308 (11.6%) |
| **Other** | 10. Other not listed or with no clear mechanism of action | Levetiracetam, Pheneturide, Etrafon, Promazine, Trifluperidol, Melperone, Pipamperone, Prothipendyl, Clotiapine, Imipraminoxide, Opipramol, Propiomazine, Amantadine, Atomoxetine, Methylphenidate, Benzhexol, Hydroxyzine, Procyclidine, Topiramate | 83 (3.1%) |

# Supplementary Table 6. Subcortical volumetric differences between patients with bipolar disorder and healthy controls

|  | ***d*** | ***d* 95% CI** | **Std. β** | **Std. β 95% CI** | **SE** | **p-value** | **q-value** | **# BD** | **# CN** |
| --- | --- | --- | --- | --- | --- | --- | --- | --- | --- |
| Ventricles | 0.22 | [.17, .27] | 0.21 | [.16, .26] | 0.03 | <0.0001 | **<0.0001** | 2589 | 3284 |
| Hippocampus | -0.13 | [-.18, -.08] | -0.11 | [-.16, -.07] | 0.02 | <0.0001 | **<0.0001** | 2579 | 4019 |
| Thalamus | -0.09 | [-.14, -.04] | -0.07 | [-.11, -.03] | 0.02 | 0.0002 | **0.0019** | 2604 | 4026 |
| Caudate | 0.02 | [-.02, .07] | 0.02 | [-.14, -.05] | 0.02 | 0.34 | 0.36 | 2601 | 4022 |
| Putamen | 0.02 | [-.03, .07] | 0.02 | [-.02, .05] | 0.02 | 0.37 | 0.38 | 2613 | 4021 |
| Globus Pallidus | -0.02 | [-.06, .03] | -0.01 | [-.05, .02] | 0.02 | 0.52 | 0.45 | 2611 | 4005 |
| Amygdala | -0.05 | [-.09, .00] | -0.04 | [-.08, .00] | 0.02 | 0.06 | 0.13 | 2596 | 3998 |
| Accumbens | -0.03 | [-.08, .02] | -0.02 | [-.06, .02] | 0.02 | 0.26 | 0.30 | 2597 | 4026 |
| ICV | -0.07 | [-.12, -.02] | -0.06 | [-.10, -.02] | 0.02 | 0.0035 | **0.017** | 2628 | 4038 |

*Note.* d = Cohen’s d, β = Standardized beta coefficient, CI = Confidence Intervals, SE= Standard Error, BD = Patients with bipolar disorder, CN = Controls. Sex, Age, and ICV (for subcortical structures) were controlled for in each model.

# Supplementary Table 7. Number of Cases where hemispheric volumes were missing

|  | N Total | N Missing (by hemisphere) | Missing % |
| --- | --- | --- | --- |
| Thalamus | 6671 | 169 | 2.53% |
| Caudate | 6553 | 61 | 0.93% |
| Putamen | 6683 | 101 | 1.51% |
| Pallidus | 6662 | 312 | 4.68% |
| Hippocampus | 6643 | 201 | 3.02% |
| Accumbens | 6671 | 142 | 2.13% |
| Amygdala | 6641 | 102 | 1.54% |

# Supplementary Table 8. Subcortical volumetric differences between patients with bipolar disorder and healthy controls across hemispheres

| Hemisphere | Brain_Region | d | *d 95% CI* | Std. β | Std. β 95% CI | SE | p-value^^[[3]](#footnote-3)^^ | # BD | # HC |
| --- | --- | --- | --- | --- | --- | --- | --- | --- | --- |
| Left | Accumbens | 0.00 | -0.05 ; 0.050 | 0.00 | -0.038 ; 0.04 | 0.02 | 0.946 | 2562 | 3992 |
| Right | Accumbens | -0.06 | -0.11 ; -0.011 | -0.05 | -0.086 ; -0.01 | 0.02 | **0.016** | 2569 | 3982 |
| Left | Amygdala | -0.04 | -0.09 ; 0.004 | -0.04 | -0.082 ; 0.00 | 0.02 | 0.072 | 2569 | 3968 |
| Right | Amygdala | -0.04 | -0.09 ; 0.005 | -0.03 | -0.074 ; 0.00 | 0.02 | 0.081 | 2576 | 3975 |
| Left | Caudate | 0.00 | -0.04 ; 0.052 | 0.00 | -0.040 ; 0.05 | 0.02 | 0.883 | 2588 | 4004 |
| Right | Caudate | 0.04 | -0.01 ; 0.084 | 0.03 | -0.011 ; 0.07 | 0.02 | 0.148 | 2585 | 4009 |
| Left | Globus Pallidus | 0.01 | -0.04 ; 0.058 | 0.01 | -0.030 ; 0.04 | 0.02 | 0.736 | 2511 | 3843 |
| Right | Globus Pallidus | -0.04 | -0.08 ; 0.013 | -0.03 | -0.071 ; 0.01 | 0.02 | 0.146 | 2597 | 3971 |
| Left | Hippocampus | -0.13 | -0.18 ; -0.082 | -0.12 | -0.162 ; -0.07 | 0.02 | **<0.0001** | 2517 | 3971 |
| Right | Hippocampus | -0.11 | -0.16 ; -0.058 | -0.10 | -0.140 ; -0.05 | 0.02 | **<0.0001** | 2542 | 3967 |
| Left | ICV | -0.07 | -0.12 ; -0.024 | -0.06 | -0.103 ; -0.02 | 0.02 | **0.003** | 2628 | 4038 |
| Right | ICV | -0.07 | -0.12 ; -0.024 | -0.06 | -0.103 ; -0.02 | 0.02 | **0.003** | 2628 | 4038 |
| Left | Putamen | 0.02 | -0.03 ; 0.066 | 0.01 | -0.024 ; 0.05 | 0.02 | 0.472 | 2589 | 3985 |
| Right | Putamen | 0.03 | -0.01 ; 0.082 | 0.03 | -0.011 ; 0.06 | 0.02 | 0.171 | 2599 | 3994 |
| Left | Thalamus | -0.08 | -0.13 ; -0.033 | -0.06 | -0.101 ; -0.03 | 0.02 | **0.001** | 2572 | 4014 |
| Right | Thalamus | -0.07 | -0.12 ; -0.024 | -0.06 | -0.098 ; -0.02 | 0.02 | **0.003** | 2533 | 3972 |
| Left | Ventricles | 0.22 | 0.17 ; 0.272 | 0.22 | 0.166 ; 0.27 | 0.03 | **<0.0001** | 2589 | 3283 |
| Right | Ventricles | 0.20 | 0.15 ; 0.250 | 0.19 | 0.144 ; 0.24 | 0.03 | **<0.0001** | 2589 | 3283 |

*Note.* d = Cohen’s d, β = Standardised beta coefficient, CI = Confidence Intervals, SE= Standard Error, BD = Patients with bipolar disorder, CN = Controls. Sex, Age, and ICV (for subcortical structures) were controlled for in each model.

# Supplementary Table 9. Associations between the number of psychotropic medications BD patients are taking at the time of scan and subcortical volume compared with CN

|  | ***d*** | ***d 95% CI*** | **Std. β** | **Std. β 95% CI** | **SE** | **p-value** | **q-value** | **# BD** |
| --- | --- | --- | --- | --- | --- | --- | --- | --- |
| **Not Taking** |  |  |  |  |  |  |  |  |
| Ventricles | 0.07 | [.02, .12] | 0.13 | [.03, .22] | 0.05 | 0.0093 | **0.037** | 407 |
| Hippocampus | -0.01 | [-.06, .04] | -0.02 | [-.10, .07] | 0.04 | 0.71 | 0.52 | 396 |
| Thalamus | -0.01 | [-.06, .04] | -0.01 | [-.09, .07] | 0.04 | 0.79 | 0.54 | 401 |
| Caudate | 0.04 | [-.01, .09] | 0.07 | [-.01, .15] | 0.04 | 0.09 | 0.17 | 402 |
| Putamen | 0.06 | [.01, .11] | 0.09 | [.02, .16] | 0.04 | 0.012 | **0.041** | 401 |
| Globus Pallidus | -0.03 | [-.08, .02] | -0.04 | [-.11, .03] | 0.04 | 0.25 | 0.29 | 402 |
| Amygdala | 0.01 | [-.04, .06] | 0.01 | [-.06, .09] | 0.04 | 0.70 | 0.51 | 401 |
| Accumbens | 0.02 | [-.03, .07] | 0.03 | [-.05, .10] | 0.04 | 0.48 | 0.44 | 399 |
| ICV | -0.06 | [-.11, -.01] | -0.10 | [-.18, -.02] | 0.04 | 0.015 | **0.045** | 407 |
| **Taking One Class** |  |  |  |  |  |  |  |  |
| Ventricles | 0.11 | [.06, .16] | 0.16 | [.08, .23] | 0.04 | <0.0001 | **0.0003** | 751 |
| Hippocampus | -0.08 | [-.12, -.03] | -0.10 | [-.16, -.03] | 0.03 | 0.0028 | **0.015** | 740 |
| Thalamus | -0.06 | [-.11, -.01] | -0.07 | [-.13, -.02] | 0.03 | 0.013 | **0.041** | 750 |
| Caudate | 0.00 | [-.05, .05] | 0.00 | [-.06, .06] | 0.03 | 0.94 | 0.58 | 753 |
| Putamen | 0.00 | [-.05, .05] | 0.00 | [-.06, .05] | 0.03 | 0.90 | 0.57 | 754 |
| Globus Pallidus | 0.00 | [-.05, .05] | 0.00 | [-.06, .05] | 0.03 | 0.92 | 0.58 | 750 |
| Amygdala | -0.02 | [-.07, .03] | -0.02 | [-.08, .04] | 0.03 | 0.48 | 0.44 | 750 |
| Accumbens | 0.00 | [-.05, .05] | 0.00 | [-.05, .06] | 0.03 | 0.91 | 0.57 | 747 |
| ICV | -0.02 | [-.07, .02] | -0.03 | [-.09, .03] | 0.03 | 0.32 | 0.34 | 756 |
| **Taking Two Classes** |  |  |  |  |  |  |  |  |
| Ventricles | 0.19 | [.13, .24] | 0.25 | [.18, .32] | 0.04 | <0.0001 | **<0.0001** | 840 |
| Hippocampus | -0.11 | [-.16, -.06] | -0.14 | [-.20, -.08] | 0.03 | <0.0001 | **<0.0001** | 847 |
| Thalamus | -0.10 | [-.15, -.05] | -0.11 | [-.16, -.06] | 0.03 | <0.0001 | **0.0007** | 855 |
| Caudate | -0.01 | [-.05, .04] | -0.01 | [-.06, .05] | 0.03 | 0.82 | 0.55 | 849 |
| Putamen | -0.01 | [-.06, .04] | -0.01 | [-.06, .04] | 0.03 | 0.78 | 0.54 | 858 |
| Globus Pallidus | -0.02 | [-.07, .03] | -0.02 | [-.07, .03] | 0.03 | 0.47 | 0.43 | 857 |
| Amygdala | -0.07 | [-.12, -.02] | -0.08 | [-.13, -.02] | 0.03 | 0.0071 | **0.030** | 847 |
| Accumbens | -0.04 | [-.09, .01] | -0.04 | [-.10, .01] | 0.03 | 0.097 | 0.17 | 854 |
| ICV | -0.03 | [-.08, .02] | -0.04 | [-.10, .02] | 0.03 | 0.20 | 0.26 | 860 |
| **Taking Three or More Classes** |  |  |  |  |  |  |  |  |
| Ventricles | 0.15 | [.09, .20] | 0.28 | [.18, .38] | 0.05 | <0.0001 | **<0.0001** | 341 |
| Hippocampus | -0.08 | [-.13, -.03] | -0.15 | [-.23, -.06] | 0.04 | 0.0008 | **0.0048** | 348 |
| Thalamus | -0.06 | [-.11, -.01] | -0.10 | [-.18, -.02] | 0.04 | 0.014 | **0.042** | 350 |
| Caudate | 0.02 | [-.03, .07] | 0.03 | [-.05, .11] | 0.04 | 0.50 | 0.44 | 347 |
| Putamen | 0.00 | [-.05, .05] | 0.00 | [-.07, .07] | 0.04 | 0.97 | 0.59 | 350 |
| Globus Pallidus | 0.00 | [-.05, .05] | 0.00 | [-.07, .07] | 0.04 | 0.97 | 0.59 | 352 |
| Amygdala | -0.03 | [-.08, .02] | -0.04 | [-.12, .04] | 0.04 | 0.30 | 0.32 | 348 |
| Accumbens | -0.05 | [-.10, .00] | -0.07 | [-.14, .00] | 0.04 | 0.065 | 0.13 | 348 |
| ICV | -0.01 | [-.06, .04] | -0.01 | [-.10, .07] | 0.04 | 0.76 | 0.53 | 355 |

*Note. d* = Cohen’s d, Std. β = Standardized beta coefficient, CI = Confidence Intervals, SE= Standard Error. Sex, age, and ICV (for subcortical structures) were controlled for in each model.

**Supplementary Table 10. Propensity score matching association of number of psychotropic medications BD patients are taking at the time of the scan with subcortical volume**

| **Contrast** | **ROI** | **d** | **d 95% CI** | **Std. β** | **Std. β 95% CI** | **SE** | **p-value** | **q-value** | **n** |
| --- | --- | --- | --- | --- | --- | --- | --- | --- | --- |
|  |  |  |  |  |  |  |  |  |  |
| Taking One | Accumbens | 0.08 | [-0.10, 0.25] | 0.04 | [-0.05, 0.12] | 5.64 | 0.39 | 0.7250 | 399 |
| Taking One | Amygdala | 0.06 | [-0.11, 0.23] | 0.03 | [-0.06, 0.12] | 11.15 | 0.49 | 0.8350 | 401 |
| Taking One | Caudate | 0.13 | [-0.05, 0.30] | 0.07 | [-0.02, 0.16] | 26.01 | 0.15 | 0.3300 | 402 |
| Taking One | Hippocampus | 0.01 | [-0.16, 0.18] | 0 | [-0.09, 0.10] | 22.98 | 0.92 | 0.9880 | 396 |
| Taking One | Pallidum | -0.12 | [-0.29, 0.05] | -0.06 | [-0.14, 0.02] | 13.13 | 0.17 | 0.3360 | 402 |
| Taking One | Putamen | 0.24 | [0.07, 0.41] | 0.11 | [0.03, 0.19] | 34.06 | 0.01 | **0.0307** | 401 |
| Taking One | Thalamus | -0.04 | [-0.22, 0.13] | -0.02 | [-0.11, 0.06] | 43.34 | 0.62 | 0.9280 | 401 |
| Taking One | Ventricles | 0.15 | [-0.02, 0.32] | 0.09 | [-0.01, 0.20] | 245.92 | 0.08 | 0.1950 | 407 |
| Taking Two | Accumbens | 0.02 | [-0.11, 0.15] | 0.01 | [-0.05, 0.07] | 4.29 | 0.79 | 0.9870 | 854 |
| Taking Two | Amygdala | -0.04 | [-0.17, 0.09] | -0.02 | [-0.09, 0.05] | 8.51 | 0.54 | 0.8670 | 847 |
| Taking Two | Caudate | 0 | [-0.13, 0.14] | 0 | [-0.07, 0.07] | 19.8 | 0.95 | 0.9880 | 849 |
| Taking Two | Hippocampus | -0.17 | [-0.30, -0.04] | -0.09 | [-0.17, -0.02] | 17.5 | 0.01 | **0.0372** | 847 |
| Taking Two | Pallidum | -0.01 | [-0.14, 0.12] | -0.01 | [-0.07, 0.06] | 10.02 | 0.86 | 0.9870 | 857 |
| Taking Two | Putamen | 0 | [-0.13, 0.13] | 0 | [-0.06, 0.06] | 25.87 | 0.99 | 0.9900 | 858 |
| Taking Two | Thalamus | -0.17 | [-0.30, -0.03] | -0.08 | [-0.15, -0.02] | 33 | 0.01 | **0.0405** | 855 |
| Taking Two | Ventricles | 0.28 | [0.15, 0.41] | 0.17 | [0.09, 0.25] | 188.39 | 0.00 | **0.0003** | 840 |
| Taking Three or More | Accumbens | -0.11 | [-0.21, 0.00] | -0.05 | [-0.10, 0.00] | 3.51 | 0.05 | 0.1430 | 348 |
| Taking Three or More | Amygdala | -0.14 | [-0.25, -0.03] | -0.07 | [-0.12, -0.02] | 6.99 | 0.01 | **0.0372** | 348 |
| Taking Three or More | Caudate | 0.01 | [-0.10, 0.12] | 0.01 | [-0.05, 0.06] | 16.28 | 0.86 | 0.9870 | 347 |
| Taking Three or More | Hippocampus | -0.27 | [-0.37, -0.16] | -0.15 | [-0.20, -0.09] | 14.29 | <0.001 | **<0.0001** | **348** |
| Taking Three or More | Pallidum | -0.02 | [-0.12, 0.09] | -0.01 | [-0.06, 0.04] | 8.2 | 0.78 | 0.9870 | 350 |
| Taking Three or More | Putamen | -0.02 | [-0.13, 0.09] | -0.01 | [-0.06, 0.04] | 21.21 | 0.74 | 0.9870 | 352 |
| Taking Three or More | Thalamus | -0.22 | [-0.33, -0.11] | -0.11 | [-0.16, -0.06] | 27.02 | <0.001 | **0.0004** | 350 |
| Taking Three or More | Ventricles | 0.42 | [0.31, 0.53] | 0.26 | [0.19, 0.32] | 156.99 | <0.001 | **<0.0001** | 341 |

*Note.* values represent standardized mean differences in bilateral subcortical volumes derived from weighted linear mixed‐effects models. Medication groups were compared with those taking none, one, two and three or more medications. d = Cohen’s d; Std. β = standardized beta from z-scored models; SE = standard error. Negative values indicate smaller volumes relative to the one-class group. Confidence intervals are shown in brackets. Models adjusted for intracranial volume, age, sex, and site (random intercept).

**Supplementary Table 11. The association of lithium treatment with subcortical volume – BD patients taking lithium vs. CN**

|  | ***d*** | ***d* 95% CI** | **Std. β** | **Std. β 95% CI** | **SE** | **p-value** | **q-value** | **# Taking** | **# CN** |
| --- | --- | --- | --- | --- | --- | --- | --- | --- | --- |
| Ventricles | 0.08 | [.02, .13] | 0.13 | [.04, .21] | 0.04 | 0.0044 | **0.020** | 777 | 3284 |
| Hippocampus | 0.03 | [-.02, .08] | 0.05 | [-.03, .12] | 0.04 | 0.22 | 0.28 | 771 | 4019 |
| Thalamus | 0.06 | [.01, .11] | 0.08 | [.02, .15] | 0.03 | 0.016 | **0.048** | 778 | 4026 |
| Caudate | -0.01 | [-.06, .04] | 0.02 | [-.09, .05] | 0.04 | 0.63 | 0.49 | 773 | 4022 |
| Putamen | 0.03 | [-.02, .08] | 0.04 | [-.03, .10] | 0.03 | 0.26 | 0.30 | 782 | 4021 |
| Globus Pallidus | -0.04 | [-.09, .01] | -0.05 | [-.12, .01] | 0.03 | 0.10 | 0.17 | 782 | 4005 |
| Amygdala | 0.01 | [-.04, .06] | 0.01 | [-.05, .08] | 0.04 | 0.68 | 0.51 | 773 | 3998 |
| Accumbens | -0.02 | [-.06, .03] | -0.02 | [-.08, .04] | 0.03 | 0.54 | 0.46 | 782 | 4026 |
| ICV | -0.03 | [-.08, .02] | -0.04 | [-.11, .03] | 0.04 | 0.30 | 0.32 | 787 | 4038 |

*Note. d* = Cohen’s d, Std. β = Standardized beta coefficient, CI = Confidence Intervals, SE= Standard Error. In each model we controlled for sex, age, ICV (for subcortical structures) and other concurrently used psychotropic medications (antiepileptics, antipsychotics, antidepressants).

# Supplementary Table 12. The association of antiepileptic treatment with subcortical volume – BD patients taking antiepileptics vs. CN

|  | ***d*** | ***d 95% CI*** | **Std. β** | **Std. β 95% CI** | **SE** | **p-value** | **q-value** | **# Taking** | **# CN** |
| --- | --- | --- | --- | --- | --- | --- | --- | --- | --- |
| Ventricles | 0.18 | [.13, .24] | 0.30 | [.21, .39] | 0.04 | <0.0001 | **<0.0001** | 865 | 3284 |
| Hippocampus | -0.09 | [-.14, -.04] | -0.14 | [-.21, -.06] | 0.04 | 0.0004 | **0.0029** | 866 | 4019 |
| Thalamus | -0.14 | [-.19, -.09] | -0.19 | [-.26, -.12] | 0.03 | <0.0001 | **<0.0001** | 878 | 4026 |
| Caudate | -0.03 | [-.08, .02] | -0.05 | [-.12, .02] | 0.04 | 0.1937 | 0.26 | 875 | 4022 |
| Putamen | -0.07 | [-.12, -.02] | -0.09 | [-.15, -.02] | 0.03 | 0.0084 | **0.035** | 879 | 4021 |
| Globus Pallidus | -0.03 | [-.08, .02] | -0.04 | [-.10, .03] | 0.03 | 0.23 | 0.29 | 881 | 4005 |
| Amygdala | -0.04 | [-.09, .01] | -0.06 | [-.13, .01] | 0.04 | 0.090 | 0.17 | 875 | 3998 |
| Accumbens | -0.05 | [-.10, .00] | -0.06 | [-.13, .00] | 0.03 | 0.050 | 0.12 | 877 | 4026 |
| ICV | -0.04 | [-.09, .01] | -0.06 | [-.14, .01] | 0.04 | 0.088 | 0.17 | 883 | 4038 |

*Note. d* = Cohen’s d, Std. β = Standardized beta coefficient, CI = Confidence Intervals, SE= Standard Error. In each model we controlled for sex, age, ICV (for subcortical structures) and other concurrently used psychotropic medications (lithium, antipsychotics, antidepressants).

# Supplementary Table 13. The association of antipsychotic treatment with subcortical volume – BD patients taking antipsychotics vs. CN

|  | ***d*** | ***d 95% CI*** | **Std. β** | **Std. β 95% CI** | **SE** | **p-value** | **q-value** | **# Taking** | **# CN** |
| --- | --- | --- | --- | --- | --- | --- | --- | --- | --- |
| Ventricles | 0.12 | [.06, .17] | 0.19 | [.10, .28] | 0.04 | <0.0001 | **0.0002** | 927 | 3284 |
| Hippocampus | -0.09 | [-.14, -.04] | -0.13 | [-.21, -.06] | 0.04 | 0.0006 | **0.0039** | 937 | 4019 |
| Thalamus | -0.07 | [-.12, -.02] | -0.10 | [-.17, -.03] | 0.03 | 0.0046 | **0.021** | 939 | 4026 |
| Caudate | 0.06 | [.01, .11] | 0.09 | [.02, .16] | 0.04 | 0.017 | **0.049** | 937 | 4022 |
| Putamen | 0.05 | [.00, .10] | 0.07 | [.00, .13] | 0.03 | 0.037 | 0.10 | 942 | 4021 |
| Globus Pallidus | 0.02 | [-.03, .07] | 0.03 | [-.03, .09] | 0.03 | 0.37 | 0.37 | 942 | 4005 |
| Amygdala | -0.03 | [-.08, .02] | -0.04 | [-.11, .03] | 0.04 | 0.30 | 0.32 | 935 | 3998 |
| Accumbens | 0.00 | [-.05, .05] | 0.00 | [-.06, .07] | 0.03 | 0.97 | 0.59 | 939 | 4026 |
| ICV | -0.03 | [-.08, .02] | -0.04 | [-.12, .03] | 0.04 | 0.24 | 0.29 | 949 | 4038 |

*Note. d* = Cohen’s d, Std. β = Standardized beta coefficient, CI = Confidence Intervals, SE= Standard Error. In each model we controlled for sex, age, ICV (for subcortical structures) and other concurrently used psychotropic medications (lithium, antiepileptics, antidepressants).

# Supplementary Table 14. The association of antidepressant treatment with subcortical volume – BD patients taking antidepressants vs. CN

|  | ***d*** | ***d 95% CI*** | **Std. β** | **Std. β 95% CI** | **SE** | **p-value** | **q-value** | **# Taking** | **# CN** |
| --- | --- | --- | --- | --- | --- | --- | --- | --- | --- |
| Ventricles | 0.05 | [-.01, .10] | 0.08 | [-.01, .17] | 0.05 | 0.090 | 0.17 | 757 | 3284 |
| Hippocampus | -0.06 | [-.11, -.01] | -0.10 | [-.18, -.02] | 0.04 | 0.013 | **0.041** | 777 | 4019 |
| Thalamus | -0.05 | [-.10, .00] | -0.07 | [-.14, .00] | 0.04 | 0.064 | 0.13 | 787 | 4026 |
| Caudate | 0.02 | [-.03, .07] | 0.02 | [-.05, .10] | 0.04 | 0.52 | 0.45 | 782 | 4022 |
| Putamen | 0.03 | [-.02, .08] | 0.04 | [-.02, .11] | 0.03 | 0.20 | 0.27 | 791 | 4021 |
| Globus Pallidus | -0.02 | [-.07, .03] | -0.03 | [-.09, .04] | 0.03 | 0.42 | 0.40 | 789 | 4005 |
| Amygdala | -0.03 | [-.08, .02] | -0.05 | [-.12, .02] | 0.04 | 0.19 | 0.26 | 779 | 3998 |
| Accumbens | 0.01 | [-.04, .06] | 0.01 | [-.05, .08] | 0.03 | 0.71 | 0.52 | 787 | 4026 |
| ICV | -0.03 | [-.08, .02] | -0.05 | [-.12, .03] | 0.04 | 0.22 | 0.28 | 796 | 4038 |

*Note*. *d* = Cohen’s d, Std. β = Standardized beta coefficient, CI = Confidence Intervals, SE= Standard Error. In each model we controlled for sex, age, ICV (for subcortical structures) and other concurrently used psychotropic medications (lithium, antiepileptics, antipsychotics).

# Supplementary Table 15. The association of lithium treatment with subcortical volume – BD patients taking lithium vs. CN across hemispheres

| Hemisphere | Brain Region | d | *d 95% CI* | Std. β | Std_Err | Std. β 95% CI | p-value^^[[4]](#footnote-4)^^ | # Taking | # HC |
| --- | --- | --- | --- | --- | --- | --- | --- | --- | --- |
| Left | Accumbens | -0.01 | -0.06;0.04 | -0.02 | 0.03 | -0.08;0.05 | 0.665 | 772 | 3992 |
| Right | Accumbens | -0.02 | -0.07;0.03 | -0.03 | 0.03 | -0.10;0.04 | 0.427 | 771 | 3982 |
| Left | Amygdala | 0.02 | -0.03;0.07 | 0.03 | 0.04 | -0.04;0.11 | 0.418 | 768 | 3968 |
| Right | Amygdala | 0.00 | -0.05;0.05 | 0.00 | 0.04 | -0.07;0.07 | 0.946 | 767 | 3975 |
| Left | Caudate | -0.02 | -0.07;0.03 | -0.03 | 0.04 | -0.10;0.05 | 0.506 | 770 | 4004 |
| Right | Caudate | -0.01 | -0.06;0.04 | -0.01 | 0.04 | -0.09;0.06 | 0.705 | 771 | 4009 |
| Left | Globus Pallidus | -0.02 | -0.07;0.03 | -0.02 | 0.03 | -0.09;0.04 | 0.459 | 759 | 3843 |
| Right | Globus Pallidus | -0.05 | -0.10;0.00 | -0.08 | 0.04 | -0.15;-0.01 | 0.035 | 773 | 3971 |
| Left | Hippocampus | 0.01 | -0.04;0.06 | 0.01 | 0.04 | -0.07;0.09 | 0.835 | 755 | 3971 |
| Right | Hippocampus | 0.04 | -0.01;0.09 | 0.07 | 0.04 | -0.01;0.14 | 0.095 | 761 | 3967 |
| Left | ICV | -0.03 | -0.08;0.02 | -0.04 | 0.04 | -0.11;0.03 | 0.301 | 787 | 4038 |
| Right | ICV | -0.03 | -0.08;0.02 | -0.04 | 0.04 | -0.11;0.03 | 0.301 | 787 | 4038 |
| Left | Putamen | 0.03 | -0.02;0.08 | 0.05 | 0.03 | -0.02;0.11 | 0.184 | 771 | 3985 |
| Right | Putamen | 0.02 | -0.03;0.07 | 0.03 | 0.03 | -0.03;0.09 | 0.330 | 775 | 3994 |
| Left | Thalamus | 0.06 | 0.01;0.11 | 0.08 | 0.03 | 0.01;0.15 | **0.021** | 769 | 4014 |
| Right | Thalamus | 0.07 | 0.02;0.12 | 0.09 | 0.04 | 0.02;0.16 | **0.010** | 757 | 3972 |
| Left | Ventricles | 0.09 | 0.04;0.14 | 0.15 | 0.04 | 0.06;0.24 | **0.001** | 777 | 3283 |
| Right | Ventricles | 0.06 | 0.00;0.11 | 0.09 | 0.04 | 0.01;0.18 | **0.037** | 777 | 3283 |

*Note. d* = Cohen’s d, Std. β = Standardised beta coefficient, CI = Confidence Intervals, SE= Standard Error. In each model we controlled for sex, age, ICV (for subcortical structures) and other concurrently used psychotropic medications (antiepileptics, antipsychotics, antidepressants).

# Supplementary Table 16. The association of antiepileptic treatment with subcortical volume – BD patients taking antiepileptics vs. CN across hemispheres

| **Hemisphere** | **Brain Region** | **d** | ***d 95% CI*** | **Std. β** | **Std_Err** | **Std. β 95% CI** | **p-value^^[[5]](#footnote-5)^^** | **# Taking** | **# HC** |
| --- | --- | --- | --- | --- | --- | --- | --- | --- | --- |
| Left | Accumbens | -0.042 | -0.09;0.01 | -0.06 | 0.03 | -0.13;0.01 | 0.0948 | 861 | 3992 |
| Right | Accumbens | -0.048 | -0.10;0.00 | -0.07 | 0.03 | -0.13;0.00 | 0.0583 | 868 | 3982 |
| Left | Amygdala | -0.044 | -0.09;0.01 | -0.07 | 0.04 | -0.14;0.01 | 0.0826 | 864 | 3968 |
| Right | Amygdala | -0.038 | -0.09;0.01 | -0.05 | 0.04 | -0.12;0.02 | 0.1321 | 870 | 3975 |
| Left | Caudate | -0.048 | -0.10;0.00 | -0.07 | 0.04 | -0.15;0.00 | 0.0570 | 871 | 4004 |
| Right | Caudate | -0.017 | -0.07;0.03 | -0.02 | 0.04 | -0.10;0.05 | 0.5112 | 869 | 4009 |
| Left | Globus Pallidus | -0.011 | -0.06;0.04 | -0.01 | 0.03 | -0.08;0.05 | 0.6599 | 851 | 3843 |
| Right | Globus Pallidus | -0.050 | -0.10;0.00 | -0.07 | 0.04 | -0.14;0.00 | 0.0512 | 875 | 3971 |
| Left | Hippocampus | -0.070 | -0.12;-0.02 | -0.11 | 0.04 | -0.19;-0.03 | **0.0065** | 839 | 3971 |
| Right | Hippocampus | -0.082 | -0.13;-0.03 | -0.13 | 0.04 | -0.20;-0.05 | **0.0014** | 856 | 3967 |
| Left | ICV | -0.043 | -0.09;0.01 | -0.06 | 0.04 | -0.14;0.01 | 0.0883 | 883 | 4038 |
| Right | ICV | -0.043 | -0.09;0.01 | -0.06 | 0.04 | -0.14;0.01 | 0.0883 | 883 | 4038 |
| Left | Putamen | -0.057 | -0.11;-0.01 | -0.08 | 0.03 | -0.14;-0.01 | **0.0253** | 867 | 3985 |
| Right | Putamen | -0.061 | -0.11;-0.01 | -0.08 | 0.03 | -0.14;-0.01 | **0.0163** | 875 | 3994 |
| Left | Thalamus | -0.120 | -0.17;-0.07 | -0.16 | 0.03 | -0.23;-0.09 | **<0.0001** | 864 | 4014 |
| Right | Thalamus | -0.120 | -0.17;-0.07 | -0.17 | 0.04 | -0.24;-0.10 | **<0.0001** | 846 | 3972 |
| Left | Ventricles | 0.186 | 0.13;0.24 | 0.31 | 0.04 | 0.22;0.39 | **<0.0001** | 865 | 3283 |
| Right | Ventricles | 0.168 | 0.12;0.22 | 0.28 | 0.04 | 0.19;0.36 | **<0.0001** | 865 | 3283 |

*Note. d* = Cohen’s d, Std. β = Standardised beta coefficient, CI = Confidence Intervals, SE= Standard Error. In each model we controlled for sex, age, ICV (for subcortical structures) and other concurrently used psychotropic medications (lithium, antipsychotics, antidepressants).

# Supplementary Table 17. The association of antipsychotic treatment with subcortical volume – BD patients taking antipsychotics vs. CN across hemispheres

| **Hemisphere** | **Brain Region** | **d** | ***d 95% CI*** | **Std. β** | **Std_Err** | **Std. β 95% CI** | **p-value^^[[6]](#footnote-6)^^** | **# Taking** | **# HC** |
| --- | --- | --- | --- | --- | --- | --- | --- | --- | --- |
| Left | Accumbens | 0.02 | -0.03;0.07 | 0.03 | 0.04 | -0.04;0.10 | 0.3943 | 922 | 3992 |
| Right | Accumbens | -0.02 | -0.07;0.03 | -0.03 | 0.04 | -0.10;0.04 | 0.3582 | 931 | 3982 |
| Left | Amygdala | -0.03 | -0.08;0.02 | -0.04 | 0.04 | -0.12;0.03 | 0.2889 | 929 | 3968 |
| Right | Amygdala | -0.02 | -0.07;0.03 | -0.03 | 0.04 | -0.10;0.04 | 0.4553 | 930 | 3975 |
| Left | Caudate | 0.04 | -0.01;0.09 | 0.06 | 0.04 | -0.02;0.13 | 0.1298 | 931 | 4004 |
| Right | Caudate | 0.07 | 0.02;0.12 | 0.11 | 0.04 | 0.03;0.18 | **0.0042** | 931 | 4009 |
| Left | Globus Pallidus | 0.04 | -0.01;0.10 | 0.06 | 0.03 | -0.01;0.12 | 0.0843 | 902 | 3843 |
| Right | Globus Pallidus | 0.01 | -0.04;0.06 | 0.01 | 0.04 | -0.06;0.08 | 0.7942 | 934 | 3971 |
| Left | Hippocampus | -0.09 | -0.14;-0.04 | -0.15 | 0.04 | -0.23;-0.07 | **0.0002** | 912 | 3971 |
| Right | Hippocampus | -0.07 | -0.12;-0.02 | -0.11 | 0.04 | -0.19;-0.03 | **0.0067** | 922 | 3967 |
| Left | ICV | -0.03 | -0.08;0.02 | -0.04 | 0.04 | -0.12;0.03 | 0.2448 | 949 | 4038 |
| Right | ICV | -0.03 | -0.08;0.02 | -0.04 | 0.04 | -0.12;0.03 | 0.2448 | 949 | 4038 |
| Left | Putamen | 0.05 | 0.00;0.10 | 0.06 | 0.03 | 0.00;0.13 | 0.0676 | 931 | 3985 |
| Right | Putamen | 0.06 | 0.01;0.11 | 0.07 | 0.03 | 0.01;0.14 | **0.0211** | 938 | 3994 |
| Left | Thalamus | -0.05 | -0.10;0.00 | -0.07 | 0.03 | -0.14;0.00 | **0.0458** | 929 | 4014 |
| Right | Thalamus | -0.07 | -0.12;-0.02 | -0.10 | 0.04 | -0.17;-0.03 | **0.0060** | 915 | 3972 |
| Left | Ventricles | 0.11 | 0.06;0.16 | 0.18 | 0.04 | 0.10;0.27 | **<0.0001** | 927 | 3283 |
| Right | Ventricles | 0.11 | 0.06;0.17 | 0.19 | 0.04 | 0.10;0.28 | **<0.0001** | 927 | 3283 |

*Note. d* = Cohen’s d, Std. β = Standardised beta coefficient, CI = Confidence Intervals, SE= Standard Error. In each model we controlled for sex, age, ICV (for subcortical structures) and other concurrently used psychotropic medications (lithium, antiepileptics, antidepressants).

# Supplementary Table 18. The association of antidepressant treatment with subcortical volume – BD patients taking antidepressants vs. CN across hemispheres

| **Hemisphere** | **Brain Region** | **d** | **d 95% CI** | **Std. β** | **SE** | **Std. β 95% CI** | **p-value^^[[7]](#footnote-7)^^** | **# Taking** | **# HC** |
| --- | --- | --- | --- | --- | --- | --- | --- | --- | --- |
| Left | Accumbens | 0.03 | -0.02;0.08 | 0.04 | 0.04 | -0.03;0.11 | 0.244 | 771 | 3992 |
| Right | Accumbens | -0.02 | -0.07;0.03 | -0.02 | 0.04 | -0.10;0.05 | 0.490 | 778 | 3982 |
| Left | Amygdala | -0.01 | -0.06;0.04 | -0.02 | 0.04 | -0.10;0.06 | 0.588 | 768 | 3968 |
| Right | Amygdala | -0.04 | -0.09;0.01 | -0.06 | 0.04 | -0.13;0.01 | 0.118 | 768 | 3975 |
| Left | Caudate | 0.00 | -0.05;0.05 | 0.00 | 0.04 | -0.07;0.08 | 0.913 | 774 | 4004 |
| Right | Caudate | 0.02 | -0.02;0.07 | 0.04 | 0.04 | -0.04;0.11 | 0.328 | 777 | 4009 |
| Left | Globus Pallidus | -0.03 | -0.08;0.02 | -0.04 | 0.03 | -0.10;0.03 | 0.272 | 757 | 3843 |
| Right | Globus Pallidus | -0.01 | -0.06;0.04 | -0.01 | 0.04 | -0.09;0.06 | 0.696 | 781 | 3971 |
| Left | Hippocampus | -0.06 | -0.11;-0.01 | -0.10 | 0.04 | -0.18;-0.02 | **0.016** | 757 | 3971 |
| Right | Hippocampus | -0.05 | -0.10;0.00 | -0.08 | 0.04 | -0.16;0.00 | **0.040** | 766 | 3967 |
| Left | ICV | -0.03 | -0.08;0.02 | -0.05 | 0.04 | -0.12;0.03 | 0.216 | 796 | 4038 |
| Right | ICV | -0.03 | -0.08;0.02 | -0.05 | 0.04 | -0.12;0.03 | 0.216 | 796 | 4038 |
| Left | Putamen | 0.04 | -0.01;0.09 | 0.05 | 0.04 | -0.02;0.12 | 0.124 | 776 | 3985 |
| Right | Putamen | 0.03 | -0.02;0.08 | 0.04 | 0.03 | -0.03;0.10 | 0.281 | 785 | 3994 |
| Left | Thalamus | -0.05 | -0.10;0.00 | -0.07 | 0.03 | -0.14;0.00 | **0.043** | 777 | 4014 |
| Right | Thalamus | -0.03 | -0.08;0.02 | -0.05 | 0.04 | -0.12;0.02 | 0.171 | 757 | 3972 |
| Left | Ventricles | 0.05 | -0.01;0.10 | 0.08 | 0.05 | -0.01;0.17 | 0.087 | 757 | 3283 |
| Right | Ventricles | 0.04 | -0.01;0.09 | 0.07 | 0.05 | -0.02;0.16 | 0.120 | 757 | 3283 |

*Note*. *d* = Cohen’s d, Std. β = Standardised beta coefficient, CI = Confidence Intervals, SE= Standard Error. In each model we controlled for sex, age, ICV (for subcortical structures) and other concurrently used psychotropic medications (lithium, antiepileptics, antipsychotics).

# Supplementary Table 19. The association between lithium serum levels and subcortical volume in BD

| **ROI** | **N** | **Beta** | **SE** | **df** | **t** | **p** |
| --- | --- | --- | --- | --- | --- | --- |
| Hippocampus | 118 | 0.13304 | 0.36345 | 111 | 0.36603 | 0.71504 |
| Thalamus | 123 | 0.20344 | 0.33629 | 117.7 | 0.60498 | 0.54636 |
| Amygdala | 116 | 0.16803 | 0.27146 | 110.7 | 0.61899 | 0.5372 |
| Caudate | 117 | -0.54496 | 0.28962 | 111.2 | -1.88163 | 0.0625 |
| Putamen | 123 | 0.13284 | 0.27282 | 117.2 | 0.48691 | 0.62723 |
| Pallidus | 124 | 0.18876 | 0.30462 | 118.2 | 0.61965 | 0.53668 |
| Accumbens | 124 | 0.01952 | 0.24781 | 119 | 0.07876 | 0.93735 |
| Ventricles | 126 | -0.55276 | 0.40633 | 120.8 | -1.36037 | 0.17624 |

# Supplementary Table 20. The association of lithium treatment with subcortical volume – BD patients taking lithium vs. BD patients not taking lithium

|  | ***d*** | ***d* 95% CI** | **Std. β** | **Std. β 95% CI** | **SE** | **p-value** | **q-value** | **# Taking** | **# Not Taking** |
| --- | --- | --- | --- | --- | --- | --- | --- | --- | --- |
| Ventricles | -0.04 | [-.12, .04] | -0.02 | [-.06, .02] | 0.04 | 0.32 | 0.34 | 846 | 1493 |
| Hippocampus | 0.12 | [.04, .20] | 0.05 | [.02, .08] | 0.04 | 0.0040 | **0.019** | 840 | 1491 |
| Thalamus | 0.18 | [.10, .26] | 0.07 | [.04, .10] | 0.03 | <0.0001 | **0.0002** | 847 | 1509 |
| Caudate | -0.06 | [-.15, .02] | -0.03 | [-.06, .01] | 0.04 | 0.12 | 0.202 | 841 | 1510 |
| Putamen | -0.01 | [-.09, .07] | 0.00 | [-.03, .03] | 0.03 | 0.86 | 0.56 | 851 | 1512 |
| Globus Pallidus | -0.03 | [-.11, .05] | -0.01 | [-.04, .02] | 0.03 | 0.43 | 0.40 | 851 | 1510 |
| Amygdala | 0.06 | [-.02, .14] | 0.02 | [-.01, .05] | 0.03 | 0.17 | 0.25 | 842 | 1504 |
| Accumbens | -0.02 | [-.10, .06] | -0.01 | [-.04, .02] | 0.03 | 0.60 | 0.48 | 849 | 1499 |
| ICV | 0.02 | [-.06, .10] | 0.01 | [-.02, .04] | 0.04 | 0.61 | 0.48 | 856 | 1522 |

*Note*. d = Cohen’s d, Std. β = Standardized beta coefficient, CI = Confidence Intervals, SE= Standard Error. In each model we controlled for sex, age, ICV (for subcortical structures) and other concurrently used psychotropic medications (antiepileptics, antipsychotics, antidepressants).

# Supplementary Table 21. The association of antiepileptic treatment with subcortical volume – BD patients taking antiepileptics vs. BD patients not taking antiepileptics

|  | ***d*** | ***d* 95% CI** | **Std. β** | **Std. β 95% CI** | **SE** | **p-value** | **q-value** | **# Taking** | **# Not Taking** |
| --- | --- | --- | --- | --- | --- | --- | --- | --- | --- |
| Ventricles | 0.16 | [.08, .24] | 0.07 | [.04, .11] | 0.04 | <0.0001 | **0.0009** | 897 | 1442 |
| Hippocampus | -0.11 | [-.19, -.03] | -0.04 | [-.08, -.01] | 0.04 | 0.0098 | **0.037** | 898 | 1433 |
| Thalamus | -0.21 | [-.30, -.13] | -0.08 | [-.11, -.05] | 0.03 | <0.0001 | **<0.0001** | 910 | 1446 |
| Caudate | -0.11 | [-.19, -.03] | -0.04 | [-.07, -.01] | 0.03 | 0.010 | **0.037** | 907 | 1444 |
| Putamen | -0.18 | [-.26, -.10] | -0.06 | [-.09, -.03] | 0.03 | <0.0001 | **0.0002** | 911 | 1452 |
| Globus Pallidus | -0.06 | [-.14, .02] | -0.02 | [-.05, .01] | 0.03 | 0.16 | 0.25 | 913 | 1448 |
| Amygdala | -0.07 | [-.15, .01] | -0.03 | [-.05, .00] | 0.03 | 0.076 | 0.15 | 907 | 1439 |
| Accumbens | -0.10 | [-.18, -.02] | -0.03 | [-.06, -.01] | 0.03 | 0.018 | 0.051 | 903 | 1445 |
| ICV | 0.00 | [-.08, .08] | 0.00 | [-.03, .03] | 0.03 | 0.94 | 0.58 | 915 | 1463 |

*Note. d =* Cohen’s d, Std. β = Standardized beta coefficient, CI = Confidence Intervals, SE= Standard Error. In each model we controlled for sex, age, ICV (for subcortical structures) and other concurrently used psychotropic medications (lithium, antipsychotics, antidepressants).

# Supplementary Table 22. The association of antipsychotic treatment with subcortical volume – BD patients taking antipsychotics vs. BD patients not taking antipsychotics

|  | ***d*** | ***d* 95% CI** | **Std. β** | **Std. β 95% CI** | **SE** | **p-value** | **q-value** | **# Taking** | **# Not Taking** |
| --- | --- | --- | --- | --- | --- | --- | --- | --- | --- |
| Ventricles | 0.06 | [-.02, .14] | 0.03 | [-.01, .06] | 0.04 | 0.17 | 0.25 | 971 | 1368 |
| Hippocampus | -0.08 | [-.16, .00] | -0.03 | [-.07, .00] | 0.04 | 0.065 | 0.13 | 981 | 1350 |
| Thalamus | -0.10 | [-.19, -.02] | -0.04 | [-.07, -.01] | 0.03 | 0.012 | **0.041** | 983 | 1373 |
| Caudate | 0.06 | [-.02, .14] | 0.02 | [-.01, .06] | 0.03 | 0.17 | 0.25 | 980 | 1371 |
| Putamen | 0.04 | [-.04, .12] | 0.01 | [-.01, .04] | 0.03 | 0.33 | 0.35 | 986 | 1377 |
| Globus Pallidus | 0.06 | [-.02, .14] | 0.02 | [-.01, .05] | 0.03 | 0.16 | 0.25 | 986 | 1375 |
| Amygdala | -0.05 | [-.13, .03] | -0.02 | [-.05, .01] | 0.03 | 0.26 | 0.30 | 979 | 1367 |
| Accumbens | -0.02 | [-.11, .06] | -0.01 | [-.04, .02] | 0.03 | 0.55 | 0.46 | 980 | 1368 |
| ICV | 0.04 | [-.04, .12] | 0.02 | [-.02, .05] | 0.03 | 0.29 | 0.32 | 993 | 1385 |

*Note. d* = Cohen’s d, Std. β = Standardized beta coefficient, CI = Confidence Intervals, SE= Standard Error. In each model we controlled for sex, age, ICV (for subcortical structures) and other concurrently used psychotropic medications (lithium, antiepileptics, antidepressants).

# Supplementary Table 23. The association of antidepressant treatment with subcortical volume – BD patients taking antidepressants vs. BD patients not taking antidepressants

|  | ***d*** | ***d* 95% CI** | **Std. β** | **Std. β 95% CI** | **SE** | **p-value** | **q-value** | **# Taking** | **# Not Taking** |
| --- | --- | --- | --- | --- | --- | --- | --- | --- | --- |
| Ventricles | -0.05 | [-.13, .03] | -0.02 | [-.06, .01] | 0.04 | 0.22 | 0.28 | 777 | 1562 |
| Hippocampus | -0.02 | [-.10, .06] | -0.01 | [-.04, .03] | 0.04 | 0.62 | 0.49 | 797 | 1534 |
| Thalamus | -0.01 | [-.10, .07] | -0.01 | [-.04, .03] | 0.03 | 0.73 | 0.52 | 807 | 1549 |
| Caudate | 0.02 | [-.06, .10] | 0.01 | [-.02, .04] | 0.04 | 0.65 | 0.49 | 802 | 1549 |
| Putamen | 0.03 | [-.05, .11] | 0.01 | [-.02, .04] | 0.03 | 0.49 | 0.44 | 811 | 1552 |
| Globus Pallidus | 0.00 | [-.08, .08] | 0.00 | [-.03, .03] | 0.03 | 0.95 | 0.59 | 809 | 1552 |
| Amygdala | -0.02 | [-.10, .06] | -0.01 | [-.04, .02] | 0.03 | 0.66 | 0.50 | 799 | 1547 |
| Accumbens | 0.03 | [-.05, .11] | 0.01 | [-.02, .04] | 0.03 | 0.51 | 0.44 | 805 | 1543 |
| ICV | 0.06 | [-.02, .14] | 0.02 | [-.01, .06] | 0.04 | 0.16 | 0.25 | 816 | 1562 |

*Note. d* = Cohen’s d, Std. β = Standardized beta coefficient, CI = Confidence Intervals, SE= Standard Error. In each model we controlled for sex, age, ICV (for subcortical structures) and other concurrently used psychotropic medications (lithium, antiepileptics, antipsychotics).

# Supplementary Table 24. The association of valproate treatment with subcortical volume – BD patients taking valproate vs. CN

|  | ***d*** | ***d 95% CI*** | **Std. β** | **Std. β 95% CI** | **SE** | **p-value** | **q-value** | **# Taking** | **# CN** |
| --- | --- | --- | --- | --- | --- | --- | --- | --- | --- |
| Ventricles | 0.22 | [.16, .27] | 0.52 | [.38, .66] | 0.07 | <0.0001 | **<0.0001** | 256 | 3284 |
| Hippocampus | -0.10 | [-.16, -.05] | -0.24 | [-.36, -.11] | 0.06 | 0.0002 | **0.0018** | 248 | 4019 |
| Thalamus | -0.18 | [-.23, -.12] | -0.37 | [-.48, -.26] | 0.06 | <0.0001 | **<0.0001** | 254 | 4026 |
| Caudate | -0.03 | [-.09, .02] | -0.08 | [-.20, .04] | 0.06 | 0.21 | 0.28 | 252 | 4022 |
| Putamen | -0.09 | [-.15, -.04] | -0.18 | [-.28, -.07] | 0.05 | 0.0008 | **0.0048** | 255 | 4021 |
| Globus Pallidus | -0.02 | [-.08, .03] | -0.04 | [-.15, .07] | 0.06 | 0.45 | 0.42 | 254 | 4005 |
| Amygdala | -0.04 | [-.10, .01] | -0.09 | [-.21, .03] | 0.06 | 0.13 | 0.22 | 252 | 3998 |
| Accumbens | -0.03 | [-.09, .02] | -0.06 | [-.17, .04] | 0.06 | 0.25 | 0.29 | 251 | 4026 |
| ICV | -0.05 | [-.11, .00] | -0.12 | [-.24, .00] | 0.06 | 0.058 | 0.13 | 256 | 4038 |

*Note. d* = Cohen’s d, Std. β = Standardized beta coefficient, CI = Confidence Intervals, SE= Standard Error. In each model we controlled for sex, age, ICV (for subcortical structures) and other concurrently used NbN classified psychotropic medications.

# Supplementary Table 25. The association of glutamate sodium/calcium channel-blockers (GSCCB) with subcortical volume – BD patients taking glutamate sodium calcium channel-blockers vs. CN

|  | ***d*** | ***d 95% CI*** | **Std. β** | **Std. β 95% CI** | **SE** | **p-value** | **q-value** | **# Taking** | **# CN** |
| --- | --- | --- | --- | --- | --- | --- | --- | --- | --- |
| Ventricles | 0.06 | [.01, .12] | 0.15 | [.01, .28] | 0.06 | 0.031 | 0.085 | 302 | 3284 |
| Hippocampus | -0.05 | [-.11, .00] | -0.11 | [-.23, .01] | 0.06 | 0.065 | 0.13 | 292 | 4019 |
| Thalamus | -0.03 | [-.08, .02] | -0.06 | [-.16, .05] | 0.05 | 0.29 | 0.32 | 299 | 4026 |
| Caudate | -0.01 | [-.07, .04] | -0.03 | [-.14, .09] | 0.06 | 0.65 | 0.49 | 299 | 4022 |
| Putamen | -0.06 | [-.11, .00] | -0.10 | [-.20, -.01] | 0.05 | 0.035 | 0.095 | 299 | 4021 |
| Globus Pallidus | -0.01 | [-.06, .04] | -0.02 | [-.12, .08] | 0.05 | 0.74 | 0.53 | 302 | 4005 |
| Amygdala | 0.00 | [-.05, .06] | 0.01 | [-.10, .12] | 0.05 | 0.91 | 0.57 | 299 | 3998 |
| Accumbens | -0.02 | [-.07, .04] | -0.03 | [-.13, .07] | 0.05 | 0.58 | 0.47 | 297 | 4026 |
| ICV | -0.02 | [-.08, .03] | -0.05 | [-.16, .06] | 0.06 | 0.40 | 0.39 | 302 | 4038 |

*Note. d* = Cohen’s d, Std. β = Standardized beta coefficient, CI = Confidence Intervals, SE= Standard Error. In each model we controlled for sex, age, ICV (for subcortical structures) and other concurrently used NbN classified psychotropic medications.

# Supplementary Table 26. The association of GABA positive allosteric modulators (GABA PAM) with subcortical volume – BD patients taking GABA positive allosteric modulators vs. CN

|  | ***d*** | ***d 95% CI*** | **Std. β** | **Std. β 95% CI** | **SE** | **p-value** | **q-value** | **# Taking** | **# CN** |
| --- | --- | --- | --- | --- | --- | --- | --- | --- | --- |
| Ventricles | 0.11 | [.05, .16] | 0.27 | [.12, .42] | 0.07 | **0.0004** | 0.0029 | 296 | 3284 |
| Hippocampus | -0.06 | [-.11, .00] | -0.14 | [-.27, .00] | 0.07 | **0.044** | 0.11 | 282 | 4019 |
| Thalamus | -0.05 | [-.11, .00] | -0.12 | [-.24, .00] | 0.06 | 0.056 | 0.13 | 289 | 4026 |
| Caudate | 0.06 | [.01, .12] | 0.15 | [.02, .28] | 0.07 | **0.02** | 0.06 | 287 | 4022 |
| Putamen | -0.01 | [-.07, .04] | -0.02 | [-.13, .09] | 0.06 | 0.69 | 0.51 | 292 | 4021 |
| Globus Pallidus | 0.04 | [-.01, .09] | 0.08 | [-.03, .20] | 0.06 | 0.15 | 0.24 | 295 | 4005 |
| Amygdala | 0.00 | [-.06, .05] | 0.00 | [-.13, .12] | 0.06 | 0.97 | 0.59 | 284 | 3998 |
| Accumbens | 0.01 | [-.05, .06] | 0.01 | [-.10, .13] | 0.06 | 0.84 | 0.55 | 289 | 4026 |
| ICV | -0.01 | [-.07, .04] | -0.03 | [-.16, .09] | 0.06 | 0.60 | 0.48 | 296 | 4038 |

*Note. d* = Cohen’s d, Std. β = Standardized beta coefficient, CI = Confidence Intervals, SE= Standard Error. In each model we controlled for sex, age, ICV (for subcortical structures) and other concurrently used NbN classified psychotropic medications

# Supplementary Table 27. The association of primarily dopamine receptor antagonists with subcortical volume – BD patients taking primarily dopamine antagonists vs. CN

|  | ***d*** | ***d 95% CI*** | **Std. β** | **Std. β 95% CI** | **SE** | **p-value** | **q-value** | **# Taking** | **# CN** |
| --- | --- | --- | --- | --- | --- | --- | --- | --- | --- |
| Ventricles | 0.03 | [-.02, .09] | 0.13 | [-.09, .35] | 0.11 | 0.26 | 0.30 | 77 | 3284 |
| Hippocampus | 0.02 | [-.03, .08] | 0.09 | [-.11, .28] | 0.10 | 0.38 | 0.38 | 76 | 4019 |
| Thalamus | 0.03 | [-.02, .09] | 0.11 | [-.07, .29] | 0.09 | 0.22 | 0.28 | 75 | 4026 |
| Caudate | 0.05 | [.00, .10] | 0.18 | [.00, .37] | 0.10 | 0.06 | 0.13 | 75 | 4022 |
| Putamen | 0.03 | [-.03, .08] | 0.08 | [-.08, .24] | 0.08 | 0.34 | 0.35 | 77 | 4021 |
| Globus Pallidus | -0.01 | [-.07, .04] | -0.04 | [-.21, .12] | 0.09 | 0.61 | 0.48 | 76 | 4005 |
| Amygdala | 0.03 | [-.03, .08] | 0.09 | [-.10, .27] | 0.09 | 0.35 | 0.36 | 73 | 3998 |
| Accumbens | 0.02 | [-.03, .08] | 0.07 | [-.10, .24] | 0.09 | 0.44 | 0.41 | 74 | 4026 |
| ICV | 0.01 | [-.05, .06] | 0.03 | [-.16, .22] | 0.09 | 0.75 | 0.53 | 77 | 4038 |

*Note. d* = Cohen’s d, Std. β = Standardized beta coefficient, CI = Confidence Intervals, SE= Standard Error. In each model we controlled for sex, age, ICV (for subcortical structures) and other concurrently used NbN classified psychotropic medications.

# Supplementary Table 28. The association of dopamine and other monoamine receptor antagonists with subcortical volume – BD patients taking dopamine and other receptor antagonists vs. CN

|  | ***d*** | ***d 95% CI*** | **Std. β** | **Std. β 95% CI** | **SE** | **p-value** | **q-value** | **# Taking** | **# CN** |
| --- | --- | --- | --- | --- | --- | --- | --- | --- | --- |
| Ventricles | 0.08 | [.02, .13] | 0.15 | [.04, .27] | 0.06 | 0.011 | **0.041** | 480 | 3284 |
| Hippocampus | -0.09 | [-.14, -.03] | -0.17 | [-.27, -.06] | 0.05 | 0.0017 | **0.009** | 474 | 4019 |
| Thalamus | -0.05 | [-.10, .01] | -0.08 | [-.18, .01] | 0.05 | 0.093 | 0.17 | 476 | 4026 |
| Caudate | 0.04 | [-.02, .09] | 0.07 | [-.03, .17] | 0.05 | 0.20 | 0.26 | 474 | 4022 |
| Putamen | 0.02 | [-.04, .07] | 0.03 | [-.06, .12] | 0.04 | 0.53 | 0.46 | 476 | 4021 |
| Globus Pallidus | -0.01 | [-.06, .05] | -0.01 | [-.10, .08] | 0.05 | 0.80 | 0.54 | 479 | 4005 |
| Amygdala | -0.05 | [-.10, .01] | -0.08 | [-.18, .01] | 0.05 | 0.094 | 0.17 | 474 | 3998 |
| Accumbens | 0.01 | [-.05, .06] | 0.01 | [-.08, .10] | 0.05 | 0.82 | 0.55 | 475 | 4026 |
| ICV | -0.06 | [-.11, .00] | -0.11 | [-.21, -.01] | 0.05 | 0.037 | 0.095 | 480 | 4038 |

*Note. d* = Cohen’s d, Std. β = Standardized beta coefficient, CI = Confidence Intervals, SE= Standard Error. In each model we controlled for sex, age, ICV (for subcortical structures) and other concurrently used NbN classified psychotropic medications.

# Supplementary Table 29. The association of dopamine-serotonin partial agonists and antagonists with subcortical volume – BD patients taking dopamine partial agonists vs. CN

|  | ***d*** | ***d 95% CI*** | **Std. β** | **Std. β 95% CI** | **SE** | **p-value** | **q-value** | **# Taking** | **# CN** |
| --- | --- | --- | --- | --- | --- | --- | --- | --- | --- |
| Ventricles | 0.00 | [-.06, .06] | -0.01 | [-.29, .27] | 0.14 | 0.95 | 0.58 | 40 | 3284 |
| Hippocampus | 0.01 | [-.04, .06] | 0.05 | [-.20, .30] | 0.13 | 0.70 | 0.51 | 39 | 4019 |
| Thalamus | -0.01 | [-.06, .05] | -0.02 | [-.25, .20] | 0.12 | 0.83 | 0.55 | 39 | 4026 |
| Caudate | 0.09 | [.03, .14] | 0.39 | [.15, .64] | 0.12 | 0.0015 | **0.008** | 38 | 4022 |
| Putamen | 0.08 | [.02, .13] | 0.29 | [.08, .50] | 0.11 | 0.0061 | **0.027** | 39 | 4021 |
| Globus Pallidus | 0.07 | [.01, .12] | 0.28 | [.06, .50] | 0.11 | 0.012 | **0.041** | 38 | 4005 |
| Amygdala | 0.03 | [-.02, .08] | 0.13 | [-.10, .37] | 0.12 | 0.28 | 0.31 | 38 | 3998 |
| Accumbens | 0.06 | [.00, .11] | 0.23 | [.01, .45] | 0.11 | 0.037 | 0.10 | 38 | 4026 |
| ICV | -0.04 | [-.09, .02] | -0.17 | [-.41, .08] | 0.12 | 0.18 | 0.26 | 40 | 4038 |

*Note. d* = Cohen’s d, Std. β = Standardized beta coefficient, CI = Confidence Intervals, SE= Standard Error. In each model we controlled for sex, age, ICV (for subcortical structures) and other concurrently used NbN classified psychotropic medications.

# Supplementary Table 30. The association of drugs targeting serotonin with subcortical volume – BD patients taking drugs targeting serotonin vs. CN

|  | ***d*** | ***d 95% CI*** | **Std. β** | **Std. β 95% CI** | **SE** | **p-value** | **q-value** | **# Taking** | **# CN** |
| --- | --- | --- | --- | --- | --- | --- | --- | --- | --- |
| Ventricles | 0.04 | [-.02, .10] | 0.09 | [-.05, .24] | 0.07 | 0.20 | 0.26 | 243 | 3284 |
| Hippocampus | -0.03 | [-.09, .02] | -0.08 | [-.20, .05] | 0.06 | 0.24 | 0.29 | 234 | 4019 |
| Thalamus | -0.02 | [-.07, .04] | -0.03 | [-.15, .08] | 0.06 | 0.56 | 0.46 | 239 | 4026 |
| Caudate | 0.04 | [-.01, .09] | 0.09 | [-.03, .21] | 0.06 | 0.15 | 0.24 | 237 | 4022 |
| Putamen | -0.01 | [-.06, .05] | -0.01 | [-.12, .09] | 0.05 | 0.79 | 0.54 | 241 | 4021 |
| Globus Pallidus | 0.01 | [-.05, .06] | 0.01 | [-.09, .12] | 0.06 | 0.79 | 0.54 | 240 | 4005 |
| Amygdala | -0.04 | [-.09, .02] | -0.08 | [-.20, .04] | 0.06 | 0.19 | 0.26 | 236 | 3998 |
| Accumbens | 0.01 | [-.05, .06] | 0.02 | [-.09, .13] | 0.06 | 0.76 | 0.53 | 237 | 4026 |
| ICV | -0.10 | [-.15, -.04] | -0.22 | [-.34, -.09] | 0.06 | 0.0005 | **0.0034** | 243 | 4038 |

*Note. d* = Cohen’s d, Std. β = Standardized beta coefficient, CI = Confidence Intervals, SE= Standard Error. In each model we controlled for sex, age, ICV (for subcortical structures) and other concurrently used NbN classified psychotropic medications.

# Supplementary Table 31. The association of drugs targeting serotonin and other monoamines with subcortical volume – BD patients taking drugs targeting serotonin and other vs. CN

|  | ***d*** | ***d 95% CI*** | **Std. β** | **Std. β 95% CI** | **SE** | **p-value** | **q-value** | **# Taking** | **# CN** |
| --- | --- | --- | --- | --- | --- | --- | --- | --- | --- |
| Ventricles | 0.04 | [-.02, .10] | 0.09 | [-.06, .24] | 0.07 | 0.22 | 0.28 | 238 | 3284 |
| Hippocampus | -0.04 | [-.09, .02] | -0.09 | [-.22, .05] | 0.07 | 0.20 | 0.26 | 232 | 4019 |
| Thalamus | -0.04 | [-.09, .02] | -0.08 | [-.20, .04] | 0.06 | 0.17 | 0.25 | 235 | 4026 |
| Caudate | 0.05 | [-.01, .10] | 0.11 | [-.02, .23] | 0.07 | 0.10 | 0.17 | 234 | 4022 |
| Putamen | 0.01 | [-.05, .06] | 0.02 | [-.09, .13] | 0.06 | 0.76 | 0.53 | 236 | 4021 |
| Globus Pallidus | 0.02 | [-.04, .07] | 0.04 | [-.07, .15] | 0.06 | 0.50 | 0.44 | 237 | 4005 |
| Amygdala | -0.01 | [-.07, .04] | -0.03 | [-.16, .09] | 0.06 | 0.61 | 0.48 | 230 | 3998 |
| Accumbens | 0.05 | [.00, .11] | 0.11 | [.00, .23] | 0.06 | 0.048 | 0.12 | 237 | 4026 |
| ICV | 0.02 | [-.04, .07] | 0.04 | [-.08, .17] | 0.06 | 0.50 | 0.44 | 238 | 4038 |

*Note. d* = Cohen’s d, Std. β = Standardized beta coefficient, CI = Confidence Intervals, SE= Standard Error. In each model we controlled for sex, age, ICV (for subcortical structures) and other concurrently used NbN classified psychotropic medications.

# Table 32. Demographic and Clinical Comparisons in NbN subset sample

| **Syndrome Classification** |  | **AED** |  |  | **AP** |  |  |  | **AD** |  | | **Benzo** | | |
| --- | --- | --- | --- | --- | --- | --- | --- | --- | --- | --- | --- | --- | --- | --- |
| **NbN Classification** | **Patients taking Valproate (n=262)** | **Patients taking GSCCB (n=304)** |  | **Patients taking Primarily Dopa. (n=79)** | **Patients taking Dopa. And Other (n=484)** | **Patients taking Partial Dopa. Agonists (n=40)** |  | **Patients taking Targeting Sero. (n=247)** | **Patients taking Targeting Sero. And Other (n=242)** | |  | | **Patients taking GABA PAM (n=308)** |  |
| ***Demographic and clinical information*** | ***M(SD)/%*** | ***M(SD)/%*** |  | ***M(SD)/%*** | ***M(SD)/%*** | ***M(SD)/%*** |  | ***M(SD)/%*** | ***M(SD)/%*** | |  | | ***M(SD)/%*** |  |
| Age at Time of Scan | 43 (12.1) | 41.4 (11.6) |  | 44.8 (11.2) | 39.3 (12.3) | 37.5 (12.2) |  | 42.1 (11.8) | 46.4 (11.2) | |  | | 46.1 (11) |  |
| Gender | 138 (53%) female | 204 (67%) female |  | 43 (54%) female | 271 (56%) female | 22 (55%) female |  | 165 (67%) female | 143 (59%) female | |  | | 207 (67%) female |  |
| BDI/BDII/BDNOS | 182/22/1/57 | 183/68/2/51 |  | 53/8/1/17 | 359/36/1/88 | 26/2/0/12 |  | 153/62/1/31 | 134/54/5/49 | |  | | 204/63/1/40 |  |
| Body Mass Index (BMI) | 27.7 (6.3) | 27.1 (5.7) |  | 27.3 (5.9) | 27.2 (5.3) | 30.6 (4.6) |  | 26.3 (5.3) | 27.9 (6.2) | |  | | 26.5 (5.4) |  |
| Education in Years | 14.1 (3.7) | 14.3 (3.4) |  | 14 (3.6) | 14.5 (3.6) | 14.7 (3.3) |  | 14.4 (3.7) | 14 (3.5) | |  | | 13.7 (4) |  |
| Full Scale IQ | 94.3 (22.2) |  |  | 102.5 (18.9) | 104.7 (18.8) | 105.9 (24.6) |  | 108.2 (19.8) | 109.7 (18.1) | |  | | 100.8 (18.5) |  |
| CTQ Total | 44.4 (14.3) | 36.6 (22.1) |  | 49.4 (26.4) | 44.3 (16.7) | 48.3 (16.3) |  | 39.7 (18.2) | 43 (18.2) | |  | | 40.4 (15.4) |  |
| ***Illness Course Measures*** |  |  |  |  |  |  |  |  |  | |  | |  |  |
| History of Psychosis | 93 (35.5%) | 87 (28.6%) |  | 33 (41.2%) | 150 (31%) | 14 (35%) |  | 61 (24.7%) | 59 (24.4%) | |  | | 101 (32.8%) |  |
| Illness Duration (in years) | 17.6 (11.5) | 17 (10.7) |  | 16.9 (10.9) | 15.5 (11.1) | 15.8 (11.1) |  | 17.1 (10.8) | 19.4 (11.4) | |  | | 18 (11) |  |
| Age of Onset (in years) | 25.9 (10.9) | 24 (10) |  | 28.7 (11.5) | 24.7 (10) | 23.3 (8.2) |  | 25 (10.8) | 26.6 (10.9) | |  | | 28.1 (10.9) |  |
| Psychiatric Hospitalizations | 5.2 (5) | 2.9 (6.7) |  | 4.32 (3.2) | 4.7 (5.4) | 5.9 (6.4) |  | 4.3 (5.1) | 4.8 (4.6) | |  | | 5.7 (7.5) |  |
| Total Hypomanic Episodes | 1.1 (5.8) | 7.8 (17.2) |  | 0.2 (0.4) | 2.3 (10) | 0.2 (0.4) |  | 3.1 (10.2) | 4.5 (11.4) | |  | | 3.3 (13.1) |  |
| Total Manic Episodes | 7.7 (17.2) | 10.1 (23.1) |  | 4 (7.2) | 8.3 (20.9) | 3.6 (3) |  | 8 (20.8) | 5.2 (14) | |  | | 6.9 (19.3) |  |
| Total Depressive Episodes | 9.5 (16.5) | 15.6 (23.5) |  | 5.3 (5.6) | 9.7 (18.5) | 7.9 (12.6) |  | 13.8 (22.5) | 11.5 (16.1) | |  | | 10.4 (17.5) |  |
| Total Mixed Episodes | 1 (4) | 0.4 (1.6) |  | 0.1 (0.3) | 1.1 (4.2) | - |  | 0.5 (2.6) | 1 (13.3) | |  | | 0.3 (1.9) |  |
| BDI Total | 15.1 (13.3) | 11 (12.2) |  | 6.1 (6) | 9.9 (10.8) | 8.8 (7.9) |  | 13.9 (14.2) | 17.9 (13.1) | |  | | 12 (14.3) |  |
| HDRS Total | 15.4 (9.6) | 11.1 (9.4) |  | 13 (9.7) | 8.5 (8.3) | 7.5 (6.1) |  | 14.9 (9.3) | 14.2 (8.5) | |  | | 16.7 (9) |  |
| MADRS Total | 8.64 (8.1) | 10.9 (10.1) |  | 7.4 (9.3) | 8.7 (7.7) | 9.3 (7.8) |  | 11.3 (8.9) | 14.3 (9.5) | |  | | 11.4 (9.6) |  |
| CES-D Total | 24.4 (13.9) | 26.2 (14.6) |  | - | 23.3 (12.2) | - |  |  | - | |  | | 27.9 (14.3) |  |

*Note.* z = Mann-Whitney *z*-value, χ² = Chi-Square statistic. Li = Lithium, AED = Antiepileptics, AP = Antipsychotics, AD = Antidepressants, BMI = Body Mass Index, CTQ = Childhood Trauma Questionnaire, BDI = Beck's Depression inventory, HDRS = Hamilton Depression Rating Scale, MADRS = Montgomery-Asberg Depression Rating Scale, CES-D = Center for Epidemiological Studies Depression. GSCCB = Glutamate sodium/calcium channel blockers, GABA PAM = GABA positive allosteric modulators.

# Supplementary Table 33. The association of traditional syndrome-based medication with subcortical volume – BD patients taking vs. CN of subset sample included in NbN analysis

| **Brain Region** | **d** | **d 95% CI** | **Std. β** | **SE** | **Std. β 95% CI** | **p-value^^[[8]](#footnote-8)^^** | **# Taking** | **# HC** |
| --- | --- | --- | --- | --- | --- | --- | --- | --- |
| ***AED*** |  |  |  |  |  |  |  |  |
| Ventricles | 0.1835416 | 0.12;0.24 | 0.36632681 | 0.06 | 0.25;0.48 | **<0.0001** | 535 | 3284 |
| Caudate | -0.01177213 | -0.07;0.04 | -0.02131993 | 0.05 | -0.12;0.08 | 0.6721 | 527 | 4022 |
| Putamen | -0.07866105 | -0.13;-0.02 | -0.12343487 | 0.04 | -0.21;-0.04 | **0.0047** | 531 | 4021 |
| Globus Pallidus | 0.01438332 | -0.04;0.07 | 0.02344628 | 0.05 | -0.07;0.11 | 0.6053 | 533 | 4005 |
| Hippocampus | -0.07909167 | -0.13;-0.02 | -0.15017266 | 0.05 | -0.25;-0.05 | **0.0046** | 518 | 4019 |
| Amygdala | -0.02504505 | -0.08;0.03 | -0.04402814 | 0.05 | -0.14;0.05 | 0.3693 | 527 | 3998 |
| Accumbens | -0.05547866 | -0.11;0.00 | -0.09026293 | 0.05 | -0.18;0.00 | **0.0460** | 529 | 4026 |
| Thalamus | -0.12261713 | -0.18;-0.07 | -0.21047411 | 0.05 | -0.30;-0.12 | **<0.0001** | 530 | 4026 |
| ICV | -0.03859288 | -0.09;0.02 | -0.07112974 | 0.05 | -0.17;0.03 | 0.1638 | 535 | 4038 |
| ***AP*** |  |  |  |  |  |  |  |  |
| Ventricles | 0.099741 | 0.04;0.16 | 0.20695374 | 0.06 | 0.09;0.33 | **0.0009** | 529 | 3284 |
| Caudate | 0.06479641 | 0.01;0.12 | 0.12100893 | 0.05 | 0.02;0.22 | **0.0199** | 519 | 4022 |
| Putamen | 0.02358728 | -0.03;0.08 | 0.03822062 | 0.05 | -0.05;0.13 | 0.3960 | 524 | 4021 |
| Globus Pallidus | 0.01099316 | -0.04;0.07 | 0.0184947 | 0.05 | -0.07;0.11 | 0.6928 | 525 | 4005 |
| Hippocampus | -0.07367426 | -0.13;-0.02 | -0.14315562 | 0.05 | -0.25;-0.04 | **0.0082** | 519 | 4019 |
| Amygdala | -0.02925566 | -0.08;0.03 | -0.05304035 | 0.05 | -0.15;0.05 | 0.2944 | 517 | 3998 |
| Accumbens | 0.0309463 | -0.02;0.09 | 0.05207893 | 0.05 | -0.04;0.14 | 0.2655 | 521 | 4026 |
| Thalamus | -0.03970861 | -0.09;0.01 | -0.07039734 | 0.05 | -0.17;0.03 | 0.1532 | 521 | 4026 |
| ICV | -0.05984479 | -0.11;-0.01 | -0.11414168 | 0.05 | -0.22;-0.01 | **0.0309** | 529 | 4038 |
| ***AD*** |  |  |  |  |  |  |  |  |
| Ventricles | 0.060999124 | 0.00;0.12 | 0.129 | 0.06 | 0.00;0.25 | **0.04188** | 462 | 3284 |
| Hippocampus | -0.052740615 | -0.11;0.00 | -0.105 | 0.06 | -0.21;0.00 | **..** | 444 | 4019 |
| Caudate | 0.049476619 | -0.01;0.10 | 0.095 | 0.05 | -0.01;0.20 | 0.07534 | 449 | 4022 |
| Amygdala | -0.039686254 | -0.09;0.01 | -0.074 | 0.05 | -0.18;0.03 | 0.15490 | 446 | 3998 |
| Thalamus | -0.037581454 | -0.09;0.02 | -0.068 | 0.05 | -0.17;0.03 | 0.17639 | 454 | 4026 |
| ICV | -0.036852712 | -0.09;0.02 | -0.072 | 0.05 | -0.18;0.03 | 0.18365 | 462 | 4038 |
| Accumbens | 0.030276892 | -0.02;0.08 | 0.052 | 0.05 | -0.04;0.15 | 0.27601 | 454 | 4026 |
| Putamen | -0.003819633 | -0.06;0.05 | -0.006 | 0.05 | -0.10;0.08 | 0.89068 | 458 | 4021 |
| Globus Pallidus | -0.001288226 | -0.06;0.05 | -0.002 | 0.05 | -0.10;0.09 | 0.96308 | 458 | 4005 |

*Note. d* = Cohen’s d, Std. β = Standardised beta coefficient, CI = Confidence Intervals, SE= Standard Error, ICV= intracranial volume, Li= Lithium, AED= antiepileptics, AP= antipsychotics, AD= antidepressants. Sex, age, and ICV (for subcortical structures) were controlled for in each model.

# Supplementary Table 34. The moderating role of lithium on the associations between antiepileptics and antipsychotics with subcortical volume in BD patients

|  | **β** | **SE** | **t-statistic** | **β 95% CI** | **p-value** | **q-value** |
| --- | --- | --- | --- | --- | --- | --- |
| ***Antiepileptics*** |  |  |  |  |  |  |
| Ventricles | -0.02 | 0.09 | -0.23 | [-.20, .16] | 0.82 | 0.59 |
| Hippocampus | 0.19 | 0.08 | 2.53 | [.04, .34] | **0.012** | 0.038 |
| Thalamus | 0.13 | 0.07 | 1.88 | [.00, .26] | 0.060 | 0.13 |
| Caudate | 0.14 | 0.07 | 1.90 | [.00, .28] | 0.058 | 0.13 |
| Putamen | 0.10 | 0.06 | 1.63 | [-.02, .23] | 0.10 | 0.19 |
| Accumbens | 0.05 | 0.06 | 0.81 | [-.07, .18] | 0.42 | 0.44 |
| ***Antipsychotics*** |  |  |  |  |  |  |
| Thalamus | 0.01 | 0.06 | 0.21 | [-.11, .13] | 0.84 | 0.60 |

*Note.* β = beta coefficient, CI = Confidence Intervals, SE= Standard Error. In each model we controlled for sex, age, ICV (for subcortical structures) and other concurrently used psychotropic medications.

# Supplementary Table 35. Correlation between illness course measures and subcortical volume in BD patients

|  | **Ventricles** | | **Caudate** | | **Accumbens** | | **Amygdala** | | **Putamen** | | **Pallidum** | | **Hippocampus** | | **Thalamus** | | **ICV** | |
| --- | --- | --- | --- | --- | --- | --- | --- | --- | --- | --- | --- | --- | --- | --- | --- | --- | --- | --- |
|  | ***cor*** | ***p*** | ***cor*** | ***p*** | ***cor*** | *p* | ***cor*** | ***p*** | ***cor*** | ***p*** | ***cor*** | ***p*** | *cor* | ***p*** | ***cor*** | ***p*** | ***cor*** | ***p*** |
| Total Manic Episodes | 0.02 | 0.50 | 0.02 | 0.41 | -0.03 | 0.29 | -0.02 | 0.39 | 0.02 | 0.37 | 0.05 | 0.078 | -0.10 | **0.0002** | -0.05 | 0.069 | -0.10 | **0.0004** |
| Total Hypomanic Episodes | 0.00 | 0.99 | 0.05 | 0.16 | 0.08 | 0.011 | 0.12 | **0.0002** | 0.13 | **<0.0001** | 0.05 | 0.12 | -0.06 | 0.054 | -0.09 | **0.010** | -0.02 | 0.56 |
| Total Depressive Episodes | 0.02 | 0.39 | 0.01 | 0.61 | -0.04 | 0.19 | -0.04 | 0.14 | 0.02 | 0.47 | 0.02 | 0.55 | -0.15 | **<0.0001** | -0.11 | **<0.0001** | -0.13 | **<0.0001** |
| No. Psychiatric Hospitalizations | 0.16 | **0.0002** | -0.09 | **0.025** | -0.01 | 0.72 | 0.16 | **0.0001** | -0.12 | **0.0037** | -0.05 | 0.21 | -0.05 | 0.26 | -0.18 | **<0.0001** | 0.11 | **0.0089** |
| Age of Onset | 0.18 | **<0.0001** | -0.23 | **<0.0001** | -0.24 | **<0.0001** | -0.12 | **<0.0001** | -0.25 | **<0.0001** | -0.25 | **<0.0001** | -0.04 | 0.076 | -0.08 | **0.0002** | 0.07 | **0.0008** |
| BMI | 0.17 | **<0.0001** | -0.10 | **<0.0001** | -0.09 | **0.0004** | 0.03 | 0.24 | -0.08 | **0.0026** | -0.10 | **0.0001** | -0.04 | 0.19 | -0.12 | **<0.0001** | -0.04 | 0.15 |
| Illness Duration | 0.19 | **<0.0001** | -0.22 | **<0.0001** | -0.27 | **<0.0001** | -0.15 | **<0.0001** | -0.30 | **<0.0001** | -0.26 | **<0.0001** | -0.07 | **0.0013** | -0.12 | **<0.0001** | -0.01 | 0.82 |
| CTQ | 0.017 | 0.78 | 0.01 | 0.84 | -0.30 | **<0.0001** | -0.04 | 0.52 | -0.24 | **<0.0001** | 0.16 | **0.005** | 0.15 | **0.010** | 0.24 | **<0.0001** | 0.23 | **<0.0001** |
|  | ***t*** | ***p*** | ***t*** | ***p*** | ***t*** | ***p*** | ***t*** | ***p*** | ***t*** | ***p*** | ***t*** | ***p*** | ***t*** | ***p*** | ***t*** | ***p*** | ***t*** | ***p*** |
| History Psychosis | 0.29 | 0.77 | 1.26 | 0.21 | 0.20 | 0.84 | 0.57 | 0.57 | 0.02 | 0.98 | 0.15 | 0.88 | 0.48 | 0.63 | 1.21 | 0.23 | 0.29 | 0.77 |
| BD Subtype (I v II) | 2.16 | **0.03** | 0.23 | 0.82 | 2.19 | **0.03** | 0.85 | 0.40 | 1.02 | 0.31 | 1.95 | **0.05** | 1.76 | 0.08 | 2.09 | **0.04** | 2.16 | **0.03** |
|  |  |  |  |  |  |  |  |  |  |  |  |  |  |  |  |  |  |  |

*Note*. r/rpb = Pearson’s r or point biserial correlation, BMI = Body Mass Index. The number of psychiatric hospitalizations and total manic, hypomanic, and depressive episodes were transformed using inverse hyperbolic transformation to correct positive skew. CTQ = Childhood Trauma Questionnaire.

# Supplementary Table 36. Group differences between illness course measures, BD subtype, BMI and psychotropic medication use in BD patients

|  | **History Psychosis** | |  | **BD Type** | |  | **Manic Episodes** | |  | **Hypomanic Episodes** | | |  | **BMI** | |
| --- | --- | --- | --- | --- | --- | --- | --- | --- | --- | --- | --- | --- | --- | --- | --- |
|  | **x^2^** | **p** | | **x^2^** | **p** | | **t** | **p** | | **t** | **p** | | | **t** | **p** |
| **AED** | 24.618 | **<0.0001** | | 21.684 | **<0.0001** | | 1.9575 | 0.051 | | 0.013047 | 0.99 | | | -2.8855  (BMI ↑) | **0.004** |
|  | (Psychosis ↓) |  |  | (BD1 ↑) |  |  |  |  |  |  |  |  |  |  |  |
| **AD** | 4.0114 | **0.045** | | 18.04 | **0.0001** | | 1.9276 | 0.054 | | -0.10382 | 0.92 | | | -3.1479 (BMI ↑) | **0.0017** |
|  | (Psychosis ↓) |  |  | (BD1 ↓) |  |  |  |  |  |  |  |  |  |  |  |
| **Li** | 22.832 | **<0.0001** | | 84.647 | **<0.0001** | | -3.2591 | **0.0012** | | -3.5727 | **0.0004** | | | -0.22918 | 0.82 |
|  | (Psychosis ↓) |  |  | (BD1 ↑) |  |  | (Mania ↓) |  |  | (Hypomania ↓) |  |  |  |  |  |
| **AP** | 40.602 | **<0.0001** | | 83.005 | **<0.0001** | | -0.20934 | 0.83 | | -2.7203 | **0.0067** | | | -2.926 (BMI ↑) | **0.0035** |
|  | (Psychosis ↑) |  |  | (BD1 ↑) |  |  |  |  |  | (Hypomania ↓) |  |  |  |  |  |
|  | **Depressive Episodes** | |  | **Illness Duration** | |  | **Psychiatric Hospitalizations** | |  | **Age of Onset** | |  | | **CTQ** | |
|  | **t** | **p** | | **t** | **p** | | **t** | **p** | | **t** | **p** | | | **t** | **p** |
| **AED** | 2.3777 | **0.018** | | 2.3777 | **0.018** | | 3.328 | **0.001** | | 2.0115 | **0.044** | | | -0.60324 | 0.55 |
|  | (Depression ↑) |  |  | (Duration ↑) |  |  | (Hospitalizations ↑) |  |  | (Onset ↑) |  |  |  |  |  |
| **AD** | 4.3551 | **<0.0001** | | 4.3551 | **<0.0001** | | 3.9004 | **0.0001** | | 3.3435 | **0.0008** | | | -1.42 | 0.16 |
|  | (Depression ↑) |  |  | (Duration ↑) |  |  | (Hospitalizations ↑) |  |  | (Onset ↑) |  |  |  |  |  |
| **Li** | 3.6508 | **0.0003** | | 3.6508 | **0.0003** | | 3.4704 | **0.0006** | | 4.465 | **<0.0001** | | | -1.3672 | 0.17 |
|  | (Depression ↑) |  |  | (Duration ↑) |  |  | (Hospitalizations ↑) |  |  | (Onset ↑) |  |  |  |  |  |
| **AP** | -3.421 | **0.0006** | | -3.421 | **0.0006** | | 5.667 | **<0.0001** | | 0.94201 | 0.35 | | | -2.6579 (CTQ ↑) | **0.0082** |
|  | (Depression ↓) |  |  | (Duration ↓) |  |  | (Hospitalizations ↑) |  |  |  |  |  |  |  |  |

*Note*. AED = Antiepileptics, AD = Antidepressants, Li = Lithium, AP = Antipsychotics, x^2^ = chi-square test statistic, CTQ = Childhood Trauma Questionnaire, BMI = Body Mass index. Arrows indicate the direction of association for the medication group relative to the comparison group: ↑ = higher values or greater likelihood; ↓ = lower values or reduced likelihood.

# Supplementary Table 37. The moderating role of psychotropic drugs on the relationship between total manic episodes and subcortical volume in BD patients

|  | **β** | **SE** | **t-statistic** | **β 95% CI** | **p-value** | **q-value** |
| --- | --- | --- | --- | --- | --- | --- |
| ***Hippocampus*** |  |  |  |  |  |  |
| Antiepileptics | 0.00 | 0.00 | 1.29 | [.00, .01] | 0.20 | 0.26 |
| Lithium | 0.00 | 0.00 | -0.23 | [-.01, .01] | 0.82 | 0.55 |

*Note.* β = beta coefficient, CI = Confidence Intervals, SE= Standard Error. In each model we controlled for sex, age, ICV (for subcortical structures) and other concurrently used psychotropic medications.

# Supplementary Table 38. The moderating role of psychotropic drugs on the relationship between total hypomanic episodes and subcortical volume in BD patients

|  | **β** | **SE** | **t-statistic** | **β 95% CI** | **p-value** | **q-value** |
| --- | --- | --- | --- | --- | --- | --- |
| ***Thalamus*** |  |  |  |  |  |  |
| Antiepileptics | 0.00 | 0.00 | -0.17 | [-.01, .01] | 0.87 | 0.56 |
| Lithium | 0.00 | 0.01 | -0.49 | [-.02, .01] | 0.62 | 0.49 |
| Antipsychotics | 0.00 | 0.01 | -0.01 | [-.01, .01] | 0.99 | 0.60 |
| ***Putamen*** |  |  |  |  |  |  |
| Antiepileptics | 0.00 | 0.00 | 0.08 | [-.01, .01] | 0.94 | 0.58 |
| ***Accumbens*** |  |  |  |  |  |  |
| Antiepileptics | 0.00 | 0.00 | 1.098 | [.00, .01] | 0.27 | 0.31 |

*Note.* β = beta coefficient, CI = Confidence Intervals, SE= Standard Error. In each model we controlled for sex, age, ICV (for subcortical structures) and other concurrently used psychotropic medications.

# Supplementary Table 39. The moderating role of psychotropic drugs on the relationship between total depressive episodes and subcortical volume in BD patients

|  | **β** | **SE** | **t-statistic** | **β 95% CI** | **p-value** | **q-value** |
| --- | --- | --- | --- | --- | --- | --- |
| ***Hippocampus*** |  |  |  |  |  |  |
| Antiepileptics | 0.00 | 0.00 | 0.54 | [.00, .01] | 0.59 | 0.47 |
| Lithium | 0.00 | 0.00 | -0.09 | [-.01, .01] | 0.43 | 0.40 |
| ***Thalamus*** |  |  |  |  |  |  |
| Antiepileptics | 0.00 | 0.00 | -0.55 | [-.01, .00] | 0.58 | 0.47 |
| Lithium | 0.00 | 0.00 | 0.48 | [.00, .01] | 0.63 | 0.49 |
| Antipsychotics | -0.0016 | 0.0023 | -0.711 | [-.01, .00] | 0.48 | 0.44 |

*Note.* β = beta coefficient, CI = Confidence Intervals, SE= Standard Error. In each model we controlled for sex, age, ICV (for subcortical structures) and other concurrently used psychotropic medications

# Supplementary Table 40. The moderating role of psychotropic drugs on the relationship between total psychiatric hospitalizations and subcortical volume in BD patients

|  | **β** | **SE** | **t-statistic** | **β 95% CI** | **p-value** | **q-value** |
| --- | --- | --- | --- | --- | --- | --- |
| ***Thalamus*** |  |  |  |  |  |  |
| Antiepileptics | -0.01 | 0.02 | -0.45 | [-.05, .03] | 0.65 | 0.49 |
| Lithium | 0.04 | 0.03 | 1.41 | [-.01, .09] | 0.16 | 0.25 |
| Antipsychotics | 0.02 | 0.02 | 1.01 | [-.02, .06] | 0.31 | 0.33 |
| ***Putamen*** |  |  |  |  |  |  |
| Antiepileptics | 0.02 | 0.02 | 1.21 | [-.01, .06] | 0.23 | 0.28 |
| **Caudate** |  |  |  |  |  |  |
| Antiepileptics | 0.02 | 0.02 | 0.83 | [-.03, .06] | 0.41 | 0.40 |

*Note.* β = beta coefficient, CI = Confidence Intervals, SE= Standard Error. In each model we controlled for sex, age, ICV (for subcortical structures) and other concurrently used psychotropic medications.

# Supplementary Table 41. The moderating role of psychotropic drugs on the relationship between a history of psychosis and subcortical volume in BD patients

|  | **β** | **SE** | **t-statistic** | **β 95% CI** | **p-value** | **q-value** |
| --- | --- | --- | --- | --- | --- | --- |
| ***Hippocampus*** |  |  |  |  |  |  |
| Antiepileptics | 0.06 | 0.08 | 0.68 | [-.10, .21] | 0.50 | 0.44 |
| Lithium | 0.10 | 0.08 | 1.17 | [-.06, .26] | 0.24 | 0.29 |

*Note.* β = beta coefficient, CI = Confidence Intervals, SE= Standard Error. In each model we controlled for sex, age, ICV (for subcortical structures) and other concurrently used psychotropic medications.

# Supplementary Table 42. The moderating role of psychotropic drugs on the relationship between age of onset and subcortical volume in BD patients

|  | **β** | **SE** | **t-statistic** | **β 95% CI** | **p-value** | **q-value** |
| --- | --- | --- | --- | --- | --- | --- |
| ***Ventricles*** |  |  |  |  |  |  |
| Antiepileptics | 0.00 | 0.00 | 0.88 | [.00, .01] | 0.38 | 0.38 |
| ***Hippocampus*** |  |  |  |  |  |  |
| Antiepileptics | 0.00 | 0.00 | -0.59 | [-.01, .00] | 0.55 | 0.46 |
| Lithium | 0.00 | 0.00 | 0.39 | [-.01, .01] | 0.70 | 0.51 |
| ***Thalamus*** |  |  |  |  |  |  |
| Antiepileptics | 0.00 | 0.00 | 0.157 | [-.01, .01] | 0.87 | 0.56 |
| Lithium | 0.00 | 0.00 | 0.273 | [-.01, .01] | 0.78 | 0.54 |
| Antipsychotics | 0.00 | 0.00 | 0.61 | [.00, .01] | 0.54 | 0.46 |
| ***Caudate*** |  |  |  |  |  |  |
| Antiepileptics | 0.00 | 0.00 | 0.82 | [.00, .01] | 0.41 | 0.40 |
| ***Putamen*** |  |  |  |  |  |  |
| Antiepileptics | 0.00 | 0.00 | 0.585 | [.00, .01] | 0.56 | 0.46 |
| ***Accumbens*** |  |  |  |  |  |  |
| Antiepileptics | 0.00 | 0.00 | -0.20 | [-.01, .01] | 0.85 | 0.55 |

*Note.* β = beta coefficient, CI = Confidence Intervals, SE= Standard Error. In each model we controlled for sex, age, ICV (for subcortical structures) and other concurrently used psychotropic medications.

# Supplementary Table 43. The moderating role of psychotropic drugs on the relationship between illness duration and subcortical volume in BD patients

|  | **β** | **SE** | **t-statistic** | **β 95% CI** | **p-value** | **q-value** |
| --- | --- | --- | --- | --- | --- | --- |
| ***Ventricles*** |  |  |  |  |  |  |
| Antiepileptics | 0.00 | 0.00 | -0.17 | [-.01, .01] | 0.87 | 0.56 |
| ***Hippocampus*** |  |  |  |  |  |  |
| Antiepileptics | 0.00 | 0.00 | -0.27 | [-.01, .01] | 0.79 | 0.54 |
| Lithium | 0.00 | 0.00 | 1.34 | [.00, .01] | 0.18 | 0.26 |
| ***Thalamus*** |  |  |  |  |  |  |
| Antiepileptics | 0.00 | 0.00 | -0.781 | [-.01, .00] | 0.43 | 0.41 |
| Lithium | 0.00 | 0.00 | 1.624 | [.00, .01] | 0.10 | 0.18 |
| Antipsychotics | 0.01 | 0.00 | 1.74 | [.00, .01] | 0.08 | 0.16 |
| ***Caudate*** |  |  |  |  |  |  |
| Antiepileptics | 0.00 | 0.00 | -1.45 | [.00, .01] | 0.15 | 0.24 |
| ***Putamen*** |  |  |  |  |  |  |
| Antiepileptics | -0.01 | 0.00 | -1.756 | [-.01, .00] | 0.08 | 0.16 |
| ***Accumbens*** |  |  |  |  |  |  |
| Antiepileptics | 0.00 | 0.00 | -0.40 | [-.01, .00] | 0.69 | 0.51 |

*Note.* β = beta coefficient, CI = Confidence Intervals, SE= Standard Error. In each model we controlled for sex, age, ICV (for subcortical structures) and other concurrently used psychotropic medications.

# Supplementary Table 44. The moderating role of psychotropic drugs on the relationship between BD subtype I vs II and subcortical volume in BD patients

|  | **β** | **SE** | **t-statistic** | **β 95% CI** | **p-value** | **q-value** |
| --- | --- | --- | --- | --- | --- | --- |
| ***Ventricles*** |  |  |  |  |  |  |
| Antiepileptics | -0.12 | 0.10 | -1.23 | [-.30, .07] | 0.21 |  |
| ***Thalamus*** |  |  |  |  |  |  |
| Antiepileptics | -0.02 | 0.07 | -0.26 | [-.17 .13] | 0.79 |  |
| Lithium | 0.01 | 0.09 | 0.16 | [.00, .01] | 0.87 |  |
| Antipsychotics | 0.08 | 0.09 | 0.87 | [-0.09, .24] | 0.38 |  |

*Note.* β = beta coefficient, CI = Confidence Intervals, SE= Standard Error. In each model we controlled for sex, age, ICV (for subcortical structures) and other concurrently used psychotropic medications.

# Supplementary Table 45. Regional variation in lithium, antiepileptic, antipsychotic, and antidepressant use among patients with bipolar disorder

| **Drug** | **Europe** | **North America** | **Rest of world** | **N_total** | **χ²(df)** | **p** | **Cramér’s V** | **Interpretation** |
| --- | --- | --- | --- | --- | --- | --- | --- | --- |
| Lithium | 562 (44.3%) | 162 (21.4%) | 146 (36.8%) | 2424 | χ²(2)=108 | <.001 | 0.21 | small–medium |
| AED | 483 (38.1%) | 237 (31.3%) | 205 (52.8%) | 2414 | χ²(2)=50 | <.001 | 0.14 | small |
| AP | 493 (38.9%) | 290 (38.3%) | 225 (56.7%) | 2423 | χ²(2)=45 | <.001 | 0.14 | small |
| AD | 500 (39.4%) | 220 (29.1%) | 120 (30.9%) | 2414 | χ²(2)=23 | <.001 | 0.1 | small |

*Note*. AED = Antiepileptics, AD = Antidepressants, Li = Lithium, AP = Antipsychotics, x^2^ = chi-square test statistic. Rest of world includes sites outside Europe and North America, comprising Australia, Asia, and South America.

1. Greifer, N. (2020). *A guide to using WeightIt for estimating balancing weights*. [↑](#footnote-ref-1)
2. Pustejovsky, J. E., & Tipton, E. (2018). Small-sample methods for cluster-robust variance estimation and hypothesis testing in fixed effects models. *Journal of Business & Economic Statistics*, *36*(4), 672-683. [↑](#footnote-ref-2)
3. The reported p-values are uncorrected for multiple comparisons [↑](#footnote-ref-3)
4. The reported p-values are uncorrected for multiple comparisons [↑](#footnote-ref-4)
5. The reported p-values are uncorrected for multiple comparisons [↑](#footnote-ref-5)
6. The reported p-values are uncorrected for multiple comparisons [↑](#footnote-ref-6)
7. The reported p-values are uncorrected for multiple comparisons [↑](#footnote-ref-7)
8. The reported p-values are uncorrected for multiple comparisons [↑](#footnote-ref-8)
